# Using phylogenetically-informed annotation (PIA) to search for light-interacting genes in transcriptomes from non-model organisms

**DOI:** 10.1186/s12859-014-0350-x

**Published:** 2014-11-19

**Authors:** Daniel I Speiser, M Sabrina Pankey, Alexander K Zaharoff, Barbara A Battelle, Heather D Bracken-Grissom, Jesse W Breinholt, Seth M Bybee, Thomas W Cronin, Anders Garm, Annie R Lindgren, Nipam H Patel, Megan L Porter, Meredith E Protas, Ajna S Rivera, Jeanne M Serb, Kirk S Zigler, Keith A Crandall, Todd H Oakley

**Affiliations:** Department of Ecology, Evolution, and Marine Biology, University of California Santa Barbara, Santa Barbara, CA USA; Department of Biological Sciences, University of South Carolina, Columbia, SC USA; The Whitney Laboratory for Marine Bioscience, University of Florida, St. Augustine, FL USA; Department of Biological Sciences, Florida International University-Biscayne Bay Campus, North Miami, FL USA; Florida Museum of Natural History, University of Florida, Gainesville, FL USA; Department of Biology, Brigham Young University, Provo, UT USA; Department of Biological Sciences, University of Maryland Baltimore County, Baltimore, MD USA; Department of Biology, Marine Biological Section, University of Copenhagen, Copenhagen, Denmark; Department of Biology, Portland State University, Portland, OR USA; Department of Molecular and Cell Biology & Department of Integrative Biology, University of California, Berkeley, CA USA; Department of Biology, University of South Dakota, Vermillion, SD USA; Department of Natural Sciences and Mathematics, Dominican University of California, San Rafael, CA USA; Department of Biology, University of the Pacific, Stockton, CA USA; Department of Ecology, Evolution, and Organismal Biology, Iowa State University, Ames, IA USA; Department of Biology, Sewanee: The University of the South, Sewanee, TN USA; Computational Biology Institute, George Washington University, Ashburn, VA USA; Department of Invertebrate Zoology, National Museum of Natural History, Smithsonian Institution, Washington, DC USA

**Keywords:** Bioinformatics, Eyes, Evolution, Galaxy, Next-generation sequence analysis, Orthology, Phototransduction, Transcriptomes, Vision

## Abstract

**Background:**

Tools for high throughput sequencing and *de novo* assembly make the analysis of transcriptomes (*i.e.* the suite of genes expressed in a tissue) feasible for almost any organism. Yet a challenge for biologists is that it can be difficult to assign identities to gene sequences, especially from non-model organisms. Phylogenetic analyses are one useful method for assigning identities to these sequences, but such methods tend to be time-consuming because of the need to re-calculate trees for every gene of interest and each time a new data set is analyzed. In response, we employed existing tools for phylogenetic analysis to produce a computationally efficient, tree-based approach for annotating transcriptomes or new genomes that we term Phylogenetically-Informed Annotation (PIA), which places uncharacterized genes into pre-calculated phylogenies of gene families.

**Results:**

We generated maximum likelihood trees for 109 genes from a Light Interaction Toolkit (LIT), a collection of genes that underlie the function or development of light-interacting structures in metazoans. To do so, we searched protein sequences predicted from 29 fully-sequenced genomes and built trees using tools for phylogenetic analysis in the Osiris package of Galaxy (an open-source workflow management system). Next, to rapidly annotate transcriptomes from organisms that lack sequenced genomes, we repurposed a maximum likelihood-based Evolutionary Placement Algorithm (implemented in RAxML) to place sequences of potential LIT genes on to our pre-calculated gene trees. Finally, we implemented PIA in Galaxy and used it to search for LIT genes in 28 newly-sequenced transcriptomes from the light-interacting tissues of a range of cephalopod mollusks, arthropods, and cubozoan cnidarians. Our new trees for LIT genes are available on the Bitbucket public repository (http://bitbucket.org/osiris_phylogenetics/pia/) and we demonstrate PIA on a publicly-accessible web server (http://galaxy-dev.cnsi.ucsb.edu/pia/).

**Conclusions:**

Our new trees for LIT genes will be a valuable resource for researchers studying the evolution of eyes or other light-interacting structures. We also introduce PIA, a high throughput method for using phylogenetic relationships to identify LIT genes in transcriptomes from non-model organisms. With simple modifications, our methods may be used to search for different sets of genes or to annotate data sets from taxa outside of Metazoa.

**Electronic supplementary material:**

The online version of this article (doi:10.1186/s12859-014-0350-x) contains supplementary material, which is available to authorized users.

## Background

An integrated understanding of the function and evolution of complex biological traits – such as eyes – is a major goal for biologists. Ideally, we will learn how specific genes evolved to influence particular phenotypes at multiple levels of organization. Eyes are an excellent system for establishing causative links between genotype and phenotype because their genetic components tend to be well-characterized and deeply conserved [1-4]. However, we lack genomic or transcriptomic resources for many species that are amenable to the physiological, developmental, or evolutionary study of eyes and vision. New tools for high throughput sequencing (*e.g.* 454, Illumina, SOLiD) and *de novo* assembly provide a solution to this problem, as they make the development of transcriptomic resources feasible for almost any organism, even invertebrate animals where few full genomes are available relative to species diversity [5].

A remaining challenge is that it can be difficult to assign identities to the sequences that comprise transcriptomes from non-model organisms. Existing methods for annotating transcriptomes – *e.g.* Blast2GO [6], GOtcha [7], GoFigure [8], OntoBlast [9], and AutoFACT [10] – tend to rely upon similarities between new sequences and previously characterized genes, an approach which can give misleading results because there is no consistent method for predicting how similar an uncharacterized gene must be to a characterized one to share a common function. Phylogenetic analyses provide a more objective way to annotate transcriptomes: if a sequence falls in a clade of genes whose functions are characterized and similar to each other, we can use parsimony to infer that the sequence has a similar function. A draw-back to phylogenetic analyses is that they tend to be time-consuming because of the need to re-calculate trees each time that new data are collected (*e.g.* [3]).

In response, we used existing tools for phylogenetic analysis in the Osiris package [11] of Galaxy [12-14] – an open-source workflow management system – to produce a computationally efficient, tree-based approach for annotating transcriptomes that we term Phylogenetically-Informed Annotation (PIA). First, we used tools in Galaxy and protein sequences predicted from 29 fully-sequenced genomes to produce trees for 109 gene families from a metazoan Light-Interaction Toolkit (LIT 1.0), *i.e.* a set of genetic components that metazoans use to build eyes and other light-interacting structures. LIT 1.0 includes genes that animals use to detect light (*e.g.* opsins and cryptochromes; [15,16]), absorb light (*e.g.* pigment synthesis enzymes; [17]), and refract light (*e.g.* lens crystallins; [18,19]), as well as transcription factors associated with the development of eyes and other light-interacting structures (*e.g. Pax6*; [20,21]). Second, we designed a workflow in Galaxy that uses e-values from BLAST [22] to identify potential homologs of LIT genes. The workflow then employs a choice of multiple sequence alignment programs (MUSCLE [23] or MAFFT [24,25]) and a repurposed Evolutionary Placement Algorithm (implemented in RAxML; [26,27]) to place these sequences using Maximum Likelihood on to the trees that we calculated earlier for genes from LIT 1.0. Finally, we tested our approach by using PIA to search for LIT genes in 28 new transcriptomes that we generated using the Roche 454 platform. These transcriptomes – generated from RNA expressed in light-interacting tissues from a range of cephalopod mollusks, arthropods, and cubozoan cnidarians – are all from animals that are not traditional model organisms, but are well-suited for answering particular questions about the function and evolution of eyes and other light-interacting structures.

## Implementation

Here, we describe the implementation of Phylogenetically-Informed Annotation (PIA), a new approach for using phylogenetic methods to rapidly annotate transcriptomes from non-model organisms. We focus on a set of 109 genes that we selected to form a metazoan Light Interaction Toolkit (LIT). We make LIT-PIA available through an instance of Galaxy on a publicly accessible web server (http://galaxy-dev.cnsi.ucsb.edu/pia/). Users can find a written tutorial and a screencast demonstration linked on that site. In Galaxy, we implemented a tool called pia, which executes a perl script that calls a series of bioinformatics tools, including BLAST [22], a choice of multiple sequence alignment programs (MUSCLE [23] or MAFFT [24,25]) and RAxML [27,28]. Implementing PIA in Galaxy allows us to integrate the pia tool with other useful tools, such as tab2trees of the Osiris package [11], which visualizes multiple phylogenetic trees in a single PDF file. Furthermore, the Galaxy instance allows for user-friendly annotation of LIT genes using PIA. Our pre-calculated trees for LIT genes can be selected from a menu on our Galaxy pia tool, and all the gene trees are also available on the Bitbucket public repository and documentation for using the public website is available there (http://bitbucket.org/osiris_phylogenetics/pia/src/) in the docs subdirectory.

## Results and discussion

### New trees for 109 LIT genes

We generated maximum likelihood trees for 109 genes from a metazoan Light Interaction Toolkit (LIT 1.0; Additional file 1: Table S1; Additional file 2). From our efforts, we noted that many LIT genes do not have orthologous relationships across Metazoa. For example, we find that LIT genes with similar functions in distantly related taxa (*e.g.* arthropods and vertebrates) are often paralogs, not orthologs, due to lineage-specific gene duplications. Although evidence suggests that orthologs tend to be more similar functionally than paralogs, this does not hold true in the case of all gene families [26,27]. Thus, we conclude that tree-building is a useful approach for inferring the function of certain LIT genes, yet we still urge researchers to use caution when assigning functions to uncharacterized genes in the absence of functional tests and based on phylogenetic relationships alone. Further, how one selects the sequences used to build a tree may have a significant influence on the inferences drawn about the ancestral function and patterns of diversification of gene families. Thus, we advocate for an objective, repeatable approach to choosing sequences for gene trees (such as the one we employ here), especially when using phylogenetic relationships between these sequences to make inferences about the functions of newly sequenced genes.

### New transcriptomes for integrative and comparative vision research

We generated 28 transcriptomes for light-interacting tissues from a range of cephalopod mollusks, arthropods, and cubozoan cnidarians (Table 1). We sequenced transcriptomes from these taxa because they lacked genomic or transcriptomic resources, but are well-suited for answering certain questions about the function, development, and evolution of eyes and other light-interacting structures. The sizes of our transcriptomes varied (Additional file 3: Table S2). For example, the number of total bases in our transcriptomes averaged 2,903,000 ± 1,185,000 (mean ± std deviation) with a low of 89,000 bp (for an eye from the cephalopod *Vampyroteuthis infernalis*) and a high of 5,185,000 bp (for a ventral eye from the horseshoe crab *Limulus polyphemus*). The number of isotigs per transcriptome ranged from 168 (*V. infernalis* eye dataset) to 5,447 (for tissue from the eyes and head of the isopod *Asellus aquaticus*) and the mean sizes of isotigs ranged from 530 bp (*V. infernalis* eye dataset) to 1,397 bp (*L. polyphemus* ventral eye dataset).

**Table 1:** **Collection data for the samples from which we generated 28 new transcriptomes**

	**Species**	**Description**	**Tissue**	**Collection**	**Location**	**Lat.**	**Long.**	**Depth (m)**
1	*Chiroteuthis calyx*	Cephalopod (squid)	Adult eye tissue	ROV (MBARI)	CA, USA	36°69'N	122°05'W	446
2	*Euprymna scolopes*	Cephalopod (squid)	Adult eye tissue	Hand net	Honolulu, HI, USA	21°27'N	157°77'W	0.5
3	*Galiteuthis armata*	Cephalopod (squid)	Adult eye tissue	ROV (MBARI)	CA, USA	36°69'N	122°05'W	556
4	*Octopus bimaculoides*	Cephalopod (octopus)	Adult dermal tissue	SCUBA	Santa Barbara, CA, USA	34°43'N	119°71'W	?
5	*Uroteuthis edulis*	Cephalopod (squid)	Adult eye tissue	Fishmarket	Numazu, Japan	35°08'N	138°86'E	?
6	*Vampyroteuthis infernalis*	Cephalopod (vampire squid)	Adult eye tissue	ROV (MBARI)	CA, USA	36°08'N	122°30'W	1096
7	*Asellus aquaticus*	Arthropod (cave isopod)	Adult head	Hand net	Planina cave, Slovenia	45°82'N	14°25'E	65
8	*Asellus aquaticus*	Arthropod (surface isopod)	Embryos and hatchlings	Lab colony	Planina cave, Slovenia	45°82'N	14°25'E	N/A
9	*Asellus aquaticus*	Arthropod (hybrid isopod)	Adult head	Lab colony	Planina cave, Slovenia	45°82'N	14°25'E	N/A
10	*Asellus aquaticus*	Arthropod (surface isopod)	Adult head	Lab colony	Planina cave, Slovenia	45°82'N	14°25'E	N/A
11	*Benthesicymus bartletti*	Arthropod (shrimp)	Adult eye tissue	Benthic skimmer	Northern Gulf of Mexico	28°48’N	88°12’W	1350
12	*Caecidotea bicrenata*	Arthropod (cave isopod)	Adult head	Hand net	Franklin County, TN, USA	35°15'N	86°10'W	0.1
13	*Caecidotea bicrenata*	Arthropod (cave isopod)	Whole embryos	Hand net	Franklin County, TN, USA	35°15'N	86°10'W	0.1
14	*Caecidotea forbesi*	Arthropod (surface isopod)	Adult head	Hand net	Sewanee, TN, USA	35°22'N	85°97'W	0.5
15	*Caecidotea forbesi*	Arthropod (surface isopod)	Whole embryos	Hand net	Sewanee, TN, USA	35°22'N	85°97'W	0.5
16	*Euphilomedes carcharodonta*	Arthropod (ostracod)	Whole embryos	Hand net	Half Moon Bay, CA, USA	37°29'N	122°29'W	1
17	*Hemisquilla californiensis*	Arthropod (stomatopod, male)	Adult eye tissue	Dredge	Orange County, CA, USA	33°67'N	117°78'W	?
18	*Ischnura ramburii*	Arthropod (damselfly, female)	Adult head	Hand net	Austin, TX, USA	30°28'N	97°78'W	N/A
19	*Limulus polyphemus*	Arthropod (horseshoe crab)	Adult lateral eye	Hand	Indian River near Titusville, FL, USA	28°74'N	80°75'W	Surface
20	*Limulus polyphemus*	Arthropod (horseshoe crab)	Adult median eye	Hand	Indian River near Titusville, FL, USA	28°74'N	80°75'W	Surface
21	*Limulus polyphemus*	Arthropod (horseshoe crab)	Adult ventral eye	Hand	Indian River near Titusville, FL, USA	28°74'N	80°75'W	Surface
22	*Procambarus alleni*	Arthropod (crayfish)	Adult eye tissue	Hand net	Fisheating Creek, Glades County, FL, USA	26°90'N	81°24'W	Surface
23	*Procambarus franzi*	Arthropod (crayfish)	Adult eye tissue	Hand net	Orange Lake Cave, Marion County, FL, USA	Contact Authors	Contact Authors	Surface
24	*Pseudosquilla ciliata*	Arthropod (stomatopod)	Adult eye tissue	Dredge	Isla Magueyes, Puerto Rico	17°97'N	67°05'W	?
25	*Systellaspis debilis*	Arthropod (shrimp)	Adult eye tissue	Benthic skimmer	Nothern Gulf of Mexico	28°48’N	88°12’W	1350
26	*Telebasis salva*	Arthropod (damselfly)	Juvenile head	Hand net	Austin, TX, USA	30°28'N	97°78'W	N/A
27	*Tripedalia cystophora*	Cnidarian (cubozoan)	Adult rhopalia	Snorkel	La Parguera, Puerto Rico	17°58'N	67°04'W	Surface
28	*Tripedalia cystophora*	Cnidarian (cubozoan)	Whole planula larvae	Snorkel	La Parguera, Puerto Rico	17°58'N	67°04'W	Surface

### Phylogenetically-informed annotation

**Figure 1:**
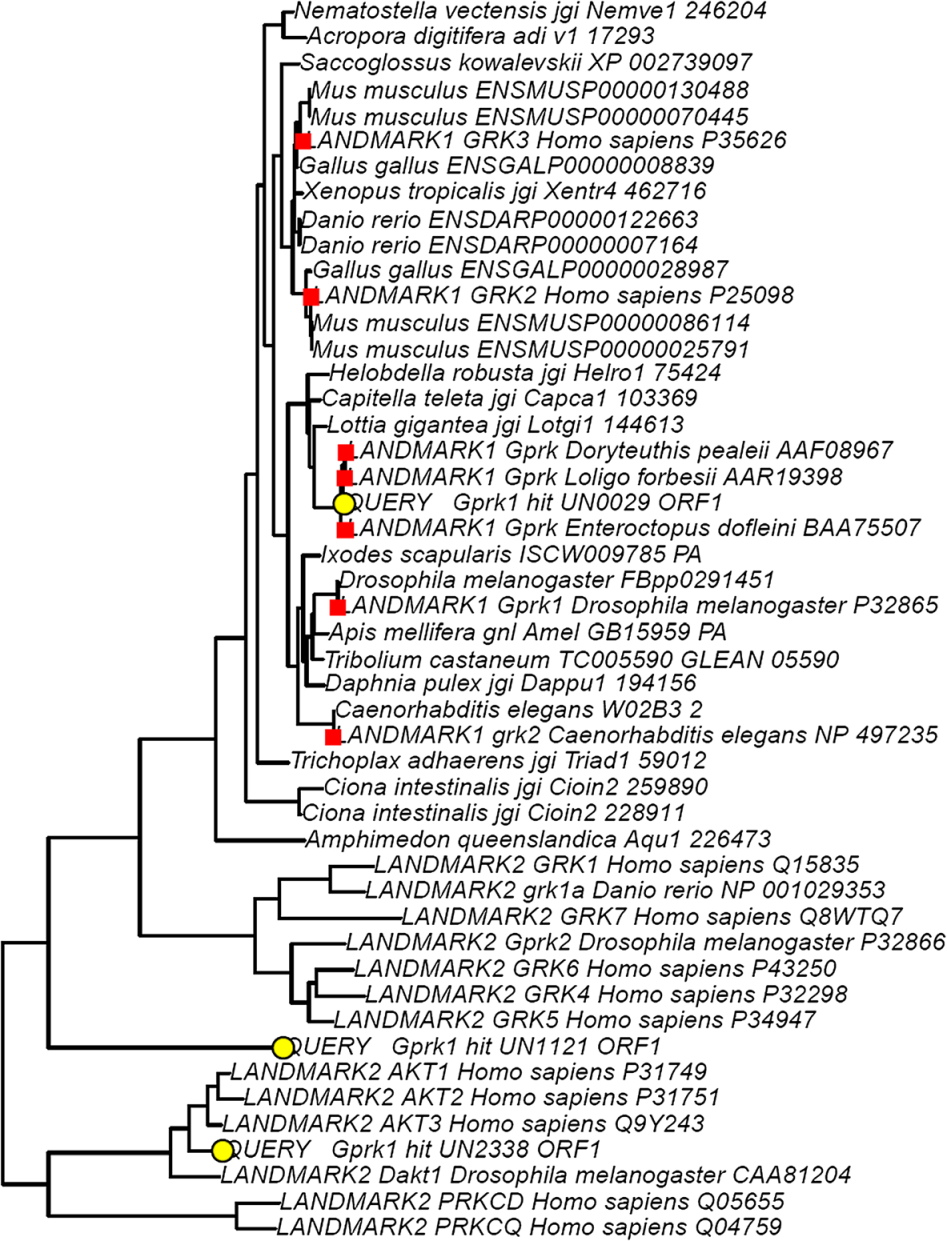
**An example of results from phylogenetically-informed annotation (PIA).** Here, we search a transcriptome generated for an eye from the squid *Chiroteuthis calyx* for relatives of the gene G protein-coupled receptor kinase 1 (*Gprk1*), a component of the rhabdomeric phototransduction pathway. Sequences marked with red squares and labeled "LANDMARK1" are homologs of *Gprk1* that have been well-characterized functionally and are thought to share similar functions. In contrast, sequences labeled "LANDMARK2" are well-characterized genes that are more distantly related to *Gprk1*. Sequences marked with yellow circles and labeled "QUERY" are protein sequences predicted from our transcriptome for *C. calyx*. Based on their phylogenetic positions and branch lengths, it is likely that one hit (UN0029) represents an ortholog of *Gprk1* and that two hits (UN1121 and UN2338) represent genes that are distant paralogs of *Gprk1*. We conclude that the eyes of *C. calyx* express an ortholog of *Gprk1*, a component of the rhabdomeric phototransduction pathway.

To rapidly identify potential LIT 1.0 genes in our genetic datasets, we implemented PIA in Galaxy. Unlike past efforts at using phylogenetic methods to annotate transcriptomes, PIA does not require the re-calculation of gene trees every time a new sequence is to be analyzed. The output from PIA is a gene tree or a set of gene trees in Newick format that can be viewed using existing tools in Galaxy, such as tab2trees from the Osiris package [11]. These trees include sequences identified from predicted protein databases associated with 29 fully-sequenced genomes (Additional file 4: Table S3 and Additional file 5: Supplementary References for Table S3). The trees also include sequences marked as either Landmarks or Queries (Figure 1). Landmarks are genes (usually from model organisms) whose functions and/or patterns of expression have been characterized relatively well. Sequences marked "LANDMARK1" – which are highlighted with red squares when the trees are viewed using our tab2trees tool – are well-characterized LIT genes. Sequences marked "LANDMARK2" are also well-characterized genes, but are those that have functions different than the LIT genes that we are seeking. For example, we included certain non-opsin GPCRs in our trees for opsins, but we labeled them as LANDMARK2s because we have evidence that they are not involved in the detection of light. Queries marked “QUERY” – which are noted by yellow circles in the output from the tab2trees tool – represent potential LIT genes that PIA has identified from a particular genetic dataset. Promising queries from a transcriptome (*i.e.* ones that may represent orthologs of LIT genes) will tend to fall on short branches in phylogenetic positions that are sensible given established relationships between species (for an example, see the query tagged “Gprk1 hit UN0029 ORF1” in Figure 1). The output from PIA may also include query sequences that are close relatives, but not orthologs, of LIT genes (for examples, see the queries tagged “Gprk1 hit UN1121 ORF1” and “Gprk1 hit UN2338 ORF1” in Figure 1). Thus, we urge users of PIA to inspect carefully where queries fall on their respective gene trees and to make inferences about function accordingly.

Building additional gene trees will allow researchers to use PIA to search for LIT genes that are not included in our initial list or to search for new sets of genes. For example, there has been much recent interest in the sets of genes that underlie the process of biomineralization in animals [28,29]. Extensive databases of these genes have been published for invertebrate taxa (*e.g.* mollusks) that are not traditional model systems [30]. By building trees for these sets of genes and applying our methods for PIA, researchers will be able to survey new transcriptomes rapidly for genes that may be involved in biomineralization.

### The distribution of LIT genes across 28 new vision-related transcriptomes

**Figure 2:**
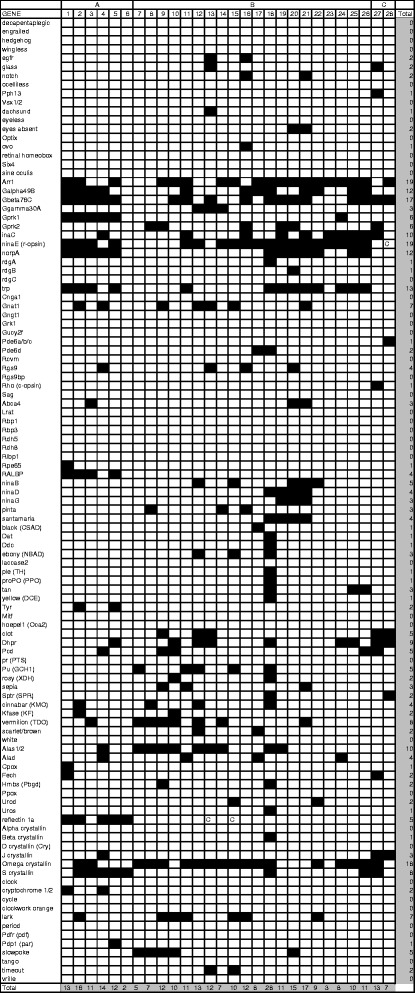
**The results of using phylogenetically-informed annotation (PIA) to search 28 new transcriptomes for light-interacting genes.** Here, cells shaded in black represent orthologs of LIT genes that are present in our transcriptomes. We have named genes based on conventions for *Drosophila melanogaster* whenever possible. Otherwise, gene names are given for *Mus musculus*. Cells marked "C" are hits from our transcriptomes that may represent contamination or assembly errors. The columns represent the following transcriptomes (where A = Cephalopods; B = Arthropods; and C = Cnidarians): 1 = *Chiroteuthis calyx* eye; 2 = *Euprymna scolopes* eye; 3 = *Galiteuthis armata* eye; 4 = *Octopus bimaculoides* skin; 5 = *Uroteuthis edulis* eye; 6 = *Vampyroteuthis infernalis* eye; 7 = *Asellus aquaticus* cave head; 8 = *Asellus aquaticus* embryos and hatchlings; 9 = *Asellus aquaticus* hybrid head; 10 = *Asellus aquaticus* surface head; 11 = *Benthesicymus bartletti* eye; 12 = *Caecidotea bicrenata* adult head; 13 = *Caecidotea bicrenata* embryos; 14 = *Caecidotea forbesi* adult head; 15 = *Caecidotea forbesi* embryos; 16 = *Euphilomedes carcharodonta* embryos; 17 = *Hemisquilla californiensis* eyes; 18 = *Ischnura ramburii* head; 19 *= Limulus polyphemus* lateral eye; 20 = *Limulus polyphemus* median eye; 21 = *Limulus polyphemus* ventral eye; 22 = *Procambarus alleni* eye; 23 = *Procambarus franzi* eye; 24 = *Pseudosquilla ciliata* eye; 25 = *Systellaspis debilis* eye; 26 = *Telebasis salva* head; 27 = *Tripedalia cystophora* eyes; 28 = *Tripedalia cystophora* planula larvae.

Across our 28 newly-sequenced transcriptomes, PIA identified potential orthologs of 69 of the 109 genes included in LIT 1.0 (Figure 2). We recovered certain genes from our transcriptomes far more often than others. Genes from LIT 1.0 that were expressed in ten or more of our transcriptomes included several components of the rhabdomeric phototransduction pathway, which is employed by the photoreceptors found in the eyes of many invertebrates [3,15]. These components include: Arrestin (*Arr*), Gq alpha (*Galpha49B*), Gq beta (*Gbeta76C*), protein kinase C (*inaC*), r-opsin (*ninaE*), phospholipase C (*norpA*), and transient receptor potential protein (*trp*). Ten or more of our transcriptomes also contained the enzyme aminolevulinate synthase (*Alas*), a component of the heme synthesis pathway [31], as well as an aldehyde dehydrogenase (*Aldh*) that is related to the Ω-crystallins expressed in the lenses of the camera eyes of cephalopods [32,33] and the mirror-based eyes of scallops [34-36]. Amino acid and nucleotide sequences for the potential LIT genes that we identified from our 28 transcriptomes, along with the corresponding gene trees, are available on our publicly-accessible web server (http://galaxy-dev.cnsi.ucsb.edu/pia/) under the Shared Data tab.

The transcriptomes that we generated for cephalopods contained between two (*Vampyroteuthis infernalis*) and sixteen (*Euprymna scolopes*) LIT genes (Figure 2). The majority of these genes represent components of the rhabdomeric phototransduction pathway, which is known to confer light-sensitivity to photoreceptors from the retinas of cephalopods [37-40]. These components include r-opsins, subunits of the hetero-trimeric Gq protein with which r-opsins interact, phospholipase C, and the ion channel TRP. We also recovered a number of lens crystallins, including relatives of the S- and Ω-crystallins indentified previously from the lenses of cephalopods [18]. Lastly, we found orthologs of LIT genes that are associated with two vision-related features that may be unique to the eyes of cephalopods. First, we found orthologs of the retinoid-binding protein RALBP, which is involved in regenerating the chromophores employed by the visual pigments of cephalopods [41,42]. Second, we found sequences that may represent reflectins, which are proteins that contribute to the biological mirrors that cephalopods use to camouflage their eyes [43,44].

Our transcriptomes from arthropods contained between three (*Procambarus franzi* – a crayfish) and 28 (*Ischnura ramburii –* a damselfly) potential orthologs of genes from LIT 1.0 (Figure 2). As in cephalopods, many of the genes we identified in arthropods represent components of the rhabdomeric phototransduction pathway. We also identified a number of genes that are associated with the synthesis of pterins and ommochromes, types of pigment found previously in the compound eyes of certain arthropods [45]. Additionally, several transcriptomes contained genes (*e.g. pinta, ninaG, ninaD, ninaB,* and *santamaria*) related to those that help synthesize the chromophores employed by the visual pigments in the eyes of the fruit fly *Drosophila melanogaster* [46,47].

Finally, our transcriptomes for the adult rhopalia and planula larvae of the cubozoan cnidarian *Tripedalia cystophora* contained thirteen and seven light-interacting genes, respectively (Figure 2). The majority of these genes are related to those associated with phototransduction in bilaterians. For example, we found a previously characterized opsin in our transcriptome for rhopalia from adult *T. cystophora* [48], as well as a Gs alpha subunit that is associated with light-detection in other cnidarians [49]. We also found evidence of J-crystallins, which are lens crystallins unique to the camera-type eyes of cubozoans [50].

It is important to note that the absence of a particular gene from a transcriptome is not necessarily informative. Even if a transcriptome is “complete”, it is only complete for a particular piece of tissue, from a particular animal, at a particular time. Thus, we have tried to draw general conclusions and points of future interest from the genes that we identified from our transcriptomes. We hope researchers will apply the approach that we have developed here to re-visit these light-interacting tissues and sequence more deeply and more broadly so that statistically meaningful comparisons of gene expression may be drawn between them.

## Conclusion

In this study, collaborators from multiple institutions worked together to produce new data and new approaches for studying genes expressed by eyes and other light-interacting tissues. We used high throughput sequencing to discover orthologs of light-interacting genes expressed in 28 vision-related tissues from a range of cephalopod mollusks, arthropods, and cnidarians. We have made available to vision researchers these genetic data, as well as new resources for analyzing high throughput genetic data. Specifically, we calculated trees to understand the evolutionary histories of 109 separate genes known to be involved with the function or development of light-interacting structures such as eyes. These trees can now be used to annotate transcriptomes by comparing the evolutionary similarities between newly identified sequences and genes that have been characterized previously through studies of their expression patterns and functions. These tools and analyses can be implemented by anyone using a set of online, flexible, user-friendly workflows implemented in Galaxy. These new data and tools will accelerate the understanding of genotype-phenotype connections and evolution in a diversity of animal visual systems.

## Methods

### Taxon selection

We sequenced 28 transcriptomes from 20 invertebrate taxa that lack genomic resources, but are well-suited for answering questions about the function, development, and evolution of eyes and other light-interacting structures (Table 1). For example, we generated transcriptomes from RNA expressed by the eyes and skin of certain **cephalopod mollusks (squid and octopus)**. These animals may have the most complex light-influenced behaviors of any invertebrate [51,52], but it appears that the eyes of cephalopods tend to contain only a single spectral class of photoreceptor ([53]; though see [54] as an exception). Additional physiological complexity may be suggested by the results of high throughput sequencing. It is also possible that certain visually-influenced behaviors in cephalopods – such as dynamic camouflage – may be influenced by molecular components that are expressed outside of their eyes. For example, past work suggests that certain cephalopods express LIT genes in their light-producing photophores [55] and in certain dermal cells [56].

We also sequenced transcriptomes for a range of **arthropods.** We chose to study **stomatopods (mantis shrimp)** because they have an unsurpassed ability to distinguish different aspects of light. Certain species are maximally sensitive to twelve distinct wavelength peaks and some species can identify both linearly and circularly polarized light [57-60]. Similarly, we chose to study **odonates (damselflies and dragonflies)** because they have physiologically complex eyes [61] and display a diversity of visually-influenced behaviors [62-64]. To study the degeneration of eyes in arthropods from subterranean environments, we examined certain species of **isopods** and **crayfish** in which closely related species or populations live either above or below ground. Specifically, we sequenced tissues from the eye-bearing, surface-dwelling isopod ***Caecidotea forbesi*** and its eyeless, cave-dwelling congeneric ***C. bricrenata***. We also sequenced transcriptomes for different populations of the isopod ***Asellus aquaticus***, which has a surface-dwelling form and multiple cave-dwelling populations with typical cave morphologies like degenerated eyes [65,66]. Likewise, we generated transcriptome data from a pair of surface (***Procambarus alleni****)* and cave (***P. franzi****)* freshwater crayfish. Crayfish have previously been the focus of molecular evolutionary studies of opsin in cave/surface comparisons [67]. To study the evolution of sexually dimorphic eyes, we generated a transcriptome for the RNA expressed by developing eyes from the **ostracod*Euphilomedes carcharodonta***, a species in which males have compound eyes, but females do not [68,69]. Other species in this family of ostracods exhibit a similar, but independently evolved eye dimorphism, suggesting that these ostracods may be a promising system for the study of sex-specific convergent phenotypic evolution [70].

Lastly, we sequenced transcriptomes for ***Tripedalia cystophora***, a **cubozoan cnidarian (box jellyfish)**. Cubozoans are the only cnidarians with camera-type eyes and, for that reason, have been the subject of numerous studies of visual neurobiology [71-74], morphology [75,76], and behavior [77,78]. Transcriptomic resources will aid these efforts. Further, as cnidarians, cubozoans may help us understand the evolutionary origins of the metazoan phototransduction cascade [79-81].

### RNA extraction, cDNA construction, and transcriptome sequencing

We extracted RNA from our tissue samples using either the organic solvent TRIzol (Invitrogen) or the Nucleospin RNA XS kit (Macherey-Nagel), in both cases following manufacturer’s protocol. In cases where we used TRIzol, we removed trace DNA with the Ambion TURBO DNA-free kit (Invitrogen). In all cases, we quantified RNA yield with a Qubit Fluorometer (Invitrogen), following manufacturer’s protocol. To generate cDNA from RNA, we used the SMARTer cDNA synthesis kit (Clontech). To reduce sequencing artifacts due to poly-T tracts, we used modified 3’-primers for first-strand synthesis: 5’-AAG CAG TGG TAT CAA CGC AGA GTA CTTTTTTCTTTTTT-3’. For second strand synthesis, we used the protocol outlined in the SMARTer cDNA kits and a number of cycles determined by a series of optimization procedures. We then purified the amplified cDNA using one volume per sample of phenol:chloroform:isoamyl (25:24:1 v/v/v) and standard protocols. Finally, we sequenced cDNA using the Roche 454 platform. Here, we followed manufacturer’s instructions and employed partial runs with a manifold to separate samples. To assemble our transcriptomes, we used GS De novo Assembler v2.3 (“Newbler”; 454 Life Sciences/Roche Branford, CT USA) set to default threshold options, and using the –vt option to remove adapters. Following assembly, we used LUCY [82,83] to trim low-quality nucleotide reads and delete any assembled contigs below 100 bp in length. Next, we ran isotigs from Newbler through the program iAssembler [84] to combine redundant isotigs, then ran the resulting sequences through the program ‘Get ORFs’ [12,85], ignoring any sequences less than 30 amino acids in length, to produce the predicted protein sequences that we used in our PIA analyses. Assembled sequences and ORFs for our 28 transcriptomes are available on the Bitbucket public repository (http://bitbucket.org/osiris_phylogenetics/pia) and on a publicly-accessible web server (http://galaxy-dev.cnsi.ucsb.edu/pia/).

### Assembling the light-interaction toolkit (LIT)

We assembled the LIT 1.0 by reviewing past research into the molecular components that underlie the function and development of light-interacting structures in metazoans (Additional file 1: Table S1). Specifically, the LIT 1.0 contains molecular components of rhabdomeric- and ciliary- type phototransduction [1,15,16,86], transcription factors involved in the specification and development of photoreceptors and eyes [3,20,87], genes involved in the synthesis and regeneration of the chromophore retinal [46,47,88], lens crystallins [18,19,36,50], reflectins [44], components of the circadian clock pathway [15] , and the enzymes that transport and produce pigments such as melanins [89,90], pterins [91], ommochromes [15,17], and hemes [31]. Genes from LIT 1.0 are an appropriate test case for PIA because the specific functions and expression patterns of many of these genes are well-characterized. Also, certain fundamental aspects of light detection – such as opsin-based phototransduction – appear to involve molecular components that are conserved broadly across metazoan phyla. Thus, we can make well-informed inferences about the functions of new sequences from transcriptomes based on their phylogenetic relatedness to LIT genes that have been characterized previously.

After assembling our list of genes for LIT 1.0, we used functionally characterized exemplars of each of these genes (*i.e.* those from model systems such as fly or mouse; see Additional file 1: Table S1) and the blastp algorithm to search the predicted protein databases associated with 29 fully-sequenced genomes, including those from 24 metazoans, two choanoflagellates, and three fungi (see Additional file 1: Table S1 for search settings and Additional file 4: Table S3 for details on the predicted protein databases that we searched). After removing duplicate genes with Similar Sequence Remover [11], we aligned BLAST hits for each gene using MAFFT [24,25]. We removed genes on long branches using Long Branch Remover [11] and built trees with RAxML assuming WAG as the protein model, and using 100 bootstrap pseudoreplicates followed by maximum likelihood search for the best tree [92,93].

### Phylogenetically-informed annotation (PIA)

We used PIA to search our 28 new transcriptomes for potential orthologs of genes from LIT 1.0. First, we searched translated versions of our transcriptomes using blastp and the same queries that we used to identify sequences for our pre-calculated gene trees (Additional file 1: Table S1). We used stringent settings for blastp, specifically taking the top three hits that surpassed an e-value cut-off of 1e-20. Future users of PIA have the option of retaining different numbers of top hits and adjusting the e-value cut-off point as they see fit. Next, we used MAFFT to align the hits from our BLAST searches against the sequences that we used to calculate trees for our 109 LIT genes. PIA provides the option of aligning sequences using MUSCLE [23], MAFFT [24,25], or MAFFT-profile [94]. MAFFT-profile is the fastest of the three options because it does not re-align all sequences, but rather aligns the new sequences to an existing alignment. Finally, PIA uses an Evolutionary Placement Algorithm (implemented in RAxML; [26,27]) to place the potential LIT genes on to our pre-calculated gene trees using Maximum Likelihood. Briefly, EPA places new genes on each branch of a pre-calculated phylogeny and calculates a likelihood score. The placement with the best score is retained. This is much faster than recalculating the entire gene tree.

## Availability and requirements

**Project Name**: Phylogenetically-Informed Annotation (PIA).

**Project Home Page**: http://galaxy-dev.cnsi.ucsb.edu/pia/.

**Project Demonstration Page**: http://galaxy-dev.cnsi.ucsb.edu/pia/.

**Operating System**: Any Internet Browser.

**Programming Language**: Python, Perl, C, Java, and others.

**Other Requirements**: For a local instance, install Galaxy (http://galaxyproject.org) and required tools.

**License**: All original source code for PIA is available under the MIT license (http://opensource.org/licenses/mit-license.html). See below:

The MIT License (MIT).

Copyright (c) 2014 Speiser et al.

Permission is hereby granted, free of charge, to any person obtaining a copy of this software and associated documentation files (the "Software"), to deal in the Software without restriction, including without limitation the rights to use, copy, modify, merge, publish, distribute, sublicense, and/or sell copies of the Software, and to permit persons to whom the Software is furnished to do so, subject to the following conditions: The above copyright notice and this permission notice shall be included in all copies or substantial portions of the Software.

THE SOFTWARE IS PROVIDED "AS IS", WITHOUT WARRANTY OF ANY KIND, EXPRESS OR IMPLIED, INCLUDING BUT NOT LIMITED TO THE WARRANTIES OF MERCHANTABILITY, FITNESS FOR A PARTICULAR PURPOSE AND NONINFRINGEMENT. IN NO EVENT SHALL THE AUTHORS OR COPYRIGHT HOLDERS BE LIABLE FOR ANY CLAIM, DAMAGES OR OTHER LIABILITY, WHETHER IN AN ACTION OF CONTRACT, TORT OR OTHERWISE, ARISING

FROM, OUT OF OR IN CONNECTION WITH THE SOFTWARE OR THE USE OR OTHER DEALINGS IN THE SOFTWARE.

**Restrictions**: None.

## Additional files

Additional file 1: Table S1. Genes from a metazoan Light Interaction Toolkit (LIT 1.0). For each gene in the table, we provide the following: a full name; abbreviations for orthologs (or paralogs) of the gene from the model systems *Drosophila melanogaster* (Dmel) and *Mus musculus* (Mmus); the gene set under which each gene can be found on our public website for Phylogenetically-Informed Annotation (PIA); a citation for a paper discussing the gene (see References in the main text); and, finally, the e-value cut-offs and the queries (identified by NCBI accession numbers) that we used to search for relatives of each gene when building our trees. Additional file 2:
**LIT PIA trees.**
Additional file 3: Table S2. Statistics for the transcriptomes that we generated using the Roche 454 platform. We generated these statistics using the tool assemblystats (version 1.0.1) available in Galaxy. In the table, the numbered columns represent the following transcriptomes: 1 = *Chiroteuthis calyx* eye; 2 = *Euprymna scolopes* eye; 3 = *Galiteuthis armata* eye; 4 = *Octopus bimaculoides* skin; 5 = *Uroteuthis edulis* eye; 6 = *Vampyroteuthis infernalis* eye; 7 = *Asellus aquaticus* cave head; 8 = *Asellus aquaticus* embryos and hatchlings; 9 = *Asellus aquaticus* hybrid head; 10 = *Asellus aquaticus* surface head; 11 = *Benthesicymus bartletti* eye; 12 = *Caecidotea bicrenata* adult head; 13 = *Caecidotea bicrenata* embryos; 14 = *Caecidotea forbesi* adult head; 15 = *Caecidotea forbesi* embryos; 16 = *Euphilomedes carcharodonta* embryos; 17 = *Hemisquilla californiensis* eyes; 18 = *Ischnura ramburii* head; 19 = *Limulus polyphemus* lateral eye; 20 = *Limulus polyphemus* median eye; 21 = *Limulus polyphemus* ventral eye; 22 = *Procambarus alleni* eye; 23 = *Procambarus franzi* eye; 24 = *Pseudosquilla* ciliata eye; 25 = *Systellaspis debilis* eye; 26 = *Telebasis salva* head; 27 = *Tripedalia cystophora* eyes; 28 = *Tripedalia cystophora* planula larvae. Additional file 4: Table S3. The fully sequenced genomes that we searched for relatives of genes from LIT 1.0 when building our gene trees. For each genome, we provide the following: the species name; the group responsible for generating the genome; the version of the genome that we searched; and a citation for a paper that describes the genome (see Supplementary References). Additional file 5:
**Supplementary References for Table S3.**
