# Supplementary material for: Using phylogenetically-informed annotation (PIA) to search for light-interacting genes in transcriptomes from non-model organisms
Source: BMC Bioinformatics. 2014 Nov 19;15(1):350. doi: 10.1186/s12859-014-0350-x (PMC4255452; doi:10.1186/s12859-014-0350-x)

Tree File: eya

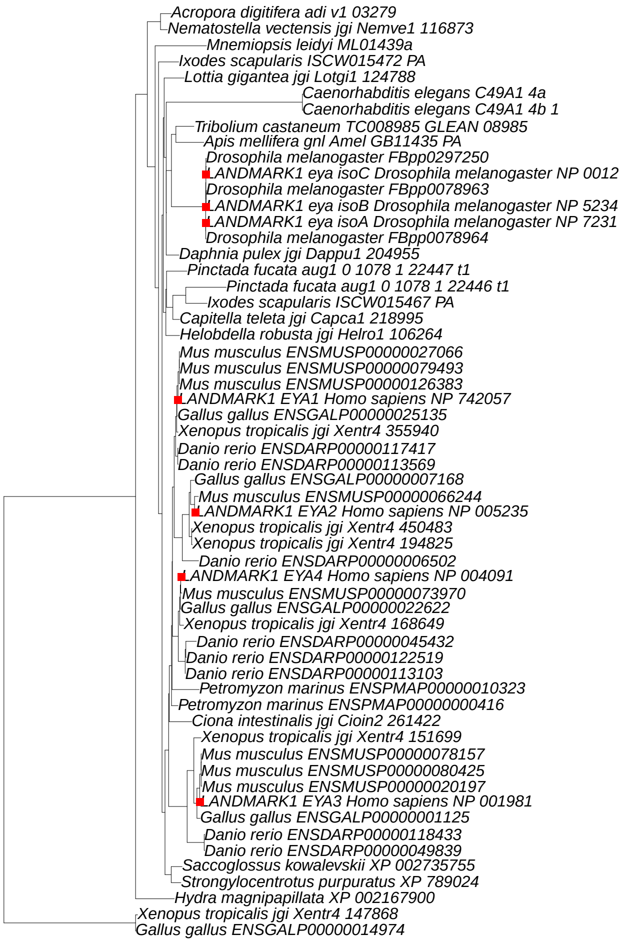

Tree File: six4

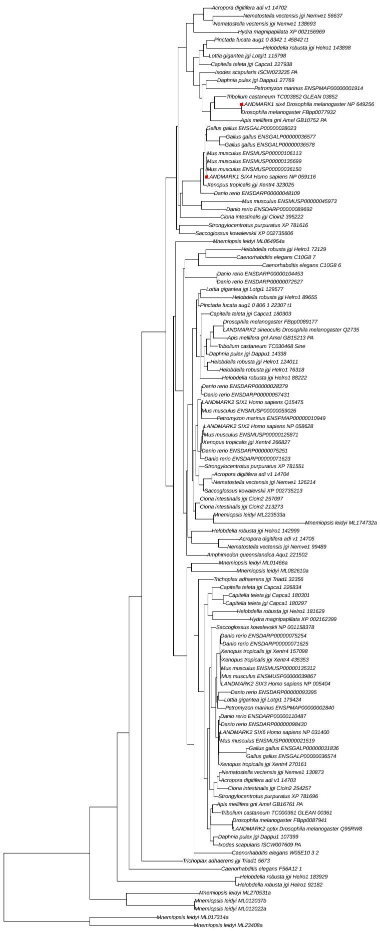

Tree File: ey_toy

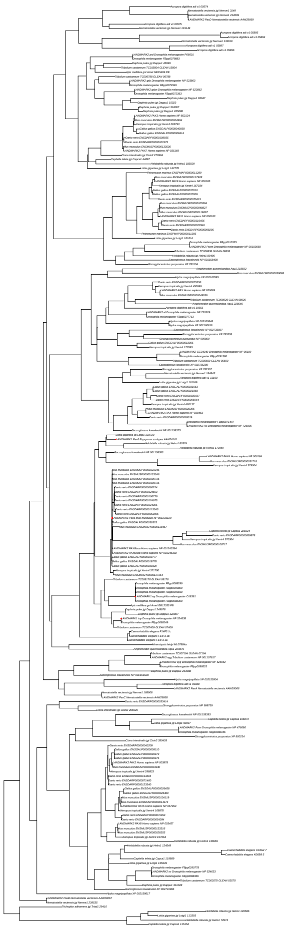

Tree File: ovo

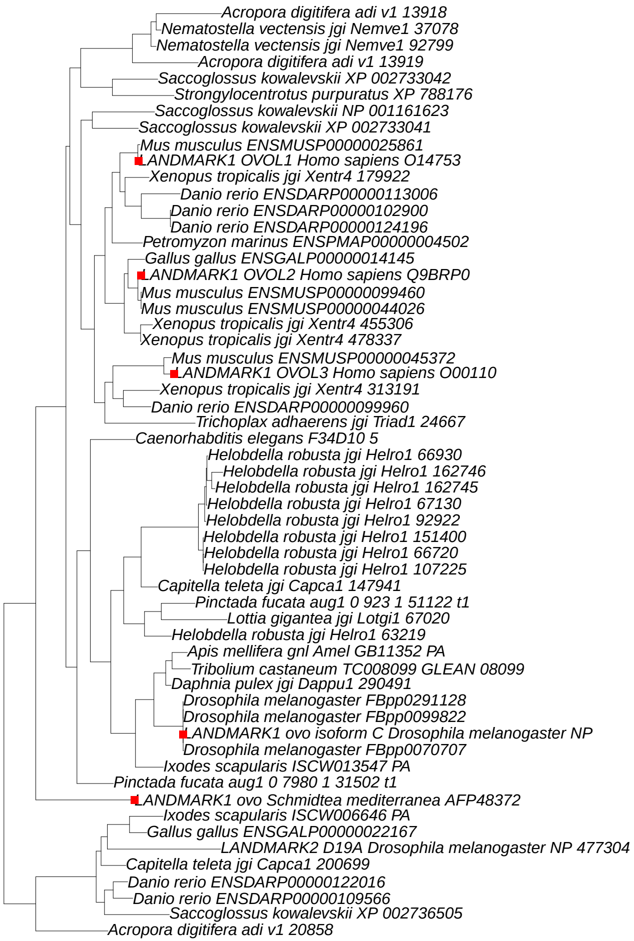

Tree File: optix

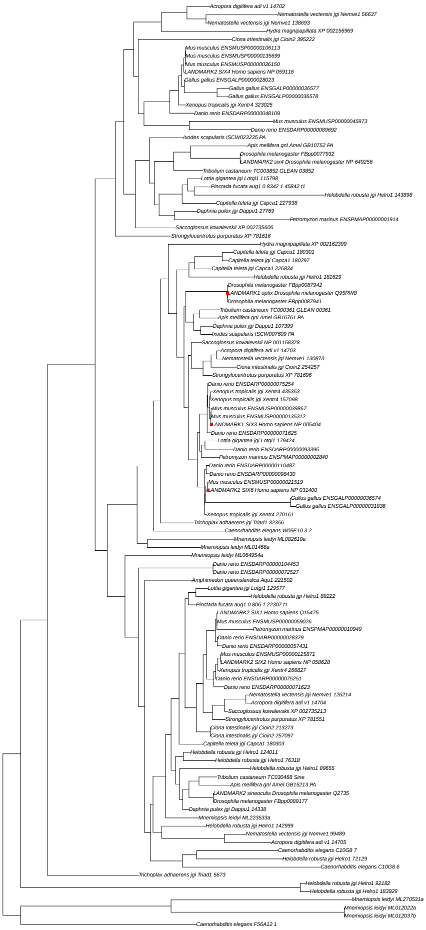

Tree File: so

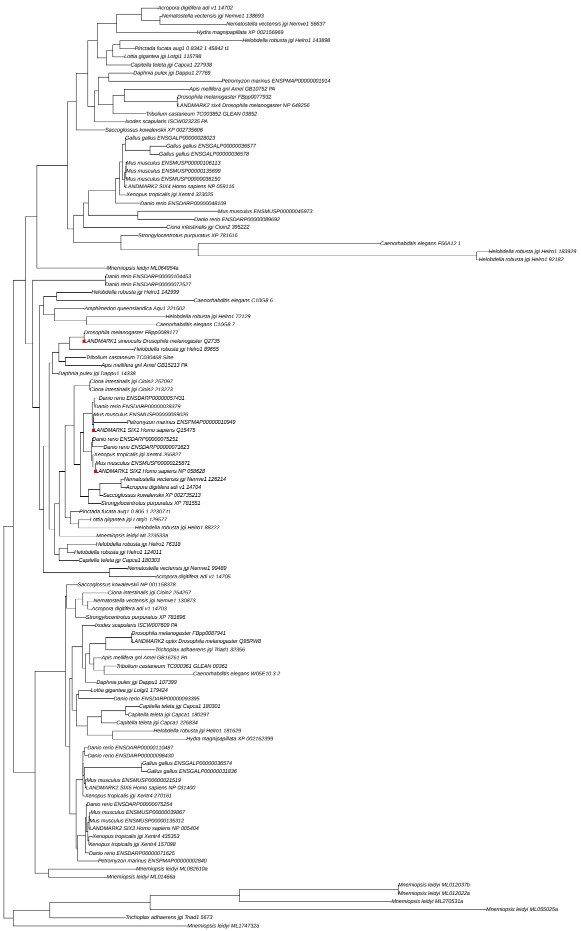

Acropora digitifera adi v1 14702
Nematostella vectensis jgi Nemve1 138693
Nematostella vectensis jgi Nemve1 56637
Hydra magnipapillata XP 002156969
Helobdella robusta jgi Helro1 143898
Pinctada fucata aug1 0 8342 1 45842 t1
Lottia gigantea jgi Lotgi1 115798
Capitella teleta jgi Capca1 227938
Daphnia pulex jgi Dappu1 27769
Petromyzon marinus ENSPMAP00000001914
Apis mellifera gnl Amel GB10752 PA
Drosophila melanogaster FBpp0077932
LANDMARK2 six4 Drosophila melanogaster NP 649256
Tribolium castaneum TC003852 GLEAN 03852
Ixodes scapularis ISCW023235 PA
Saccoglossus kowalevskii XP 002735606
Gallus gallus ENSGALP00000028023
Gallus gallus ENSGALP00000036577
Gallus gallus ENSGALP00000036578
Mus musculus ENSMUSP00000106113
Mus musculus ENSMUSP00000135699
Mus musculus ENSMUSP00000036150
LANDMARK2 SIX4 Homo sapiens NP 059116
Xenopus tropicalis jgi Xentr4 323025
Danio rerio ENSDARP00000048109
Mus musculus ENSMUSP00000045973
Danio rerio ENSDARP00000089692
Ciona intestinalis jgi Cioin2 395222
Strongylocentrotus purpuratus XP 781616
Caenorhabditis elegans F56A12 1
Helobdella robusta jgi Helro1 183929
Helobdella robusta jgi Helro1 92182
Mnemiopsis leidyi ML064954a
Danio rerio ENSDARP00000104453
Danio rerio ENSDARP00000072527
Helobdella robusta jgi Helro1 142999
Caenorhabditis elegans C10G8 6
Amphimedon queenslandica Aqu1 221502
Helobdella robusta jgi Helro1 72129
Caenorhabditis elegans C10G8 7
Drosophila melanogaster FBpp0089177
LANDMARK1 sineoculis Drosophila melanogaster Q2735
Helobdella robusta jgi Helro1 89655
Tribolium castaneum TC030468 Sine
Apis mellifera gnl Amel GB15213 PA
Daphnia pulex jgi Dappu1 14338
Ciona intestinalis jgi Cioin2 257097
Ciona intestinalis jgi Cioin2 213273
Danio rerio ENSDARP00000057431
Danio rerio ENSDARP00000028379
Mus musculus ENSMUSP00000059026
Petromyzon marinus ENSPMAP00000010949
LANDMARK1 SIX1 Homo sapiens Q15475
Danio rerio ENSDARP00000075251
Danio rerio ENSDARP00000071623
Xenopus tropicalis jgi Xentr4 266827
Mus musculus ENSMUSP00000125871
LANDMARK1 SIX2 Homo sapiens NP 058628
Nematostella vectensis jgi Nemve1 126214
Acropora digitifera adi v1 14704
Saccoglossus kowalevskii XP 002735213
Strongylocentrotus purpuratus XP 781551
Pinctada fucata aug1 0 806 1 22307 t1
Lottia gigantea jgi Lotgi1 129577
Helobdella robusta jgi Helro1 88222
Mnemiopsis leidyi ML223533a
Helobdella robusta jgi Helro1 76318
Helobdella robusta jgi Helro1 124011
Capitella teleta jgi Capca1 180303
Nematostella vectensis jgi Nemve1 99489
Acropora digitifera adi v1 14705
Saccoglossus kowalevskii NP 001158378
Ciona intestinalis jgi Cioin2 254257
Nematostella vectensis jgi Nemve1 130873
Acropora digitifera adi v1 14703
Strongylocentrotus purpuratus XP 781696
Ixodes scapularis ISCW007609 PA
Drosophila melanogaster FBpp0087941
LANDMARK2 optix Drosophila melanogaster Q95RW8
Trichoplax adhaerens jgi Triad1 32356
Apis mellifera gnl Amel GB16761 PA
Tribolium castaneum TC000361 GLEAN 00361
Caenorhabditis elegans W05E10 3 2
Daphnia pulex jgi Dappu1 107399
Lottia gigantea jgi Lotgi1 179424
Danio rerio ENSDARP00000093395
Capitella teleta jgi Capca1 180301
Capitella teleta jgi Capca1 180297
Capitella teleta jgi Capca1 226834
Helobdella robusta jgi Helro1 181629
Hydra magnipapillata XP 002162399
Danio rerio ENSDARP00000110487
Danio rerio ENSDARP00000098430
Gallus gallus ENSGALP00000036574
Gallus gallus ENSGALP00000031836
Mus musculus ENSMUSP00000021519
LANDMARK2 SIX6 Homo sapiens NP 031400
Xenopus tropicalis jgi Xentr4 270161
Danio rerio ENSDARP00000075254
Mus musculus ENSMUSP00000039867
Mus musculus ENSMUSP00000135312
LANDMARK2 SIX3 Homo sapiens NP 005404
Xenopus tropicalis jgi Xentr4 435353
Xenopus tropicalis jgi Xentr4 157098
Danio rerio ENSDARP00000071625
Petromyzon marinus ENSPMAP00000002840
Mnemiopsis leidyi ML082610a
Mnemiopsis leidyi ML01466a
Mnemiopsis leidyi ML012037b
Mnemiopsis leidyi ML012022a
Mnemiopsis leidyi ML270531a
Mnemiopsis leidyi ML055025a
Trichoplax adhaerens jgi Triad1 5673
Mnemiopsis leidyi ML174732a

Tree File: rx

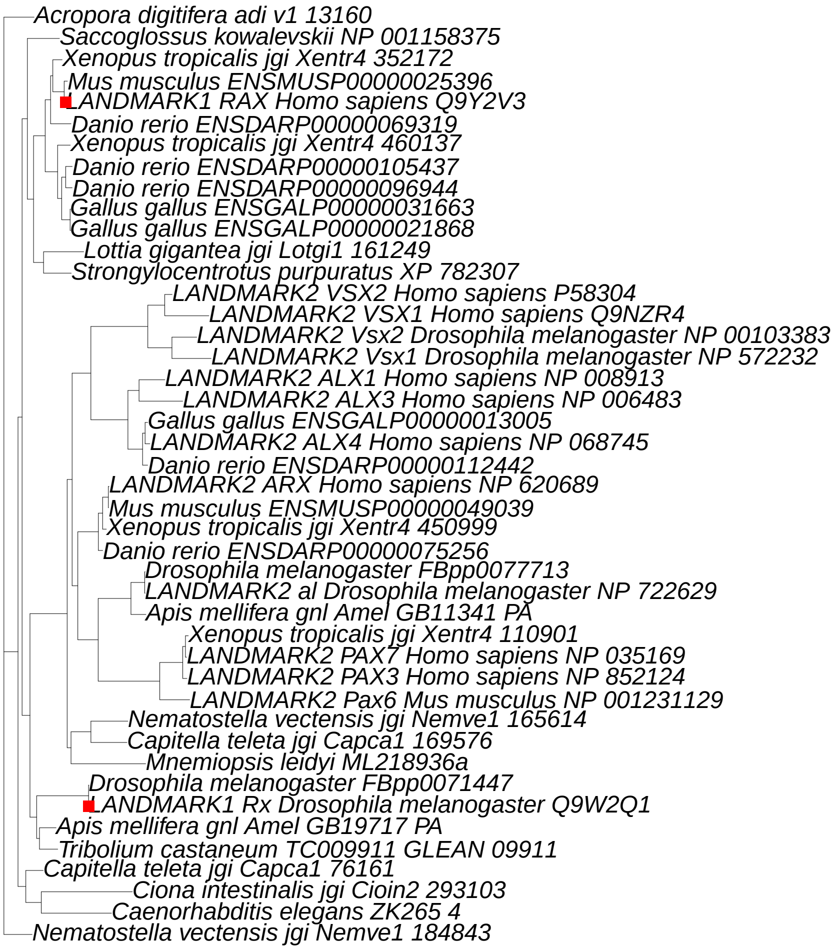

Acropora digitifera adi v1 13160
Saccoglossus kowalevskii NP 001158375
Xenopus tropicalis jgi Xentr4 352172
Mus musculus ENSMUSP00000025396
LANDMARK1 RAX Homo sapiens Q9Y2V3
Danio rerio ENSDARP00000069319
Xenopus tropicalis jgi Xentr4 460137
Danio rerio ENSDARP00000105437
Danio rerio ENSDARP00000096944
Gallus gallus ENSGALP00000031663
Gallus gallus ENSGALP00000021868
Lottia gigantea jgi Lotgi1 161249
Strongylocentrotus purpuratus XP 782307
LANDMARK2 VSX2 Homo sapiens P58304
LANDMARK2 VSX1 Homo sapiens Q9NZR4
LANDMARK2 Vsx2 Drosophila melanogaster NP 00103383
LANDMARK2 Vsx1 Drosophila melanogaster NP 572232
LANDMARK2 ALX1 Homo sapiens NP 008913
LANDMARK2 ALX3 Homo sapiens NP 006483
Gallus gallus ENSGALP00000013005
LANDMARK2 ALX4 Homo sapiens NP 068745
Danio rerio ENSDARP00000112442
LANDMARK2 ARX Homo sapiens NP 620689
Mus musculus ENSMUSP00000049039
Xenopus tropicalis jgi Xentr4 450999
Danio rerio ENSDARP00000075256
Drosophila melanogaster FBpp0077713
LANDMARK2 al Drosophila melanogaster NP 722629
Apis mellifera gnl Amel GB11341 PA
Xenopus tropicalis jgi Xentr4 110901
LANDMARK2 PAX7 Homo sapiens NP 035169
LANDMARK2 PAX3 Homo sapiens NP 852124
LANDMARK2 Pax6 Mus musculus NP 001231129
Nematostella vectensis jgi Nemve1 165614
Capitella teleta jgi Capca1 169576
Mnemiopsis leidyi ML218936a
Drosophila melanogaster FBpp0071447
LANDMARK1 Rx Drosophila melanogaster Q9W2Q1
Apis mellifera gnl Amel GB19717 PA
Tribolium castaneum TC009911 GLEAN 09911
Capitella teleta jgi Capca1 76161
Ciona intestinalis jgi Cioin2 293103
Caenorhabditis elegans ZK265 4
Nematostella vectensis jgi Nemve1 184843

Tree File: dac

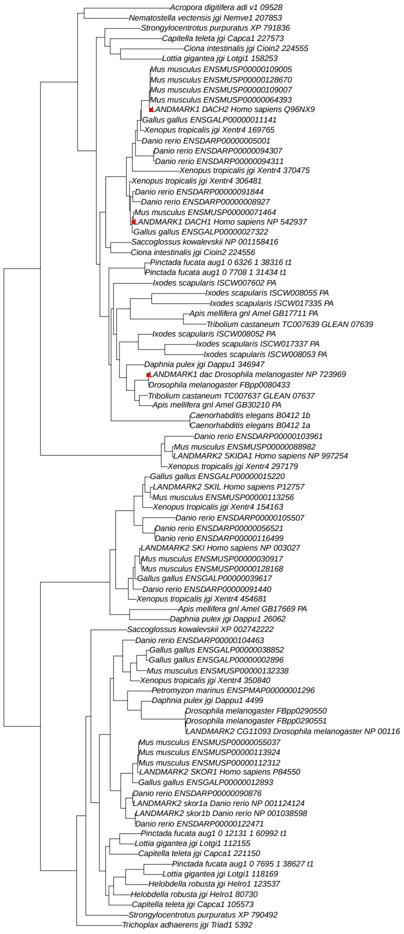

Tree File: vsx

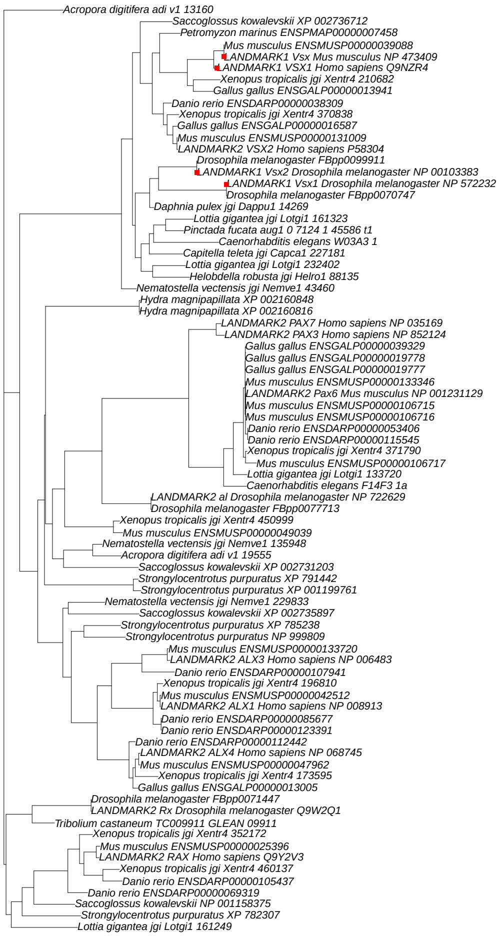

Tree File: oc

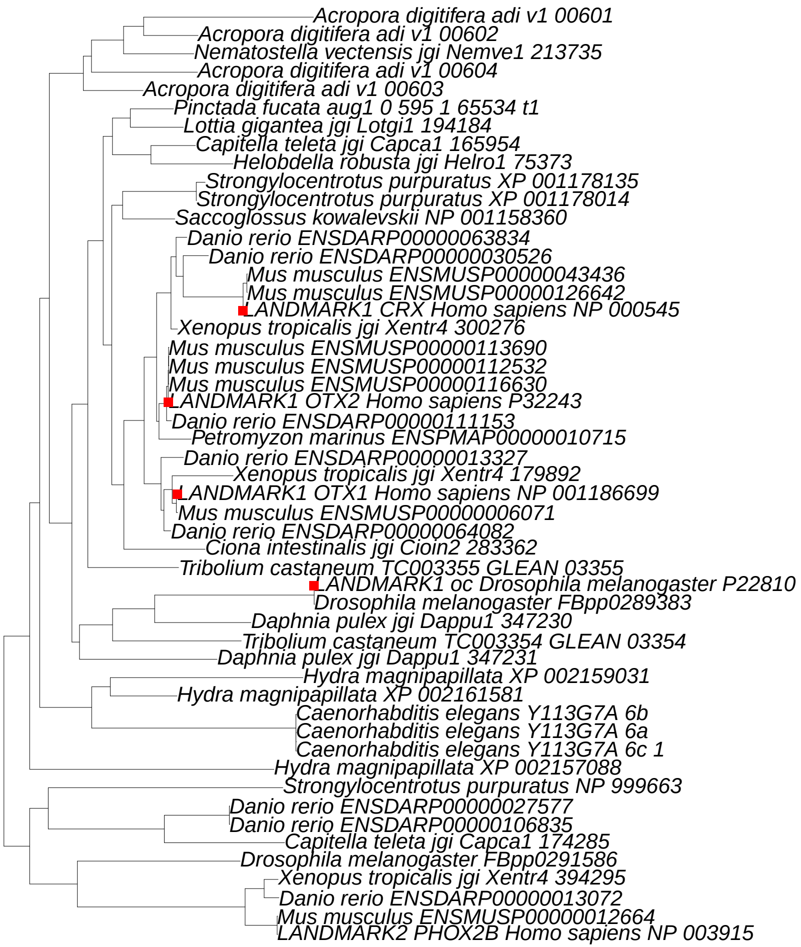
Acropora digitifera adi v1 00601
Acropora digitifera adi v1 00602
Nematostella vectensis jgi Nemve1 213735
Acropora digitifera adi v1 00604
Acropora digitifera adi v1 00603
Pinctada fucata aug1 0 595 1 65534 t1
Lottia gigantea jgi Lotgi1 194184
Capitella teleta jgi Capca1 165954
Helobdella robusta jgi Helro1 75373
Strongylocentrotus purpuratus XP 001178135
Strongylocentrotus purpuratus XP 001178014
Saccoglossus kowalevskii NP 001158360
Danio rerio ENSDARP00000063834
Danio rerio ENSDARP00000030526
Mus musculus ENSMUSP00000043436
Mus musculus ENSMUSP00000126642
LANDMARK1 CRX Homo sapiens NP 000545
Xenopus tropicalis jgi Xentr4 300276
Mus musculus ENSMUSP00000113690
Mus musculus ENSMUSP00000112532
Mus musculus ENSMUSP00000116630
LANDMARK1 OTX2 Homo sapiens P32243
Danio rerio ENSDARP00000111153
Petromyzon marinus ENSPMAP00000010715
Danio rerio ENSDARP00000013327
Xenopus tropicalis jgi Xentr4 179892
LANDMARK1 OTX1 Homo sapiens NP 001186699
Mus musculus ENSMUSP00000006071
Danio rerio ENSDARP00000064082
Ciona intestinalis jgi Cioin2 283362
Tribolium castaneum TC003355 GLEAN 03355
LANDMARK1 oc Drosophila melanogaster P22810
Drosophila melanogaster FBpp0289383
Daphnia pulex jgi Dappu1 347230
Tribolium castaneum TC003354 GLEAN 03354
Daphnia pulex jgi Dappu1 347231
Hydra magnipapillata XP 002159031
Hydra magnipapillata XP 002161581
Caenorhabditis elegans Y113G7A 6b
Caenorhabditis elegans Y113G7A 6a
Caenorhabditis elegans Y113G7A 6c 1
Hydra magnipapillata XP 002157088
Strongylocentrotus purpuratus NP 999663
Danio rerio ENSDARP00000027577
Danio rerio ENSDARP00000106835
Capitella teleta jgi Capca1 174285
Drosophila melanogaster FBpp0291586
Xenopus tropicalis jgi Xentr4 394295
Danio rerio ENSDARP00000013072
Mus musculus ENSMUSP00000012664
LANDMARK2 PHOX2B Homo sapiens NP 003915

Tree File: glass

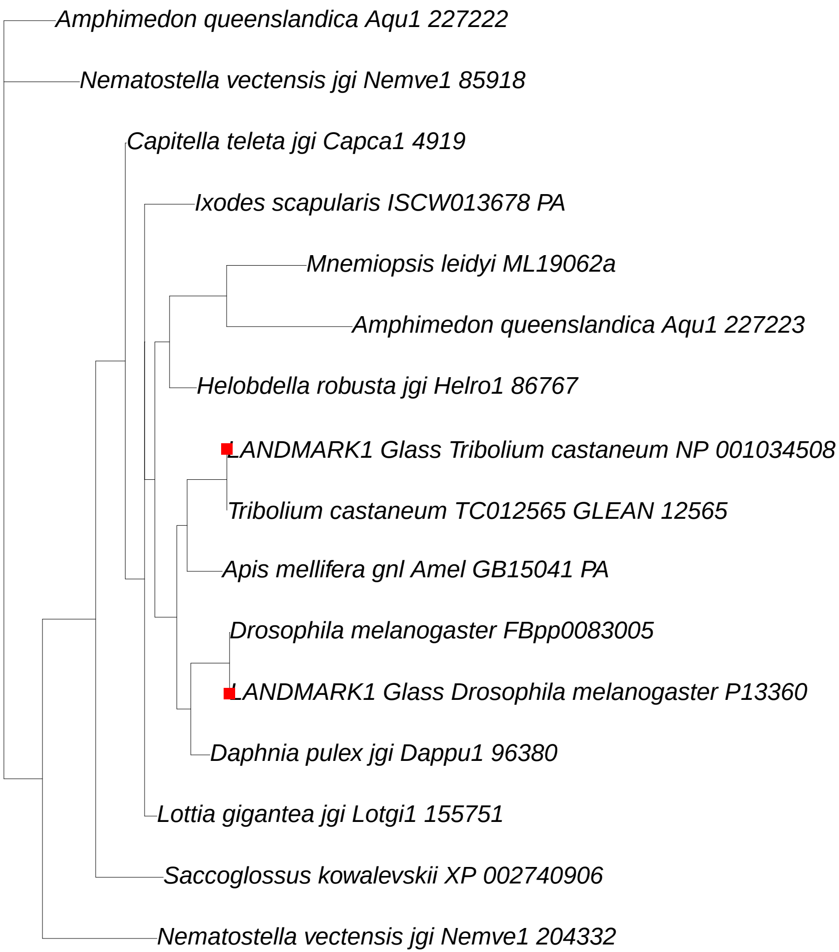

Tree File: pph13

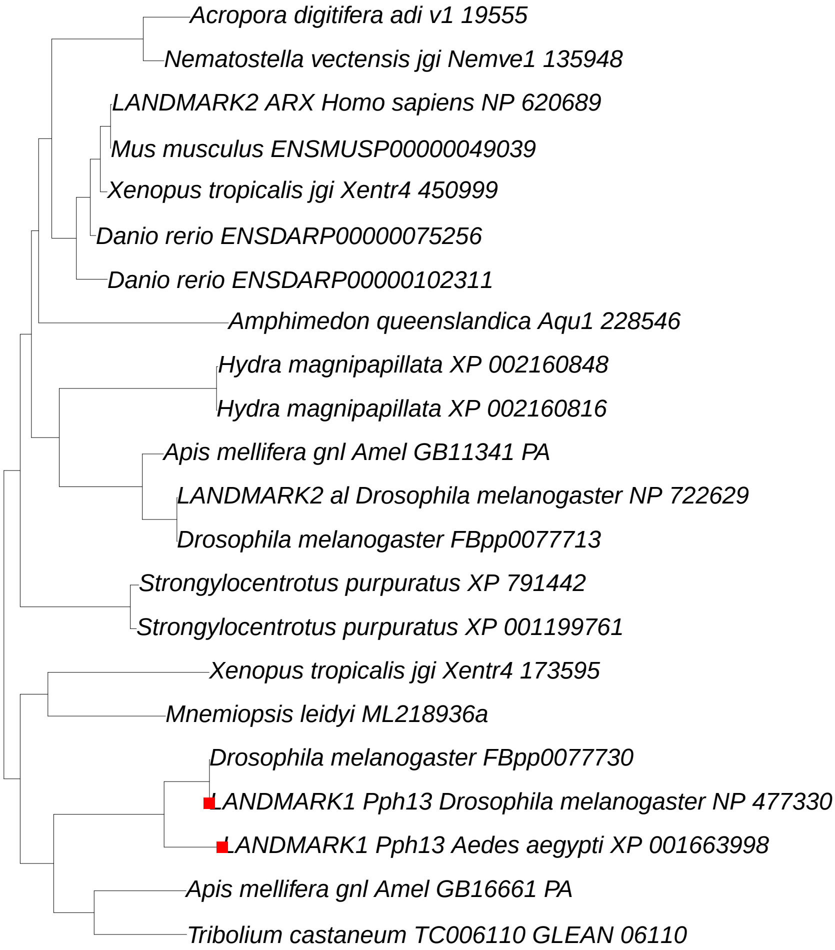

Tree File: notch

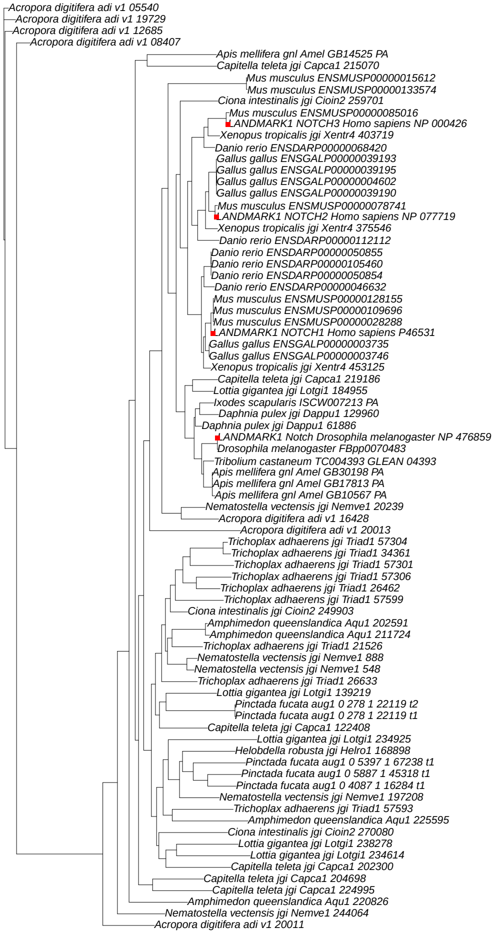

Tree File: egfr

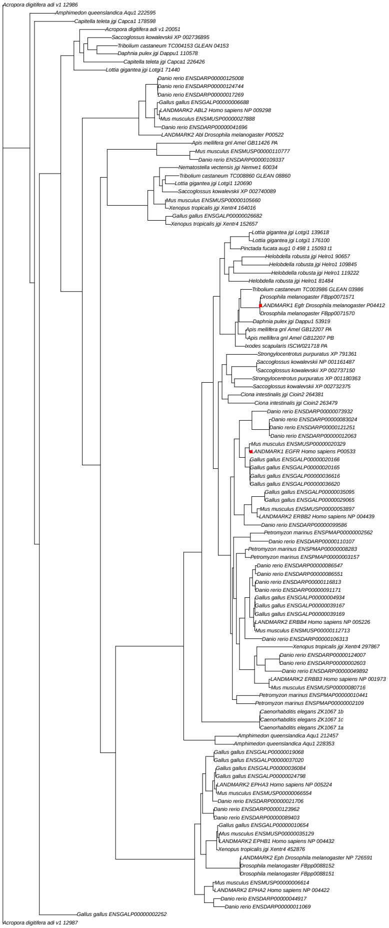

Tree File: en

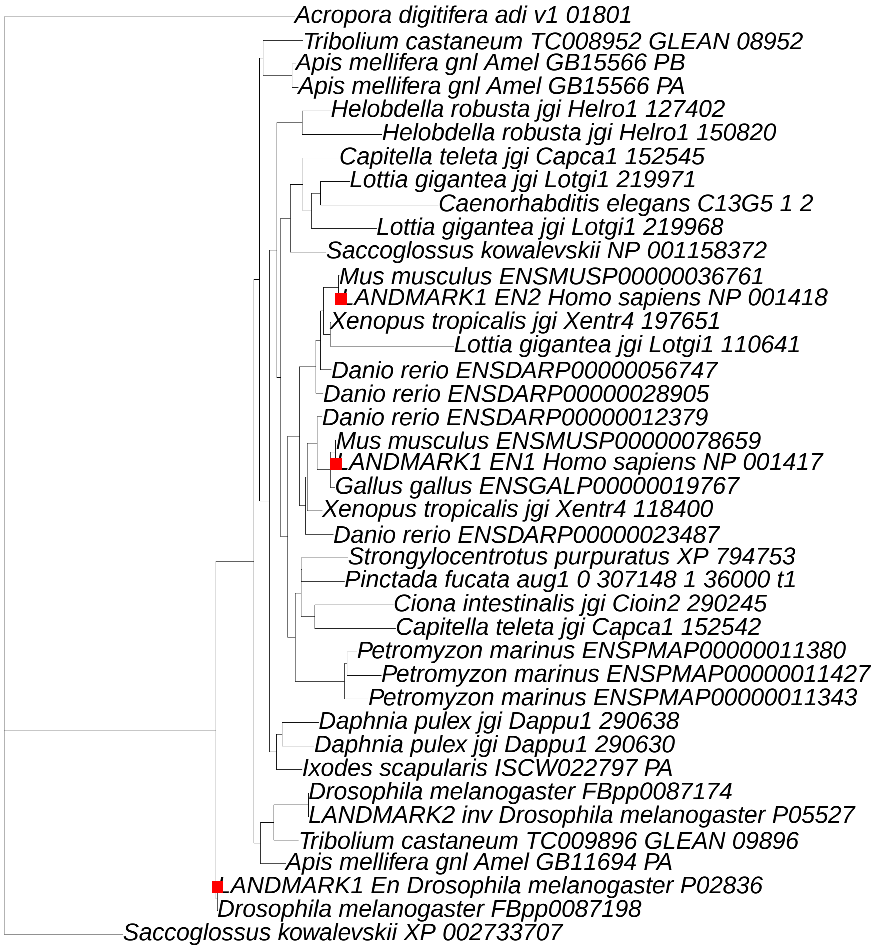
Acropora digitifera adi v1 01801
Tribolium castaneum TC008952 GLEAN 08952
Apis mellifera gnl Amel GB15566 PB
Apis mellifera gnl Amel GB15566 PA
Helobdella robusta jgi Helro1 127402
Helobdella robusta jgi Helro1 150820
Capitella teleta jgi Capca1 152545
Lottia gigantea jgi Lotgi1 219971
Caenorhabditis elegans C13G5 1 2
Lottia gigantea jgi Lotgi1 219968
Saccoglossus kowalevskii NP 001158372
Mus musculus ENSMUSP00000036761
LANDMARK1 EN2 Homo sapiens NP 001418
Xenopus tropicalis jgi Xentr4 197651
Lottia gigantea jgi Lotgi1 110641
Danio rerio ENSDARP00000056747
Danio rerio ENSDARP00000028905
Danio rerio ENSDARP00000012379
Mus musculus ENSMUSP00000078659
LANDMARK1 EN1 Homo sapiens NP 001417
Gallus gallus ENSGALP00000019767
Xenopus tropicalis jgi Xentr4 118400
Danio rerio ENSDARP00000023487
Strongylocentrotus purpuratus XP 794753
Pinctada fucata aug1 0 307148 1 36000 t1
Ciona intestinalis jgi Cioin2 290245
Capitella teleta jgi Capca1 152542
Petromyzon marinus ENSPMAP00000011380
Petromyzon marinus ENSPMAP00000011427
Petromyzon marinus ENSPMAP00000011343
Daphnia pulex jgi Dappu1 290638
Daphnia pulex jgi Dappu1 290630
Ixodes scapularis ISCW022797 PA
Drosophila melanogaster FBpp0087174
LANDMARK2 inv Drosophila melanogaster P05527
Tribolium castaneum TC009896 GLEAN 09896
Apis mellifera gnl Amel GB11694 PA
LANDMARK1 En Drosophila melanogaster P02836
Drosophila melanogaster FBpp0087198
Saccoglossus kowalevskii XP 002733707

Tree File: dpp

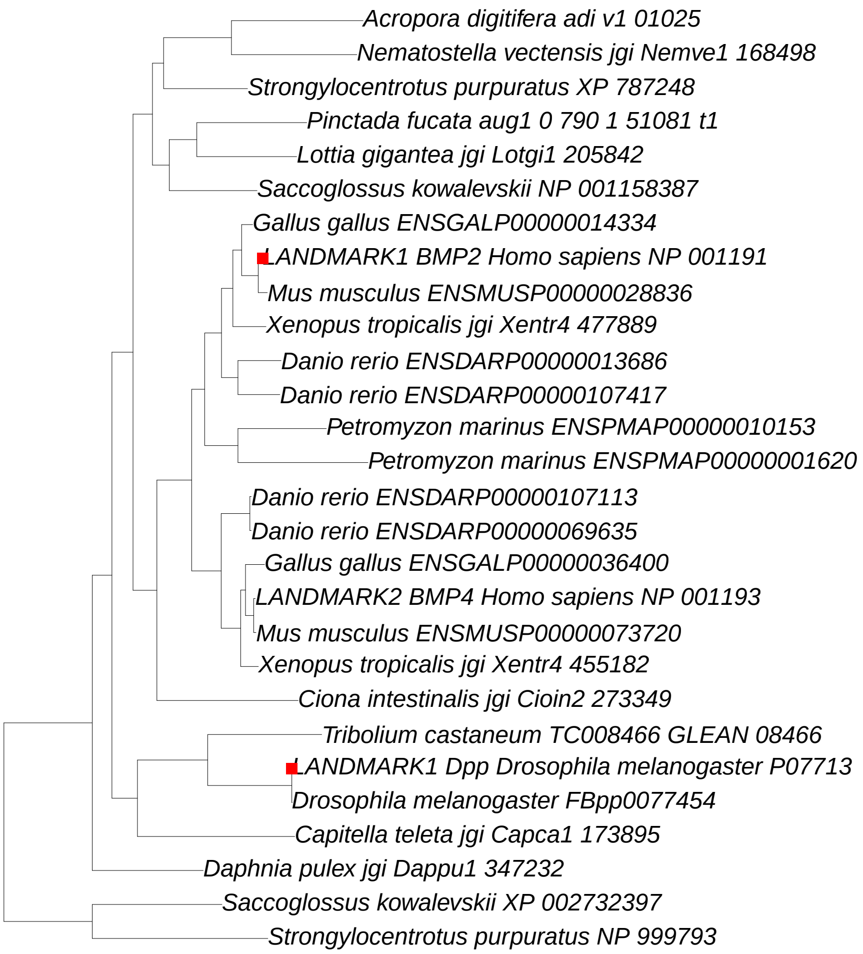

Tree File: wnt1

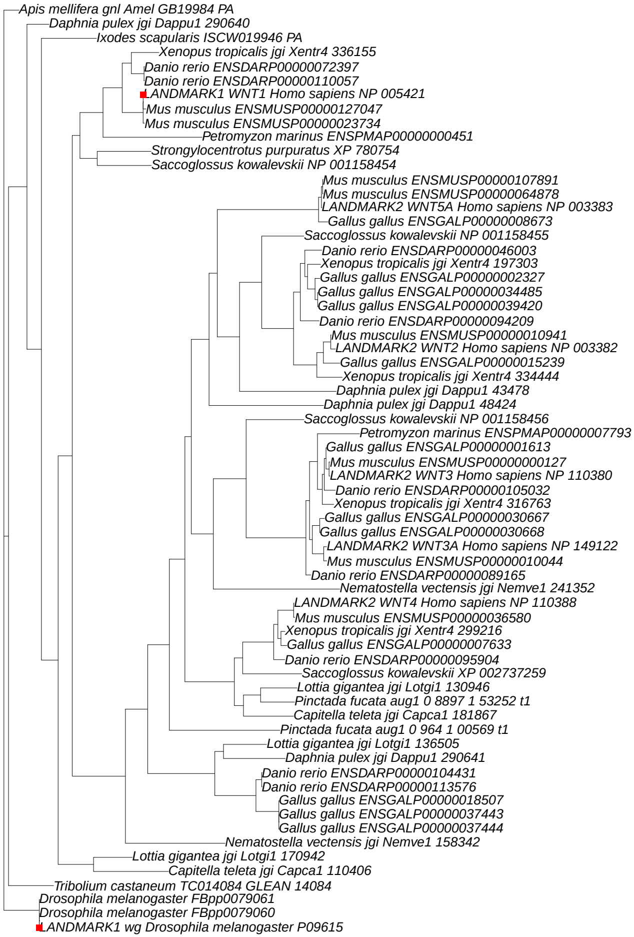

Tree File: hh

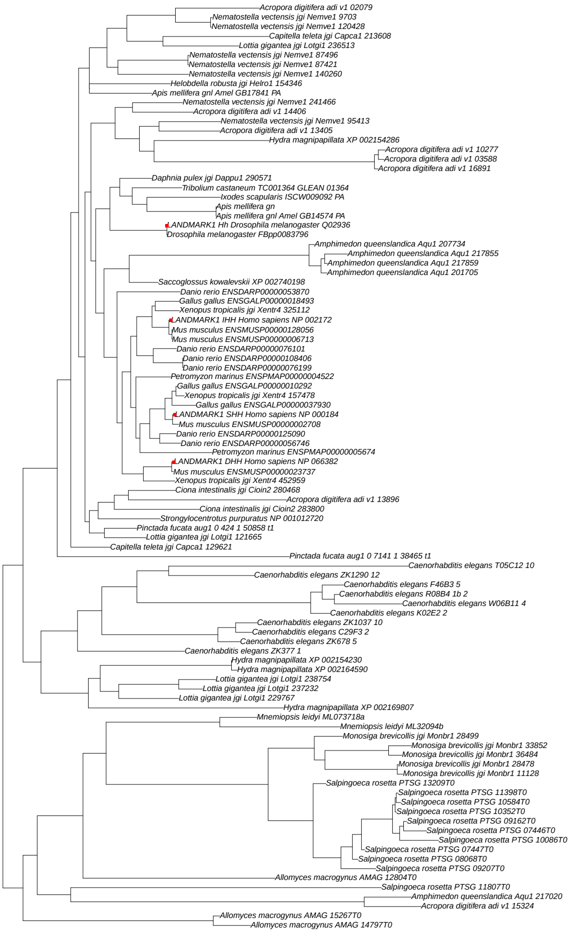

Acropora digitifera adi v1 02079
Nematostella vectensis jgi Nemve1 9703
Nematostella vectensis jgi Nemve1 120428
Capitella teleta jgi Capca1 213608
Lottia gigantea jgi Lotgi1 236513
Nematostella vectensis jgi Nemve1 87496
Nematostella vectensis jgi Nemve1 87421
Nematostella vectensis jgi Nemve1 140260
Helobdella robusta jgi Helro1 154346
Apis mellifera gnl Amel GB17841 PA
Nematostella vectensis jgi Nemve1 241466
Acropora digitifera adi v1 14406
Nematostella vectensis jgi Nemve1 95413
Acropora digitifera adi v1 13405
Hydra magnipapillata XP 002154286
Acropora digitifera adi v1 10277
Acropora digitifera adi v1 03588
Acropora digitifera adi v1 16891
Daphnia pulex jgi Dappu1 290571
Tribolium castaneum TC001364 GLEAN 01364
Ixodes scapularis ISCW009092 PA
Apis mellifera gn
Apis mellifera gnl Amel GB14574 PA
LANDMARK1 Hh Drosophila melanogaster Q02936
Drosophila melanogaster FBpp0083796
Amphimedon queenslandica Aqu1 207734
Amphimedon queenslandica Aqu1 217855
Amphimedon queenslandica Aqu1 217859
Amphimedon queenslandica Aqu1 201705
Saccoglossus kowalevskii XP 002740198
Danio rerio ENSDARP00000053870
Gallus gallus ENSGALP00000018493
Xenopus tropicalis jgi Xentr4 325112
LANDMARK1 IHH Homo sapiens NP 002172
Mus musculus ENSMUSP00000128056
Mus musculus ENSMUSP00000006713
Danio rerio ENSDARP00000076101
Danio rerio ENSDARP00000108406
Danio rerio ENSDARP00000076199
Petromyzon marinus ENSPMAP00000004522
Gallus gallus ENSGALP00000010292
Xenopus tropicalis jgi Xentr4 157478
Gallus gallus ENSGALP00000037930
LANDMARK1 SHH Homo sapiens NP 000184
Mus musculus ENSMUSP00000002708
Danio rerio ENSDARP00000125090
Danio rerio ENSDARP00000056746
Petromyzon marinus ENSPMAP00000005674
LANDMARK1 DHH Homo sapiens NP 066382
Mus musculus ENSMUSP00000023737
Xenopus tropicalis jgi Xentr4 452959
Ciona intestinalis jgi Cioin2 280468
Acropora digitifera adi v1 13896
Ciona intestinalis jgi Cioin2 283800
Strongylocentrotus purpuratus NP 001012720
Pinctada fucata aug1 0 424 1 50858 t1
Lottia gigantea jgi Lotgi1 121665
Capitella teleta jgi Capca1 129621
Pinctada fucata aug1 0 7141 1 38465 t1
Caenorhabditis elegans T05C12 10
Caenorhabditis elegans ZK1290 12
Caenorhabditis elegans F46B3 5
Caenorhabditis elegans R08B4 1b 2
Caenorhabditis elegans W06B11 4
Caenorhabditis elegans K02E2 2
Caenorhabditis elegans ZK1037 10
Caenorhabditis elegans C29F3 2
Caenorhabditis elegans ZK678 5
Caenorhabditis elegans ZK377 1
Hydra magnipapillata XP 002154230
Hydra magnipapillata XP 002164590
Lottia gigantea jgi Lotgi1 238754
Lottia gigantea jgi Lotgi1 237232
Lottia gigantea jgi Lotgi1 229767
Hydra magnipapillata XP 002169807
Mnemiopsis leidyi ML073718a
Mnemiopsis leidyi ML32094b
Monosiga brevicollis jgi Monbr1 28499
Monosiga brevicollis jgi Monbr1 33852
Monosiga brevicollis jgi Monbr1 36484
Monosiga brevicollis jgi Monbr1 28478
Monosiga brevicollis jgi Monbr1 11128
Salpingoeca rosetta PTSG 13209T0
Salpingoeca rosetta PTSG 11398T0
Salpingoeca rosetta PTSG 10584T0
Salpingoeca rosetta PTSG 10352T0
Salpingoeca rosetta PTSG 09162T0
Salpingoeca rosetta PTSG 07446T0
Salpingoeca rosetta PTSG 10086T0
Salpingoeca rosetta PTSG 07447T0
Salpingoeca rosetta PTSG 08068T0
Salpingoeca rosetta PTSG 09207T0
Allomyces macrogynus AMAG 12804T0
Salpingoeca rosetta PTSG 11807T0
Amphimedon queenslandica Aqu1 217020
Acropora digitifera adi v1 15324
Allomyces macrogynus AMAG 15267T0
Allomyces macrogynus AMAG 14797T0

Tree File: r_opsin

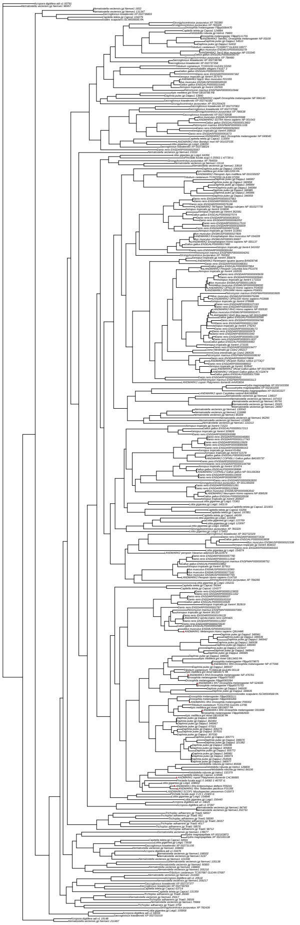

Tree File: trp

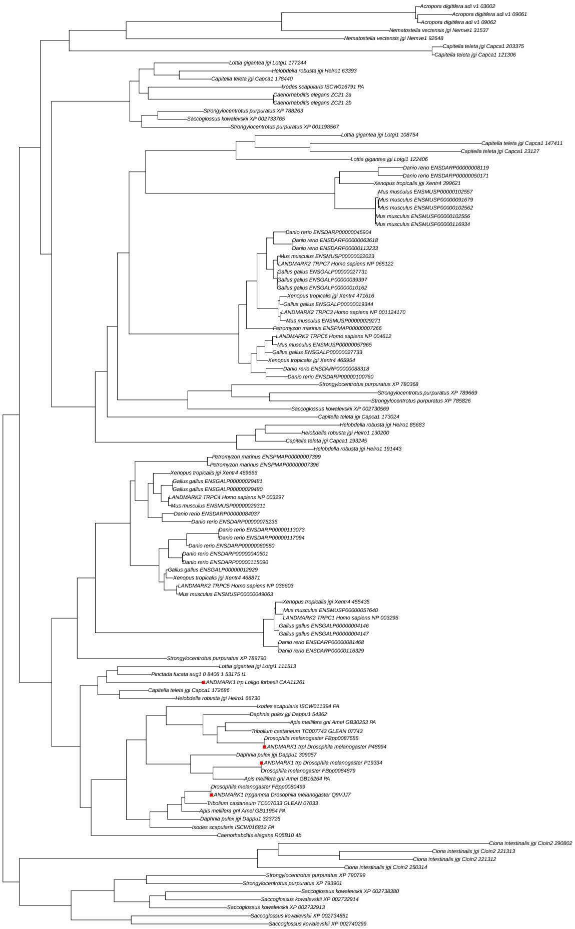

Tree File: Gq_beta

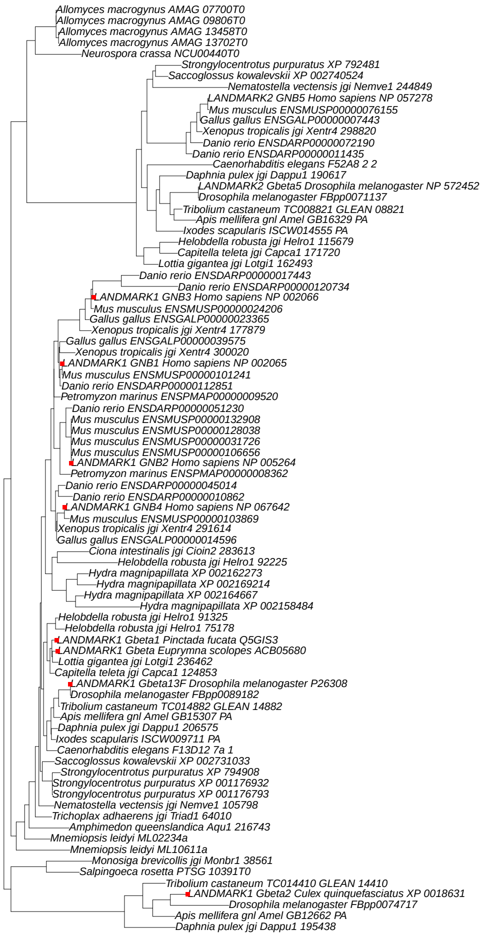

Tree File: rdgB

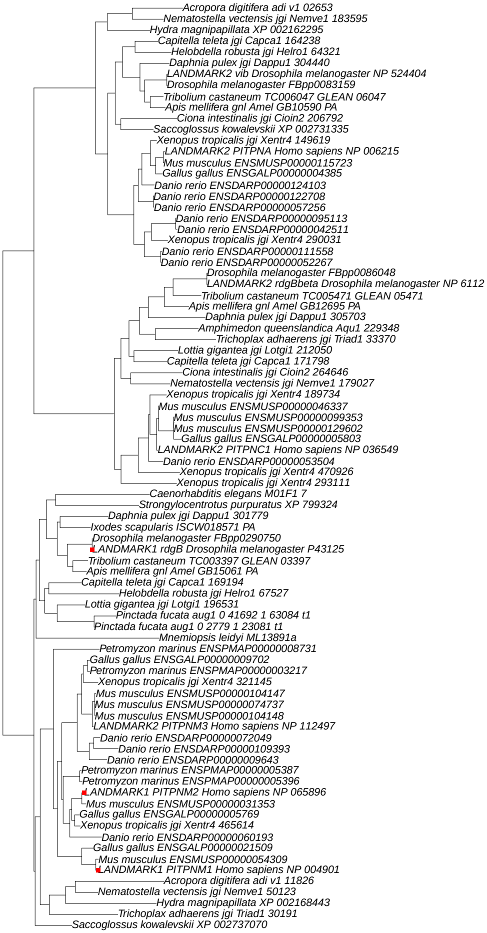

Tree File: PLC

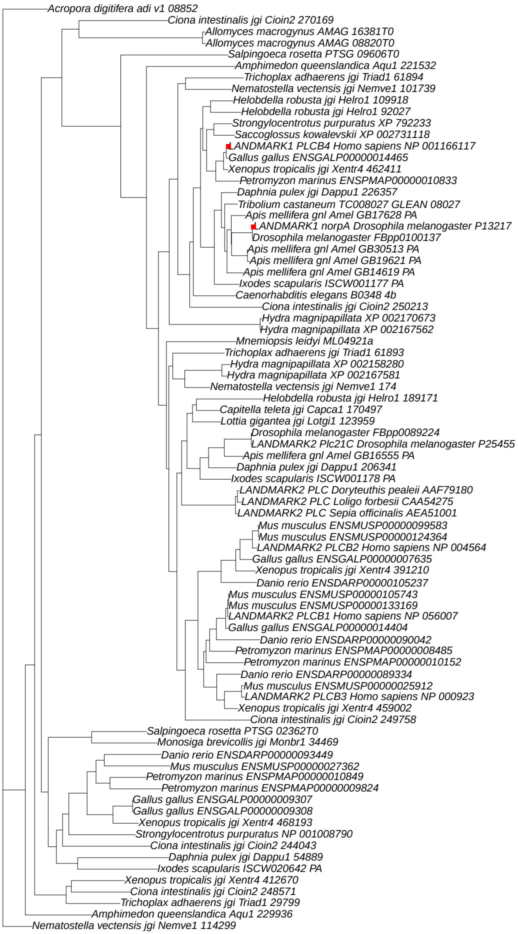

Tree File: Gprk2

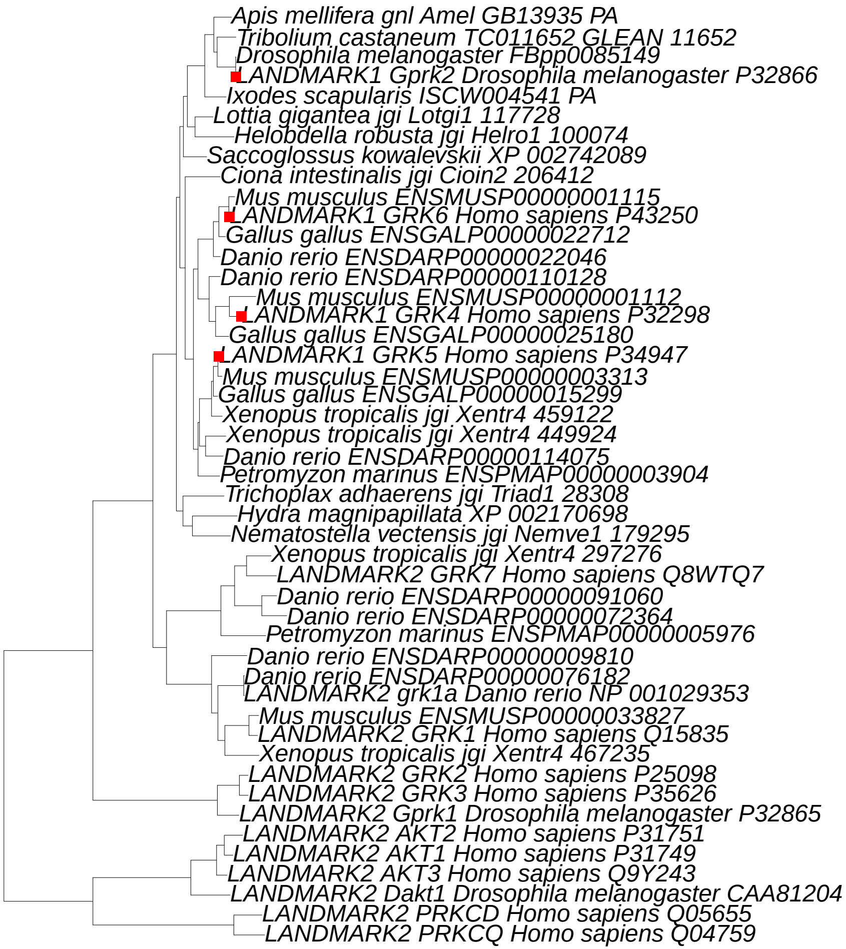

Tree File: Gq_gamma

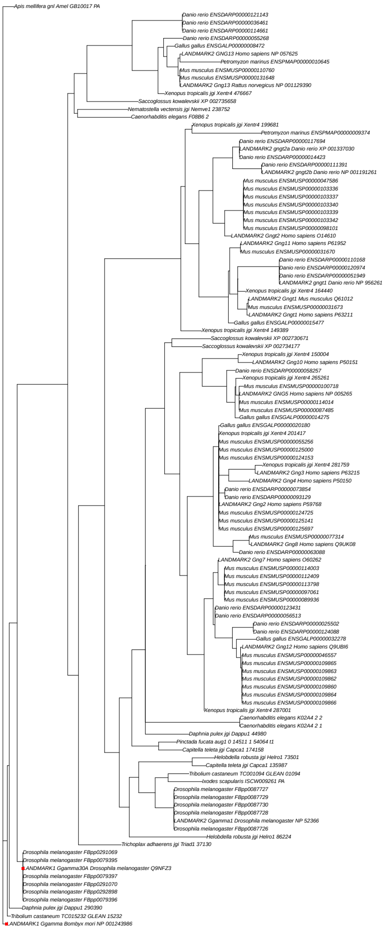

Tree File: Gq_alpha

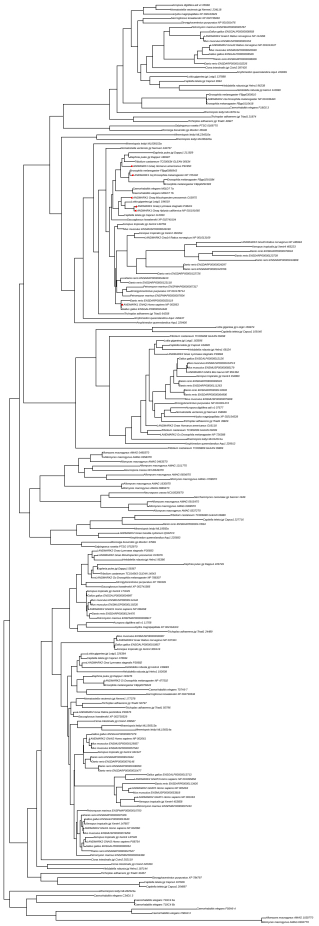

Tree File: Gprk1

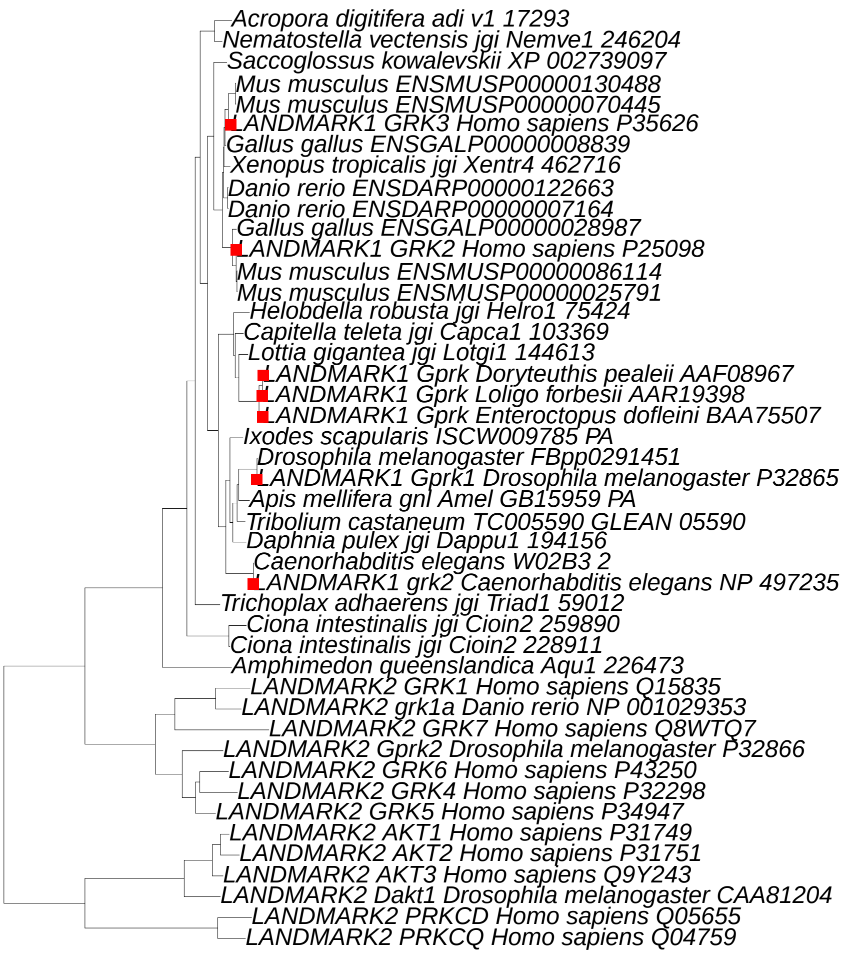

Tree File: DAGK

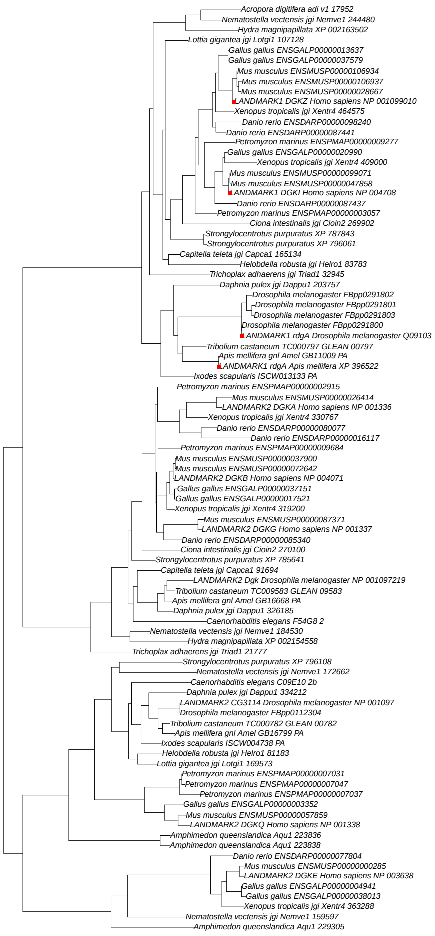

Tree File: Arr

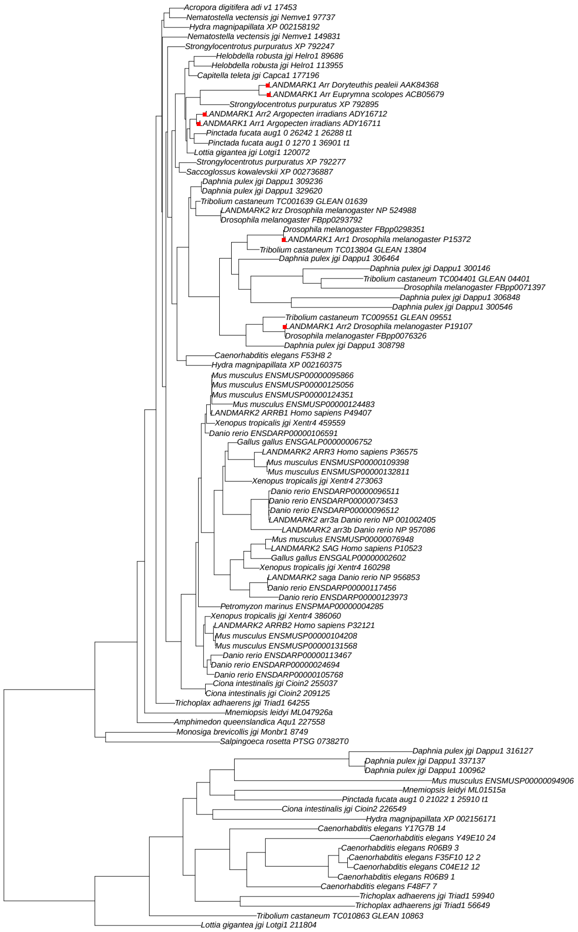

Tree File: rdgC

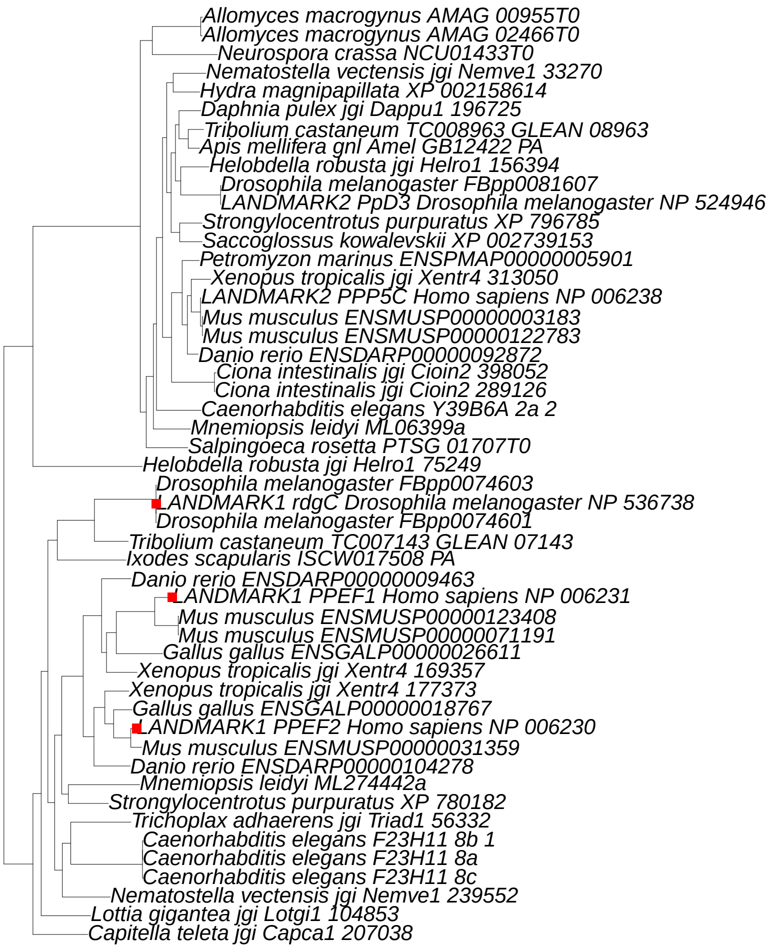

Tree File: PKC

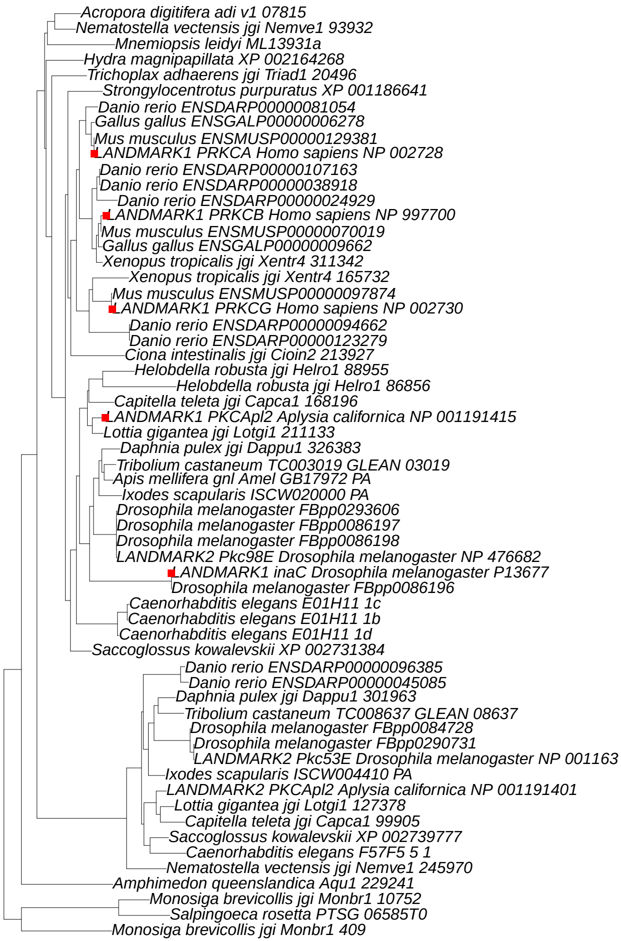

Tree File: Rcvrn

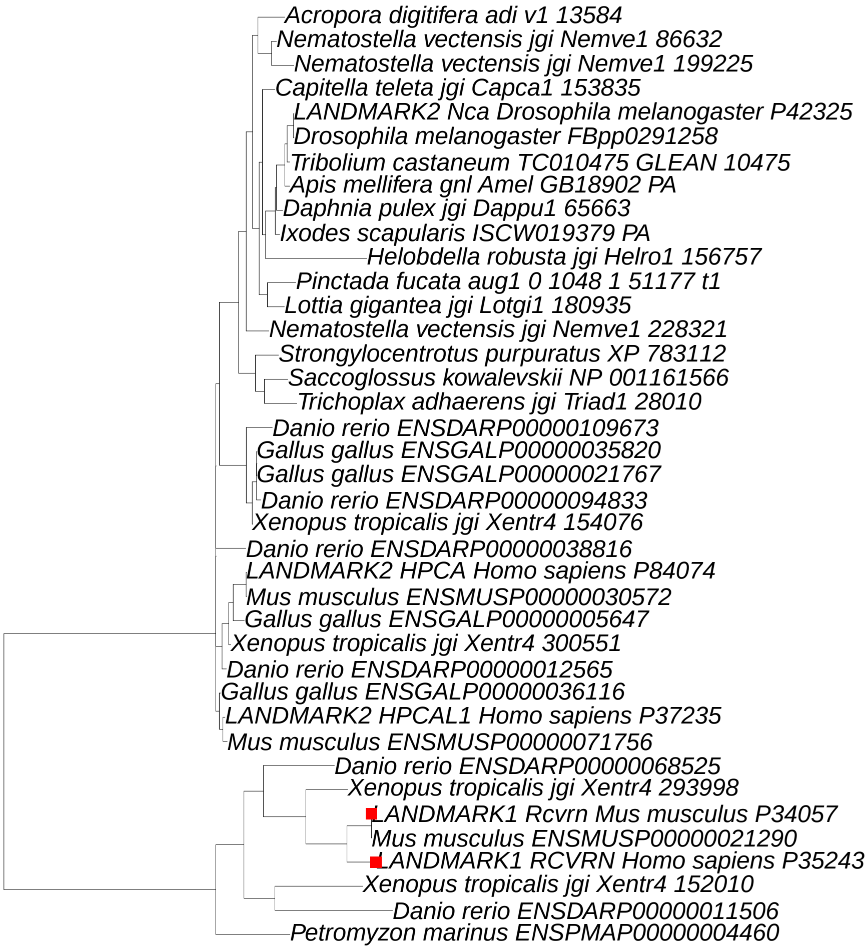

Tree File: RGS9

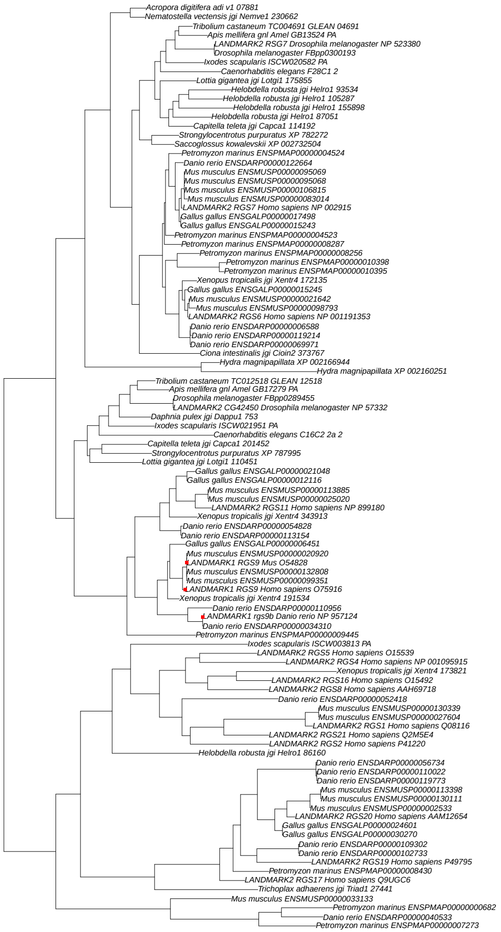

Tree File: Grk1

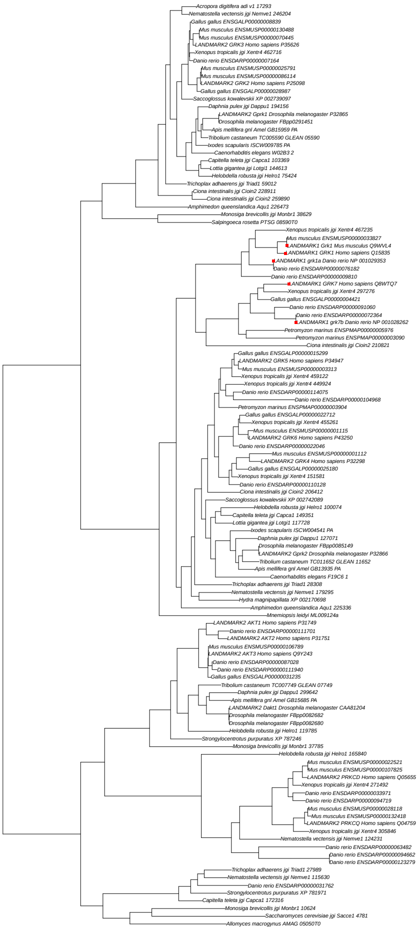

Tree File: Gngt1

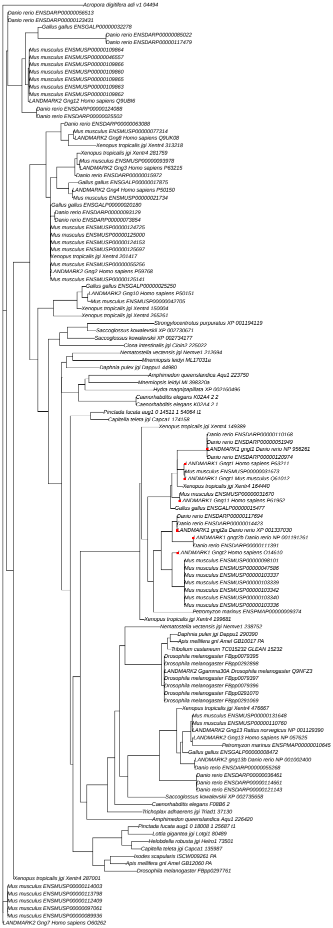

Tree File: Sag

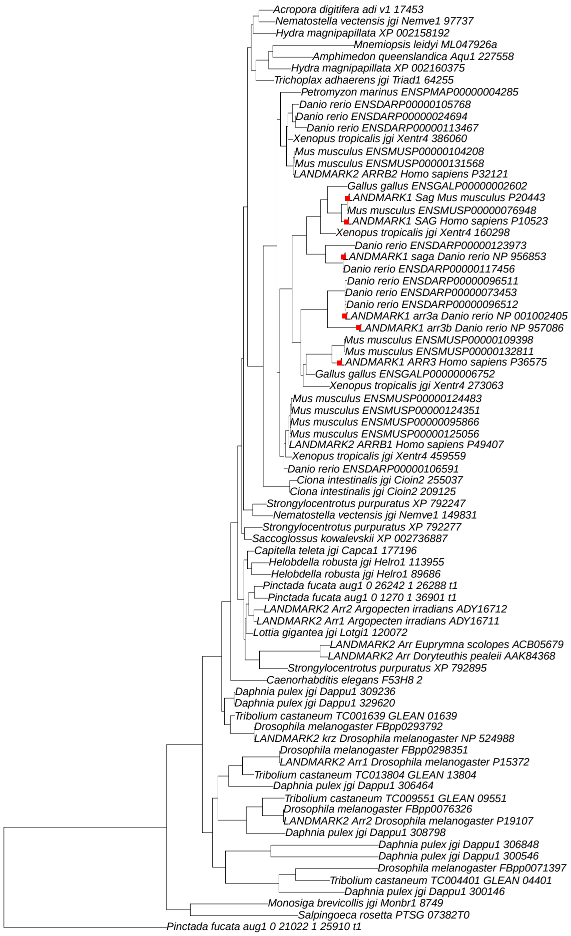

Tree File: Rgs9bp

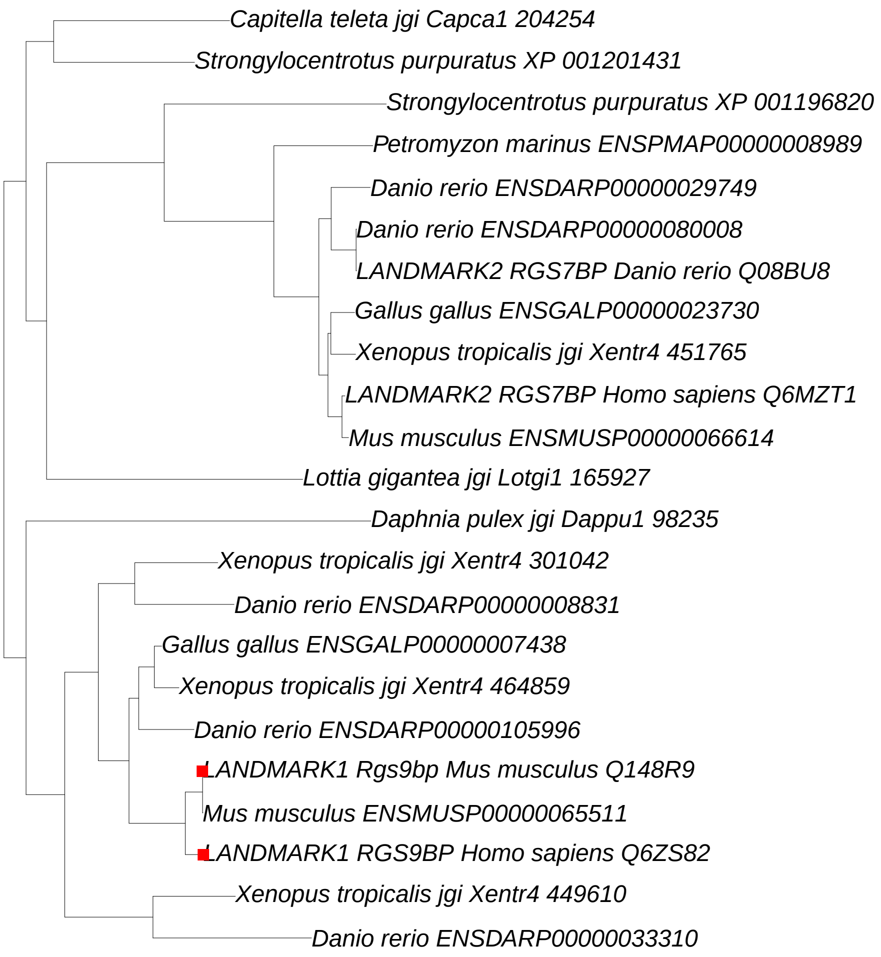

Tree File: GC

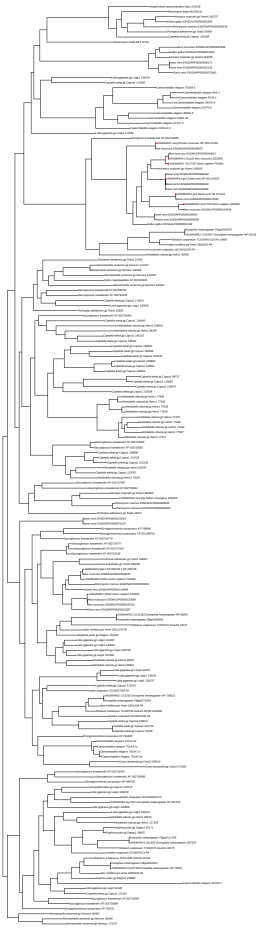

Tree File: Pde6d

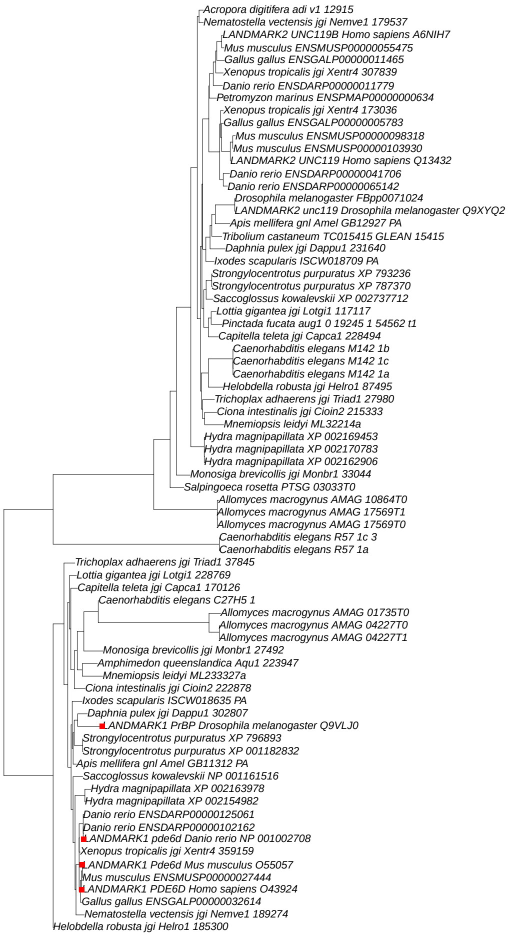

Tree File: Galpha_it

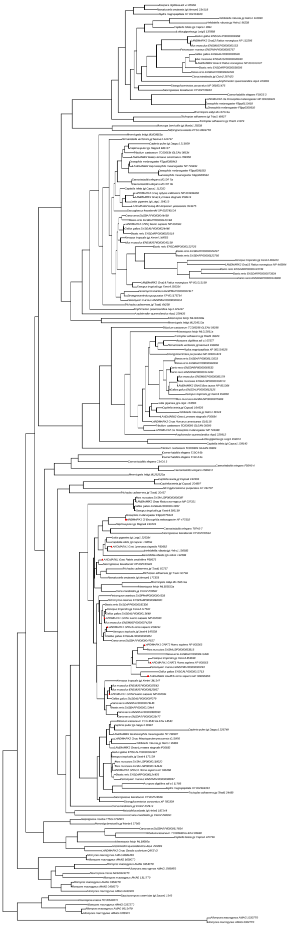

Tree File: c_opsin

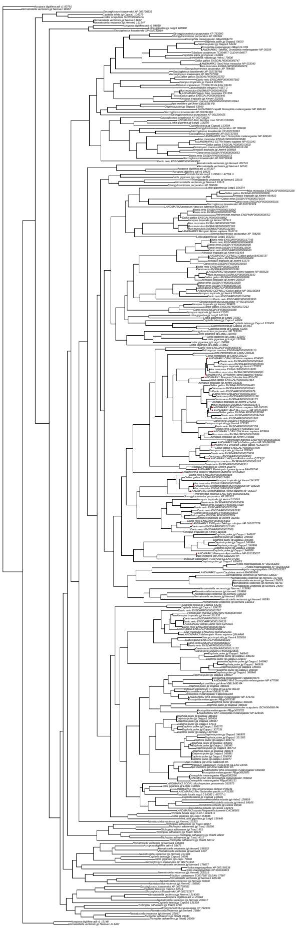

Tree File: Pde6abc

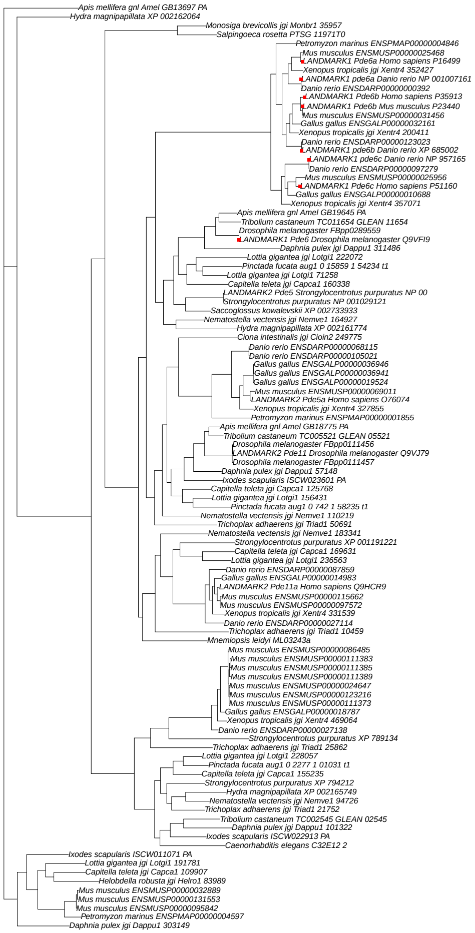

Tree File: Cnga1

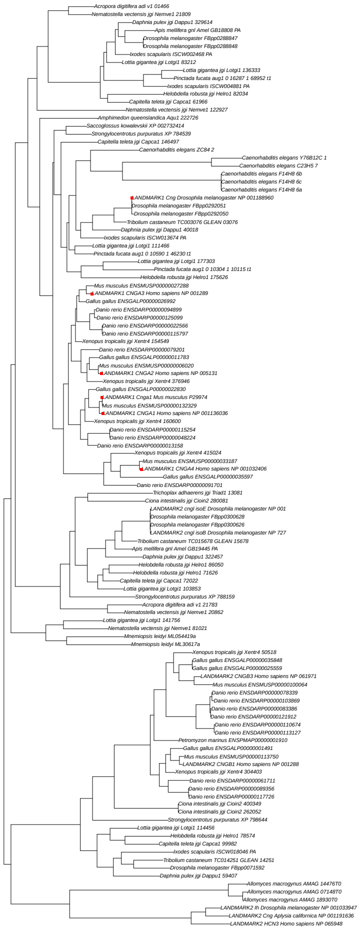

Tree File: RPE65

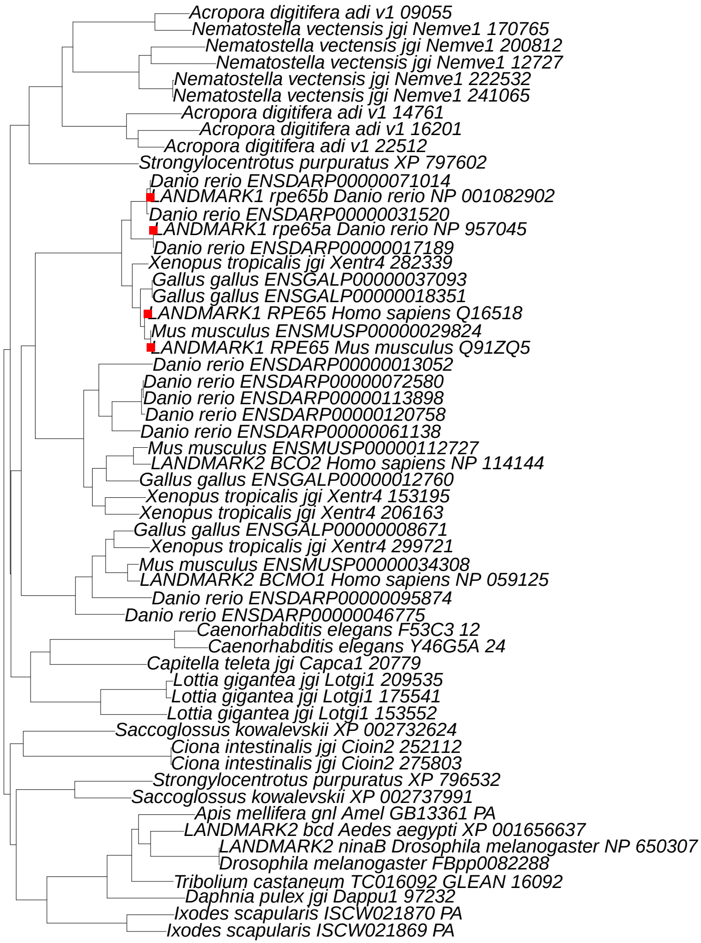

Tree File: RLBP1

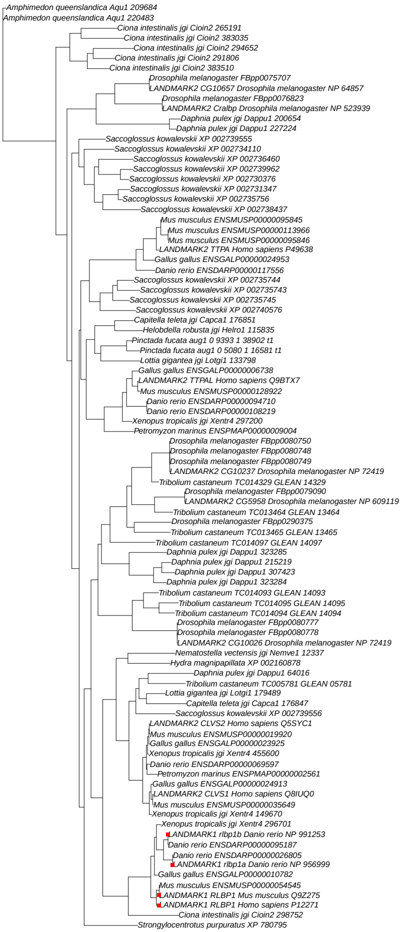

Tree File: RDH5

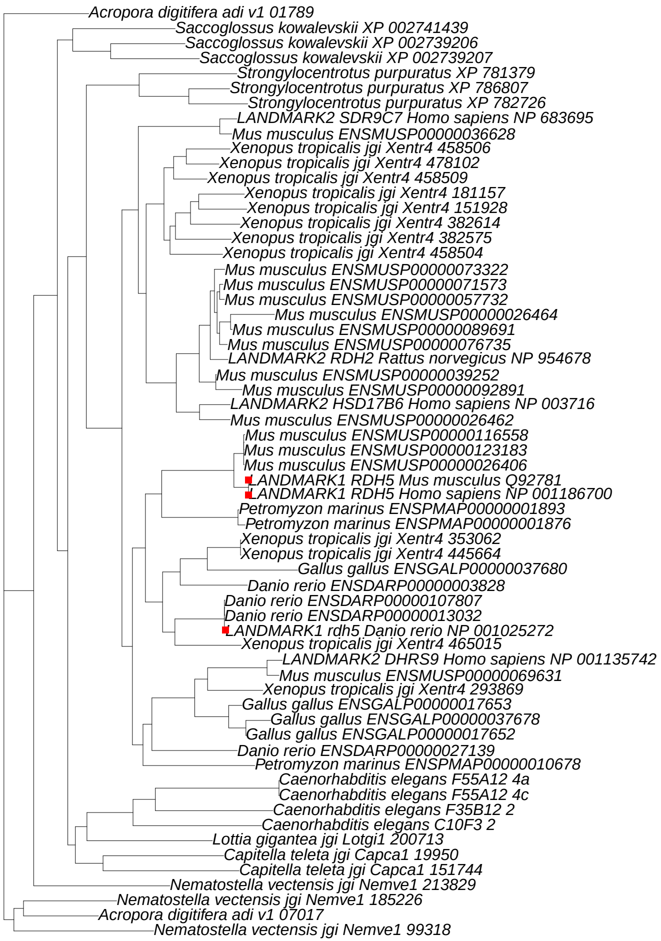

Tree File: LRAT

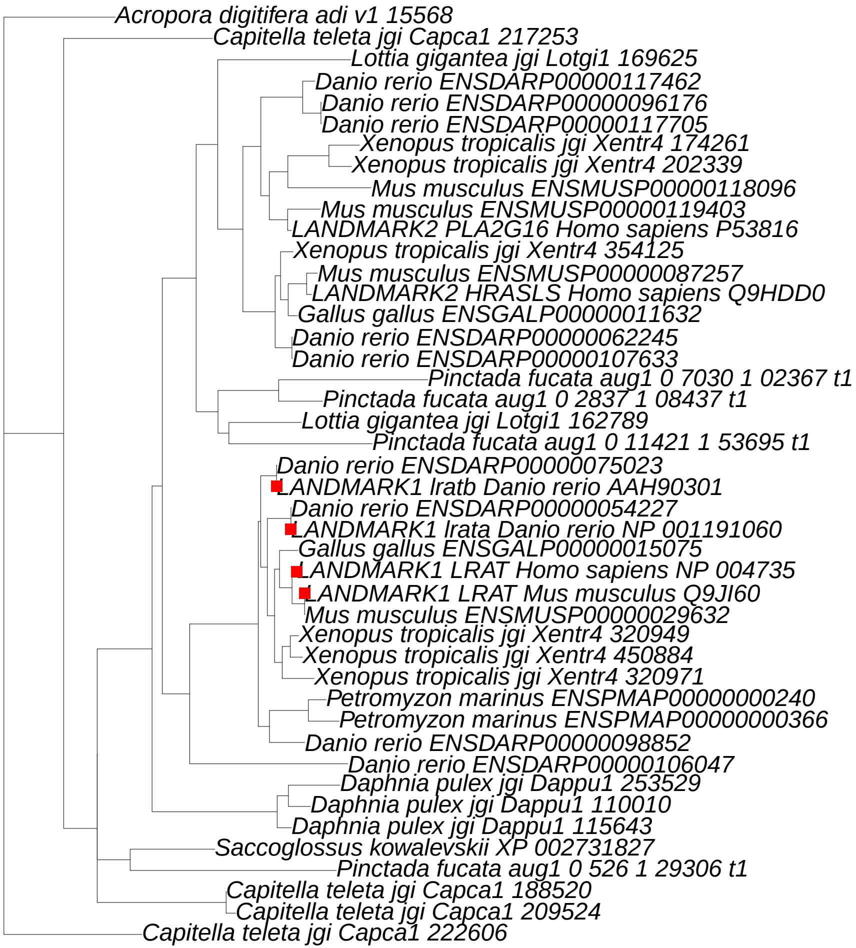

Tree File: RBP3

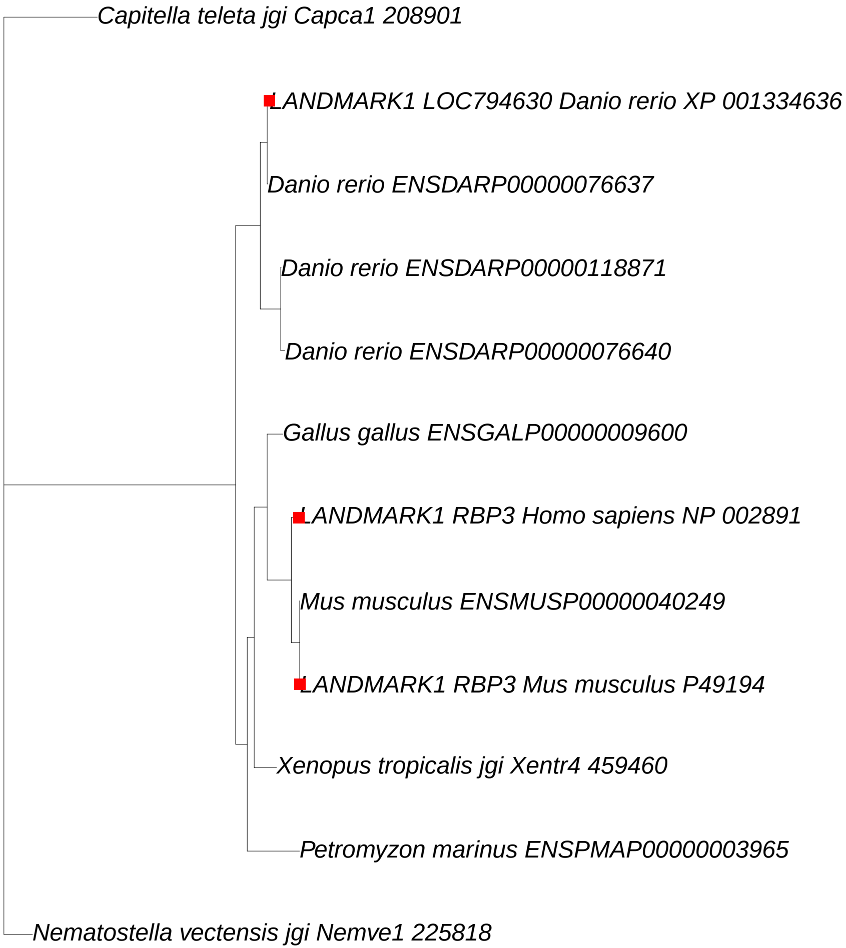

Tree File: RBP1

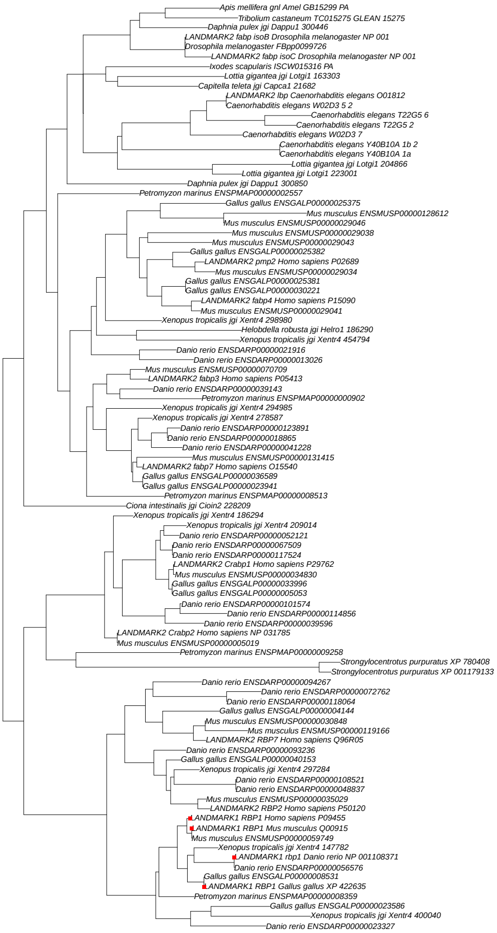

Tree File: RDH8

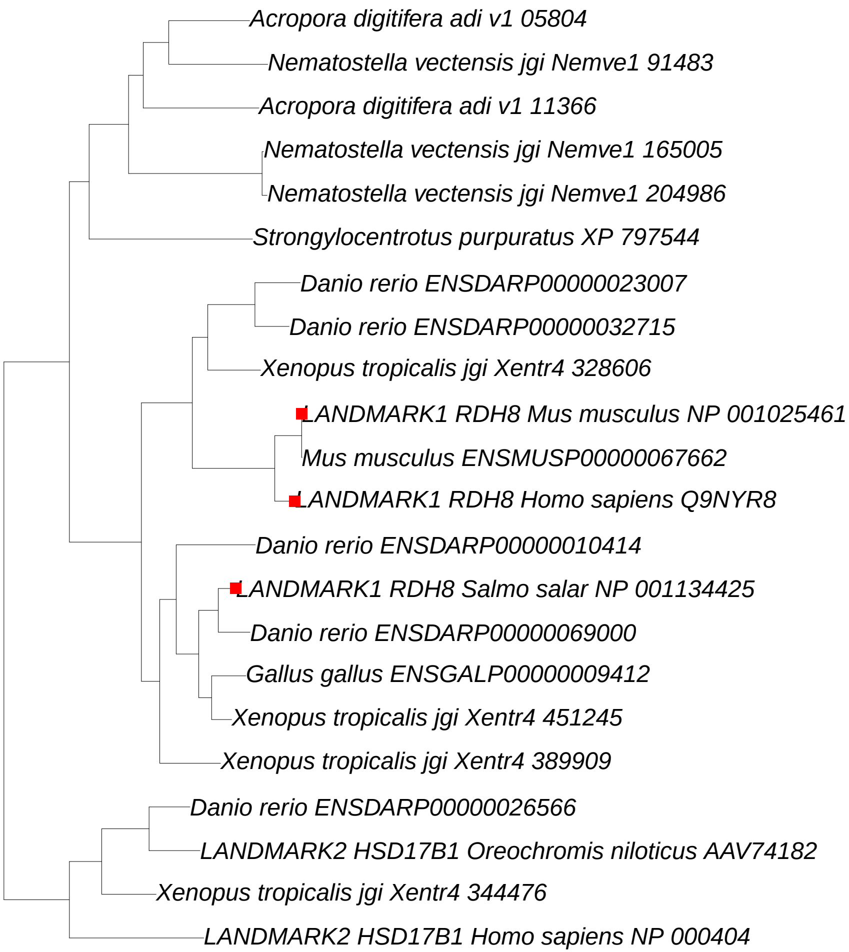

Tree File: ABCA4

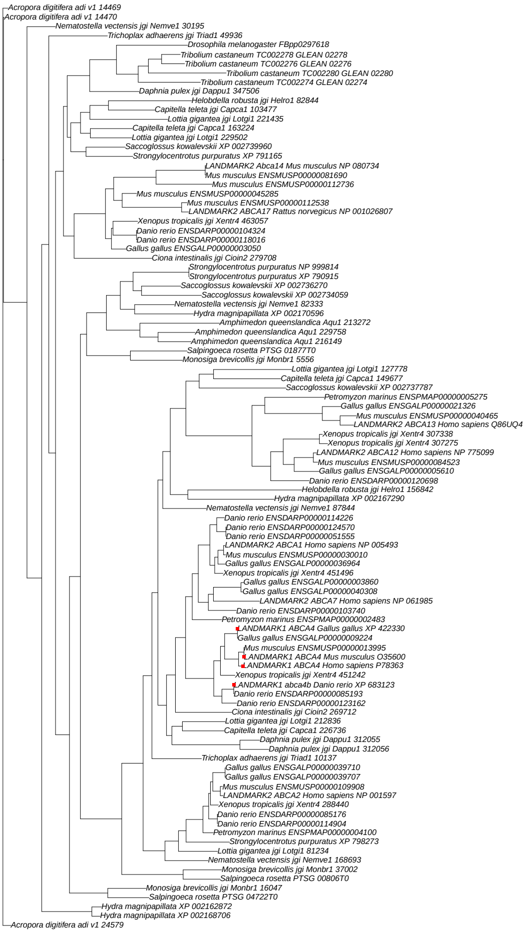

Tree File: RALBP

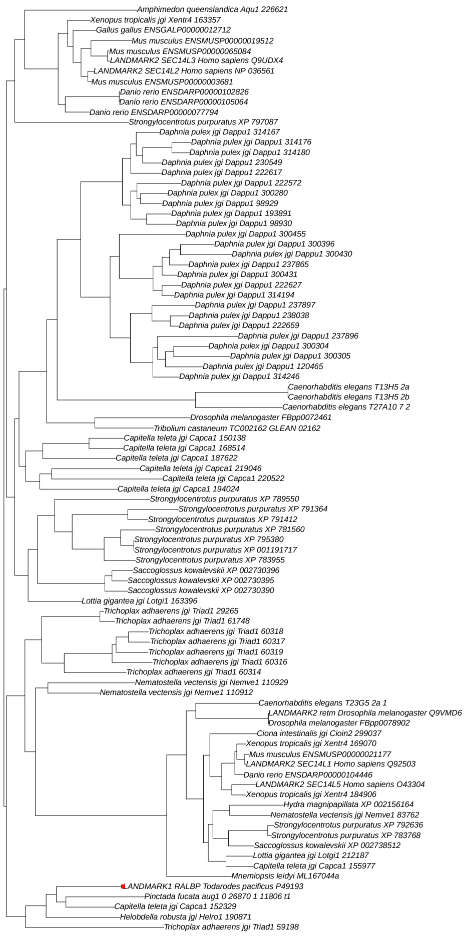

Tree File: pinta

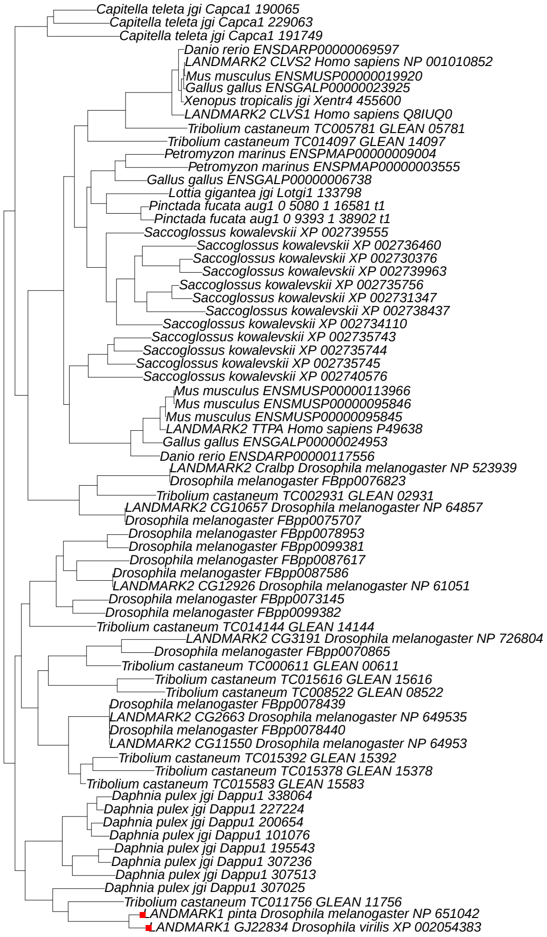

Tree File: ninaG

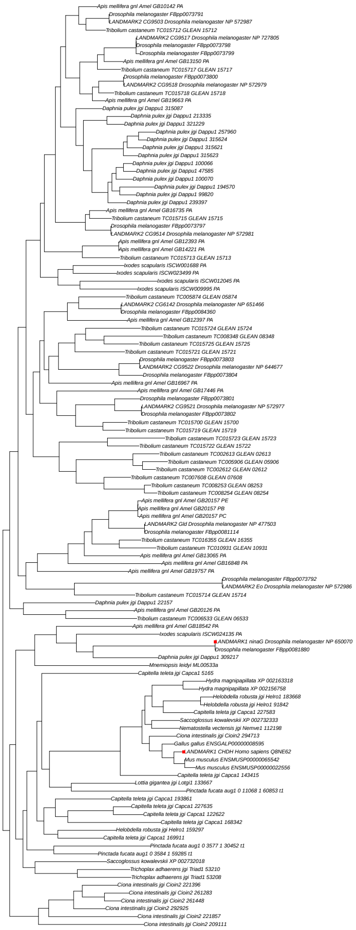

Tree File: ninaD

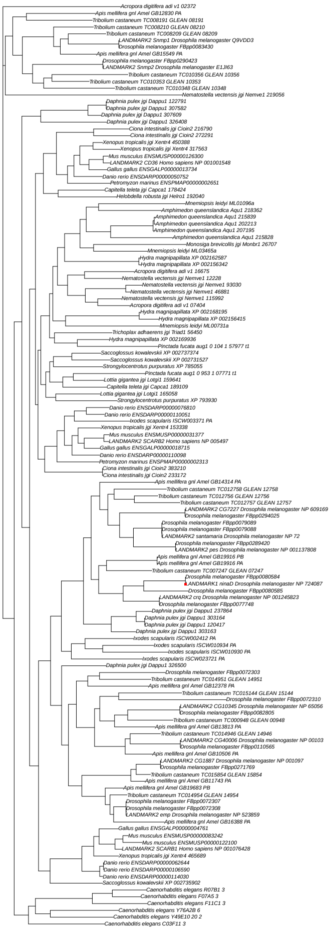

Tree File: ninaB

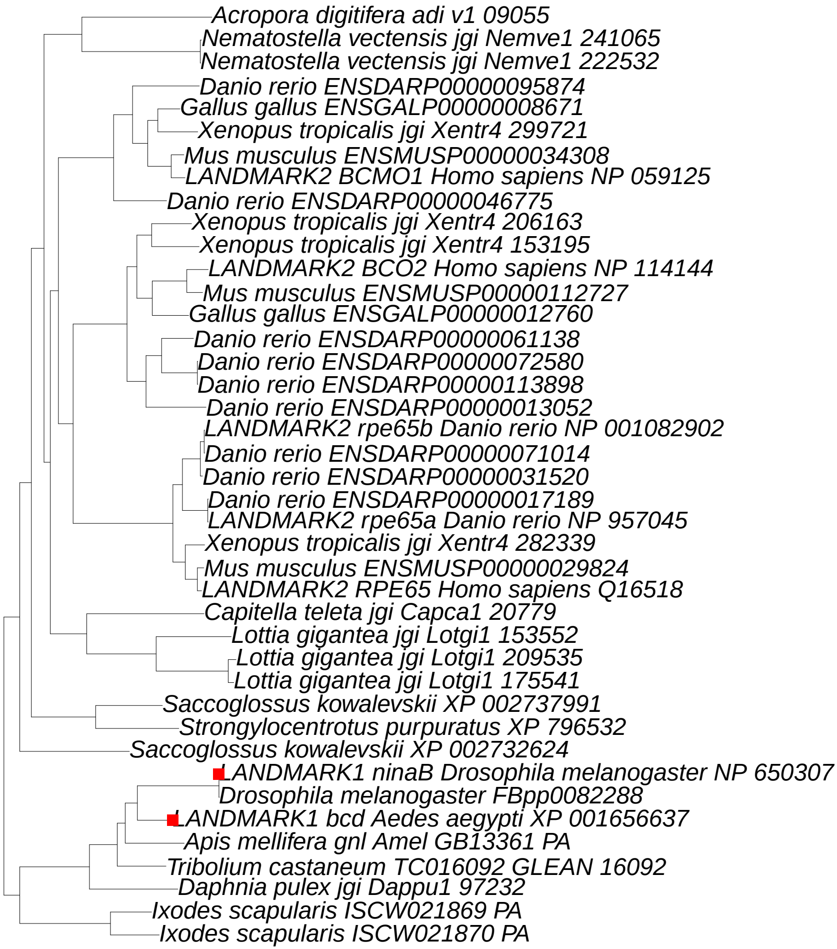

Tree File: santamaria

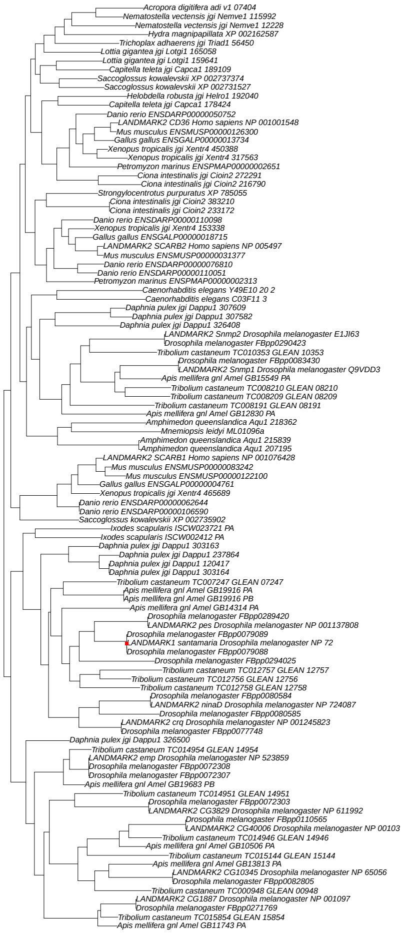

Tree File: DCE

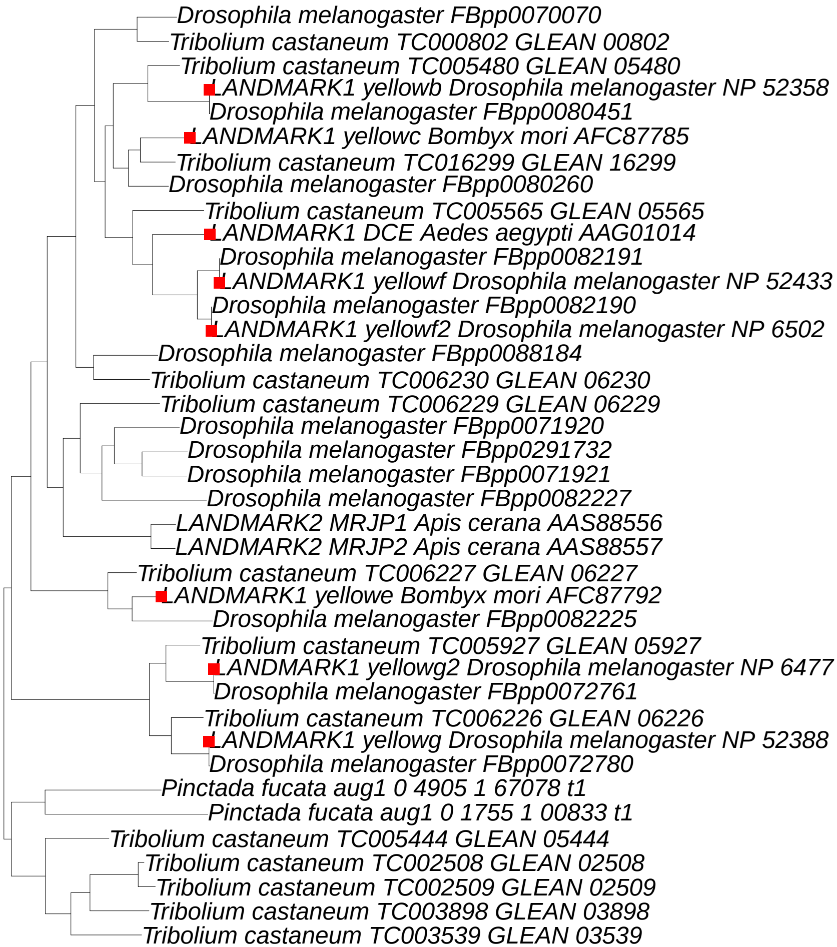

Tree File: laccase2

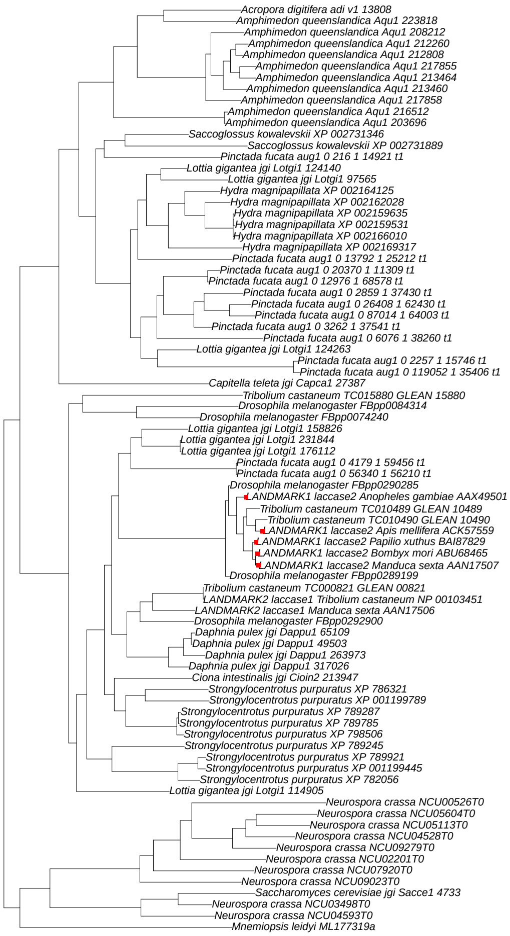

Tree File: TYR

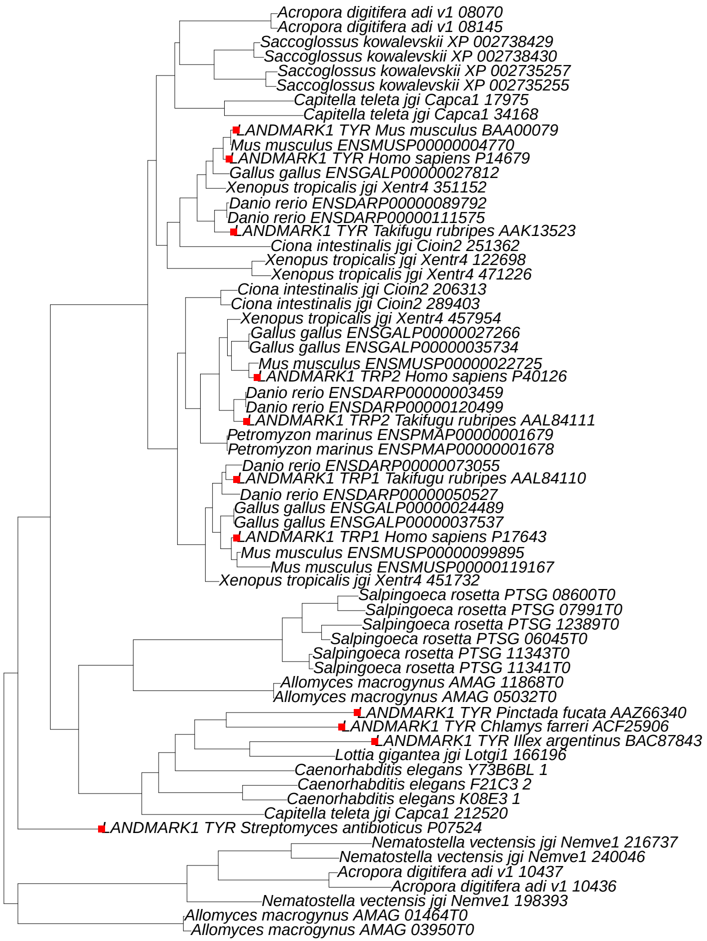

Tree File: DDC

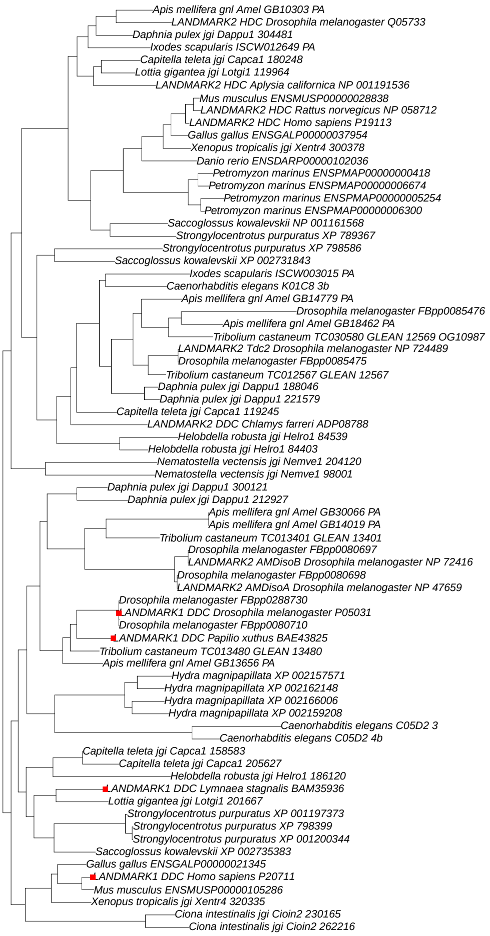

Tree File: TH

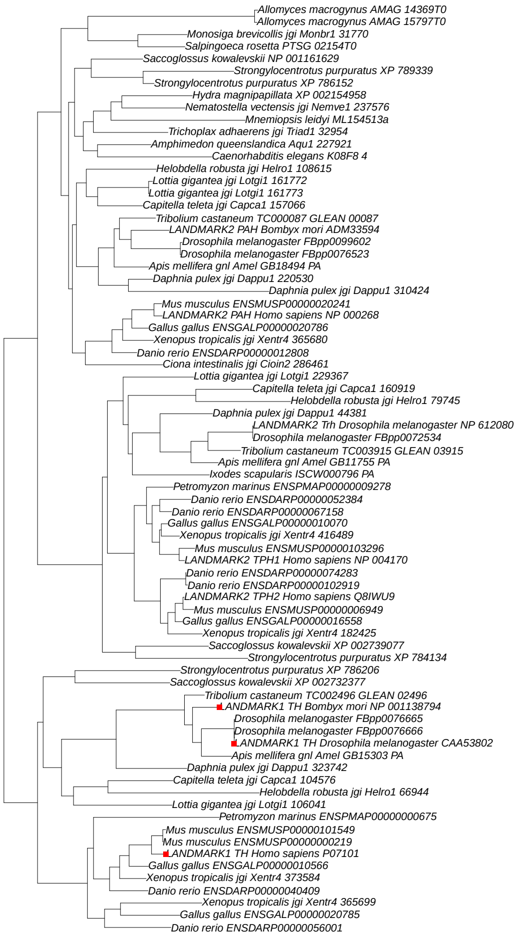

Allomyces macrogynus AMAG 14369T0
Allomyces macrogynus AMAG 15797T0
Monosiga brevicollis jgi Monbr1 31770
Salpingoeca rosetta PTSG 02154T0
Saccoglossus kowalevskii NP 001161629
Strongylocentrotus purpuratus XP 789339
Strongylocentrotus purpuratus XP 786152
Hydra magnipapillata XP 002154958
Nematostella vectensis jgi Nemve1 237576
Mnemiopsis leidyi ML154513a
Trichoplax adhaerens jgi Triad1 32954
Amphimedon queenslandica Aqu1 227921
Caenorhabditis elegans K08F8 4
Helobdella robusta jgi Helro1 108615
Lottia gigantea jgi Lotgi1 161772
Lottia gigantea jgi Lotgi1 161773
Capitella teleta jgi Capca1 157066
Tribolium castaneum TC000087 GLEAN 00087
LANDMARK2 PAH Bombyx mori ADM33594
Drosophila melanogaster FBpp0099602
Drosophila melanogaster FBpp0076523
Apis mellifera gnl Amel GB18494 PA
Daphnia pulex jgi Dappu1 220530
Daphnia pulex jgi Dappu1 310424
Mus musculus ENSMUSP00000020241
LANDMARK2 PAH Homo sapiens NP 000268
Gallus gallus ENSGALP00000020786
Xenopus tropicalis jgi Xentr4 365680
Danio rerio ENSDARP00000012808
Ciona intestinalis jgi Cioin2 286461
Lottia gigantea jgi Lotgi1 229367
Capitella teleta jgi Capca1 160919
Helobdella robusta jgi Helro1 79745
Daphnia pulex jgi Dappu1 44381
LANDMARK2 Trh Drosophila melanogaster NP 612080
Drosophila melanogaster FBpp0072534
Tribolium castaneum TC003915 GLEAN 03915
Apis mellifera gnl Amel GB11755 PA
Ixodes scapularis ISCW000796 PA
Petromyzon marinus ENSPMAP00000009278
Danio rerio ENSDARP00000052384
Danio rerio ENSDARP00000067158
Gallus gallus ENSGALP00000010070
Xenopus tropicalis jgi Xentr4 416489
Mus musculus ENSMUSP00000103296
LANDMARK2 TPH1 Homo sapiens NP 004170
Danio rerio ENSDARP00000074283
Danio rerio ENSDARP00000102919
LANDMARK2 TPH2 Homo sapiens Q8IWU9
Mus musculus ENSMUSP00000006949
Gallus gallus ENSGALP00000016558
Xenopus tropicalis jgi Xentr4 182425
Saccoglossus kowalevskii XP 002739077
Strongylocentrotus purpuratus XP 784134
Strongylocentrotus purpuratus XP 786206
Saccoglossus kowalevskii XP 002732377
Tribolium castaneum TC002496 GLEAN 02496
LANDMARK1 TH Bombyx mori NP 001138794
Drosophila melanogaster FBpp0076665
Drosophila melanogaster FBpp0076666
LANDMARK1 TH Drosophila melanogaster CAA53802
Apis mellifera gnl Amel GB15303 PA
Daphnia pulex jgi Dappu1 323742
Capitella teleta jgi Capca1 104576
Helobdella robusta jgi Helro1 66944
Lottia gigantea jgi Lotgi1 106041
Petromyzon marinus ENSPMAP00000000675
Mus musculus ENSMUSP00000101549
Mus musculus ENSMUSP00000000219
LANDMARK1 TH Homo sapiens P07101
Gallus gallus ENSGALP00000010566
Xenopus tropicalis jgi Xentr4 373584
Danio rerio ENSDARP00000040409
Xenopus tropicalis jgi Xentr4 365699
Gallus gallus ENSGALP00000020785
Danio rerio ENSDARP00000056001

Tree File: NBAD

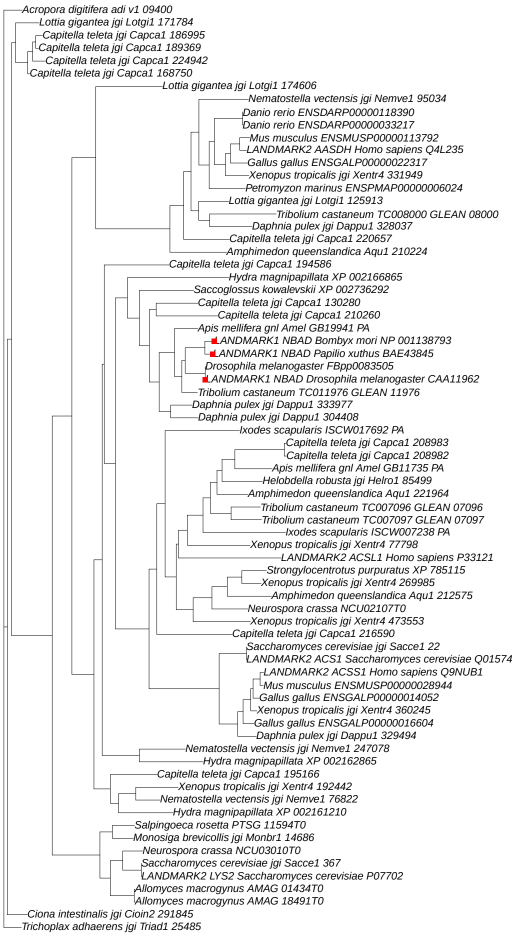

Tree File: CSAD

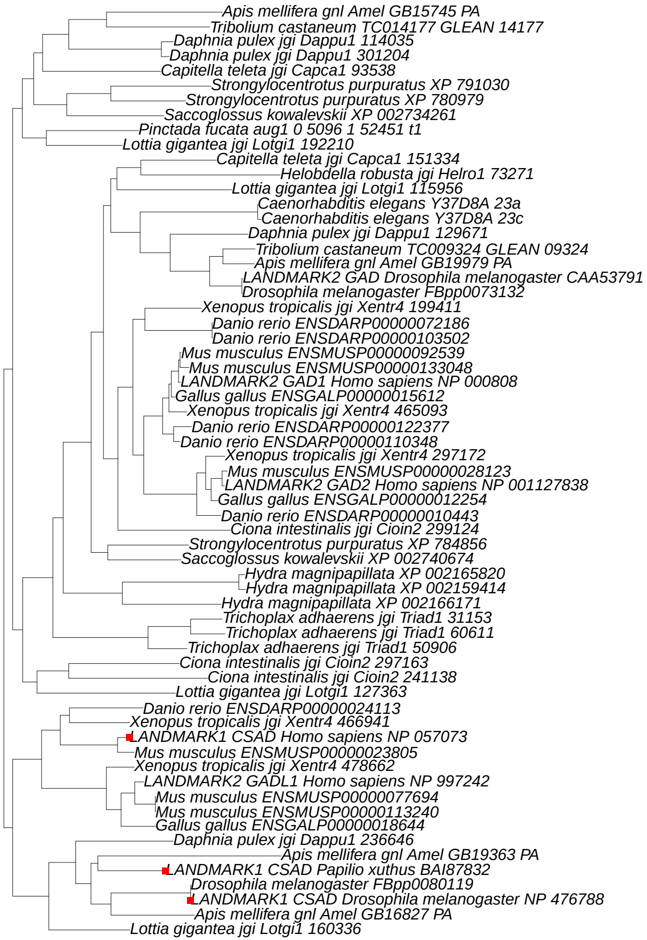

Tree File: PPO

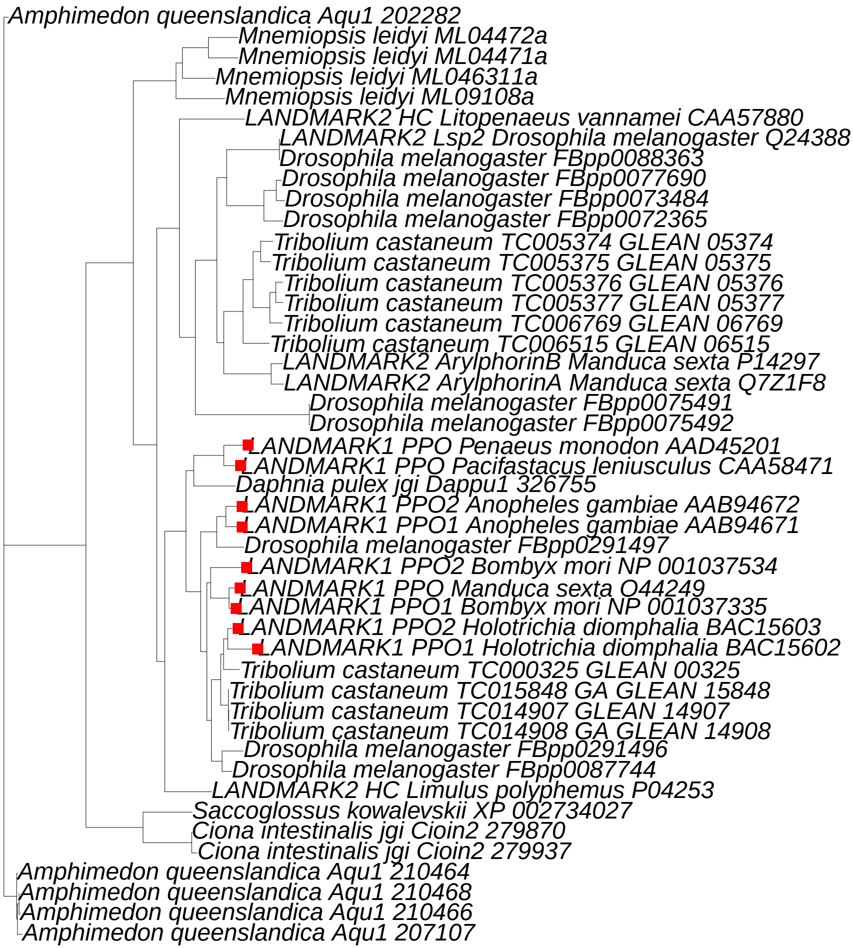

Tree File: tan

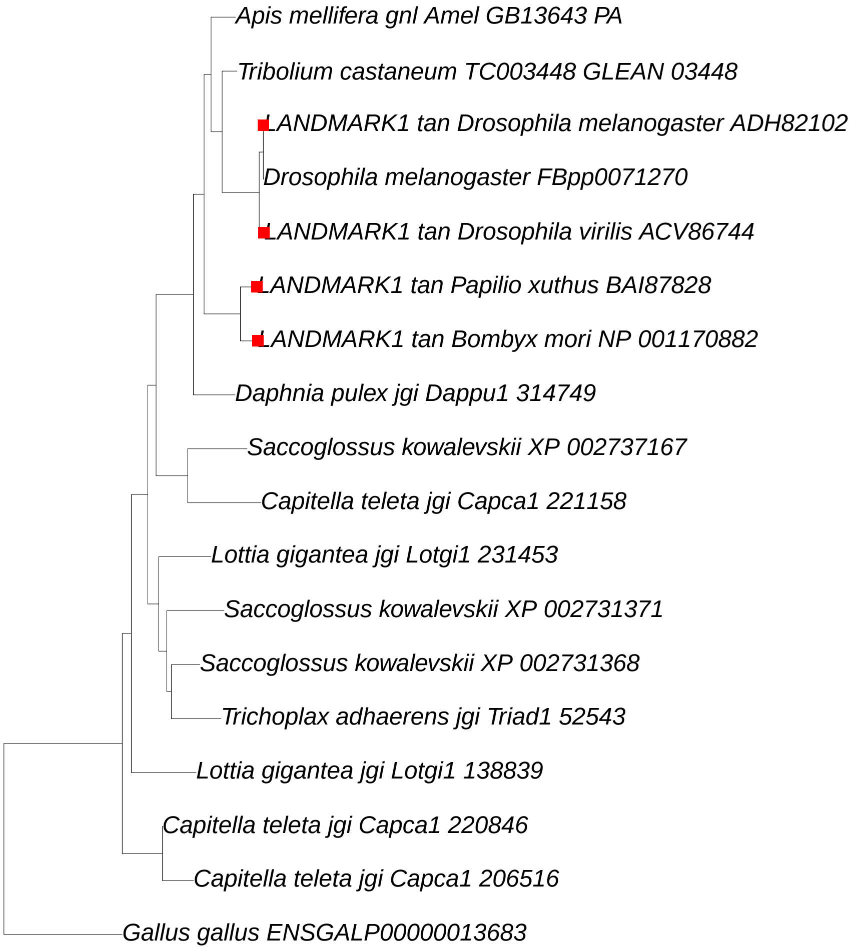

Tree File: Dat

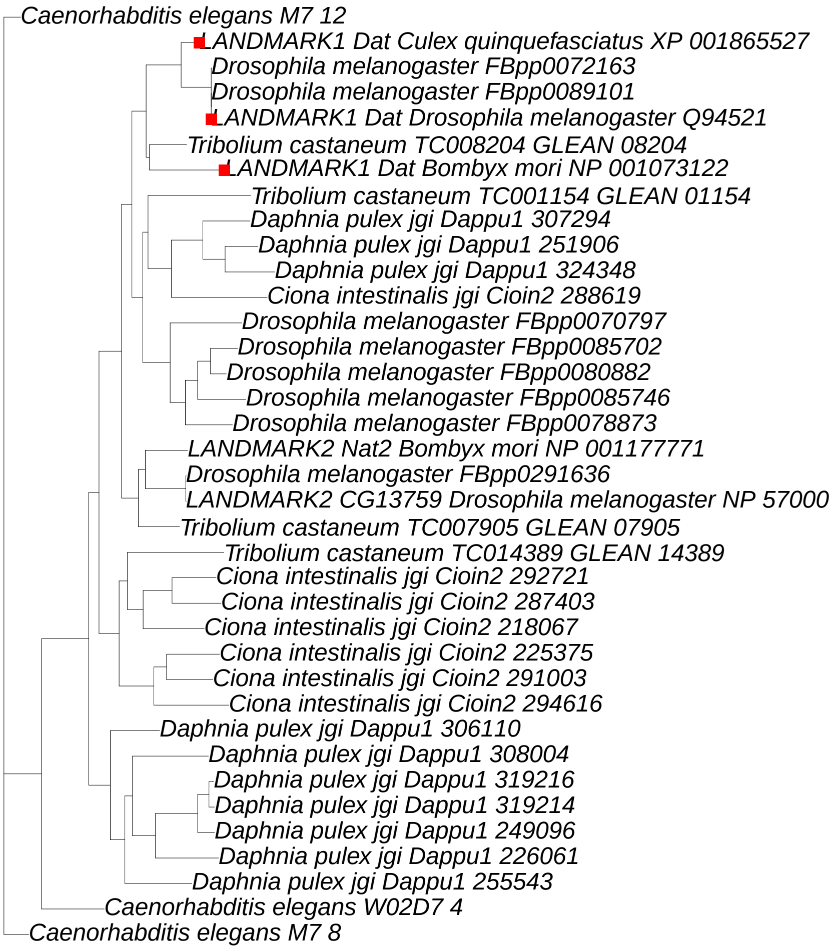

Tree File: GCH1

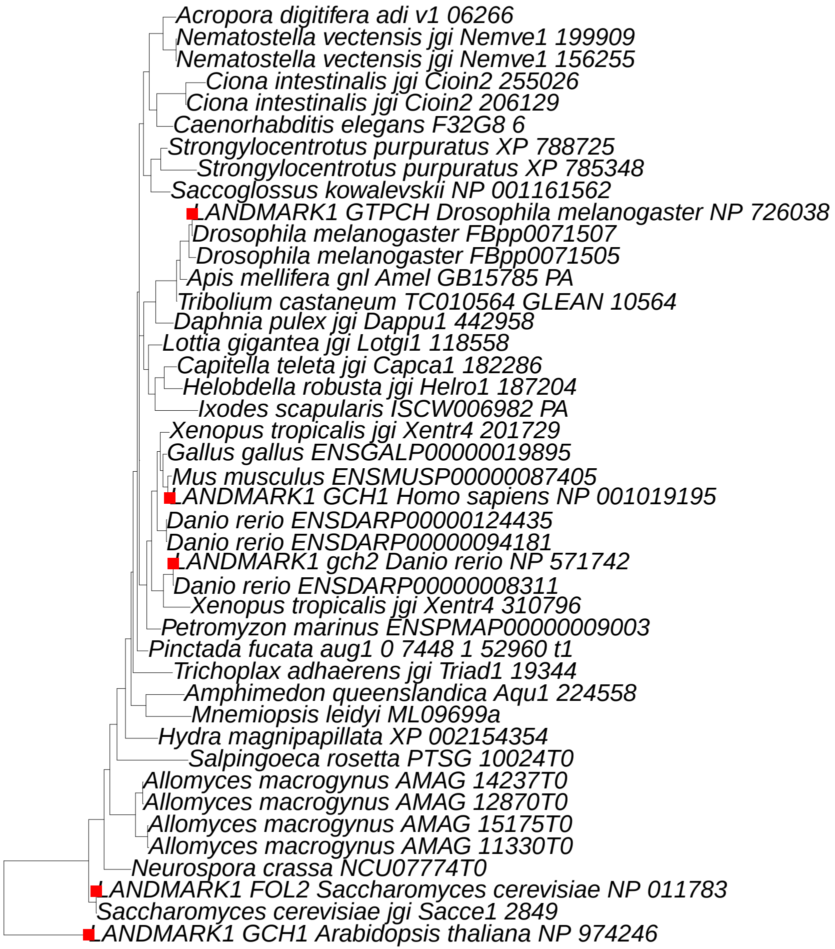

Tree File: XDH

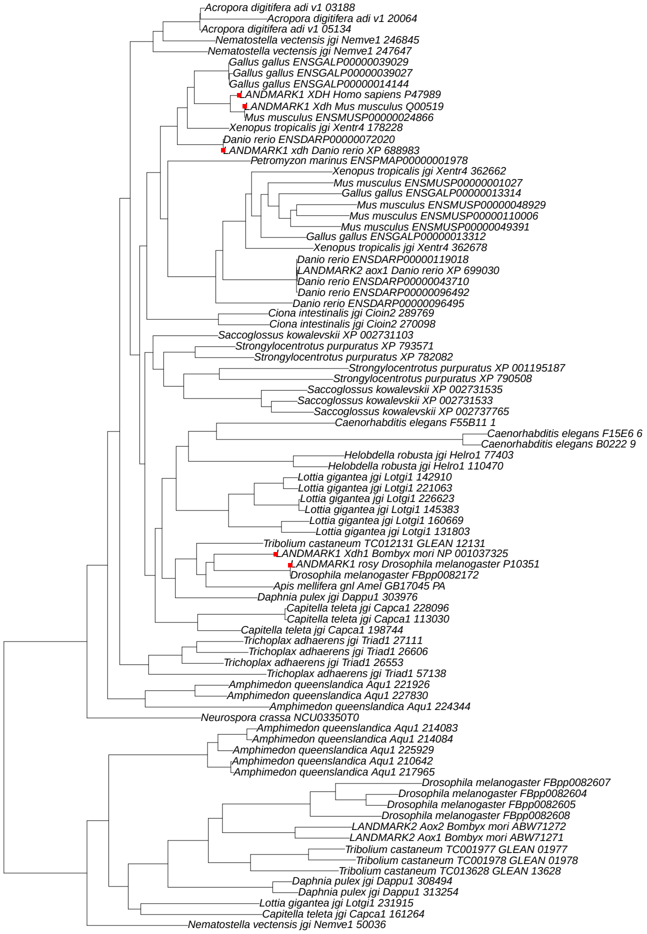

Tree File: Pcd

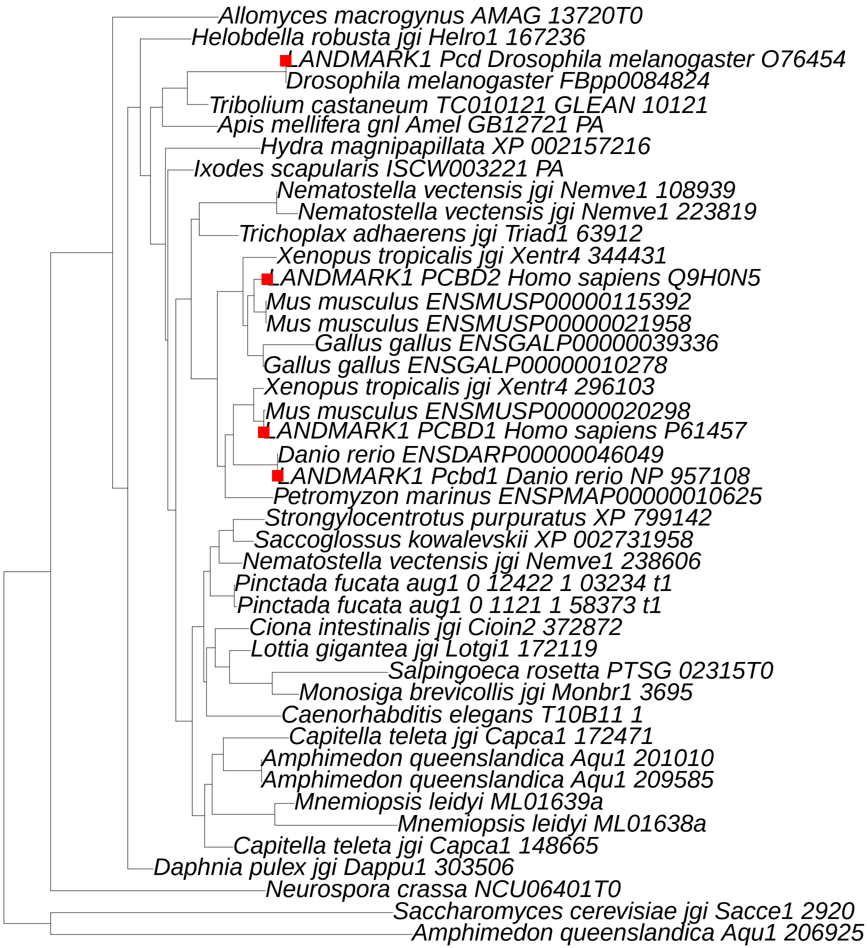

Tree File: PTS

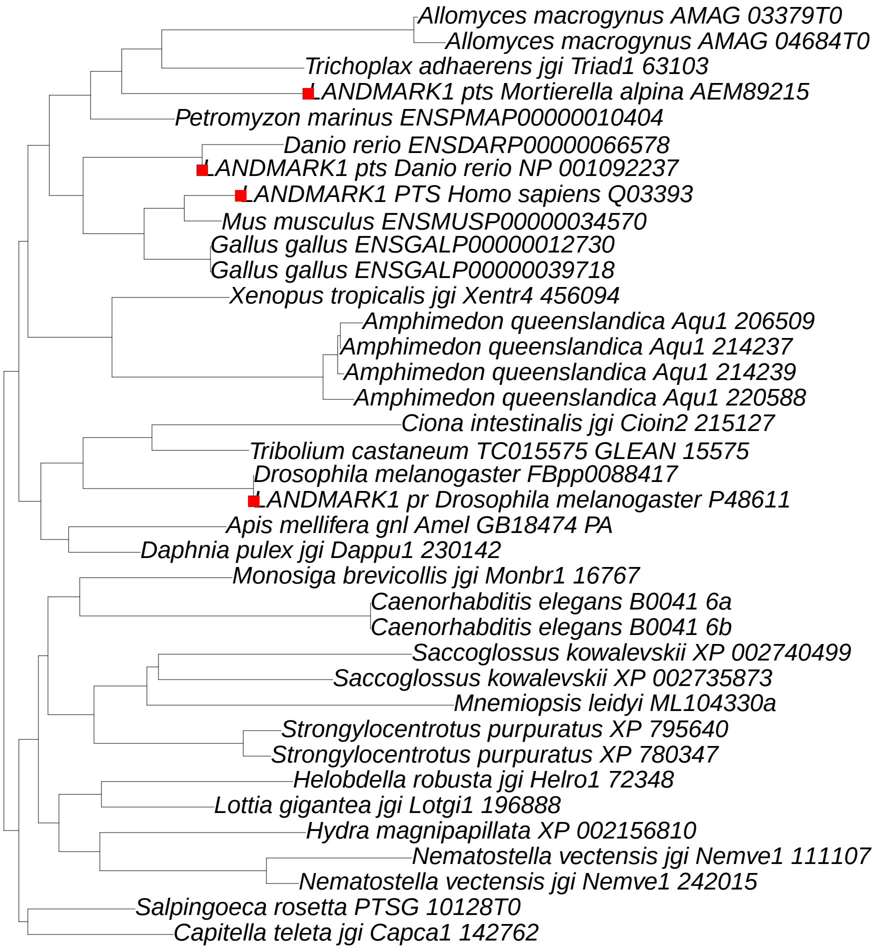

Tree File: clot

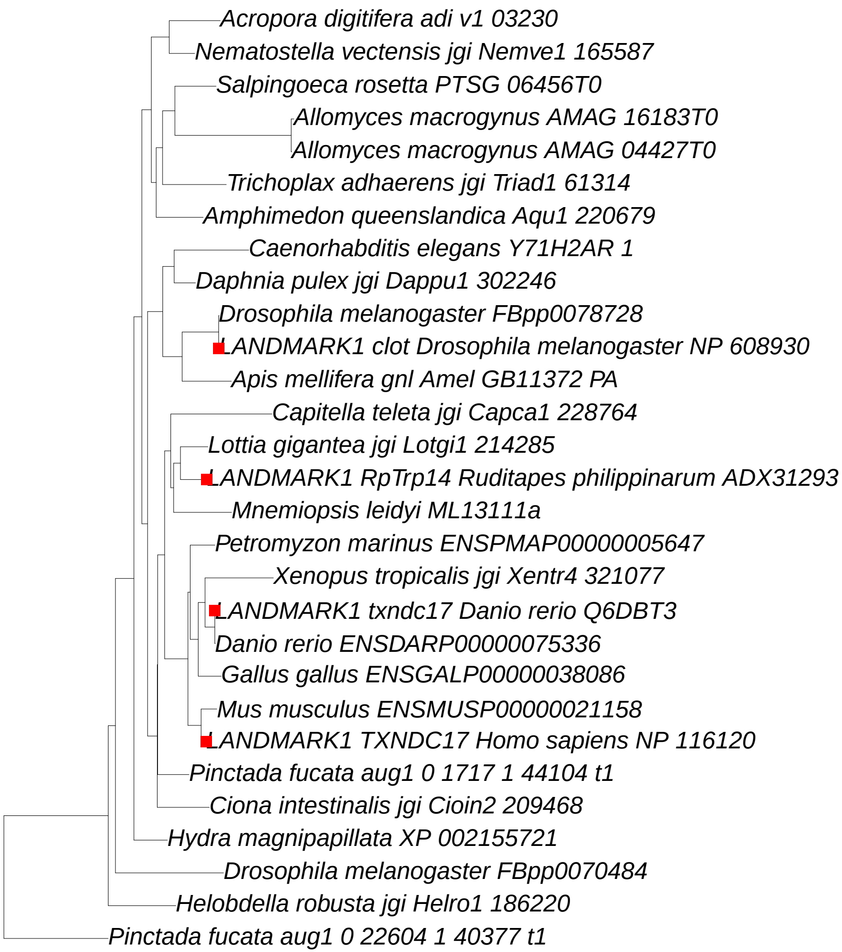

Tree File: sepia

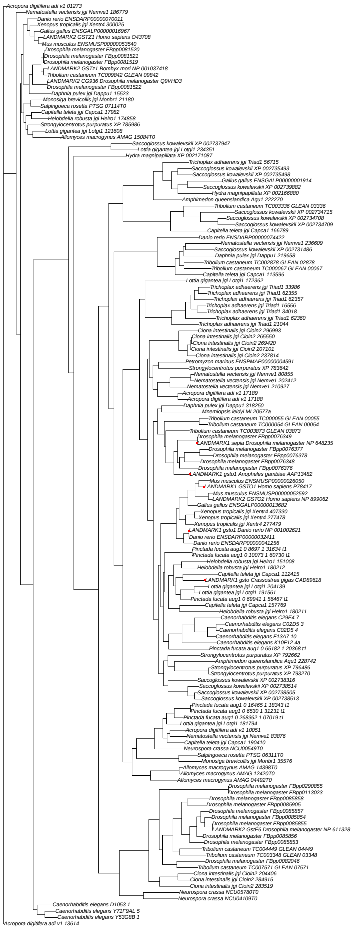

Tree File: Dhpr

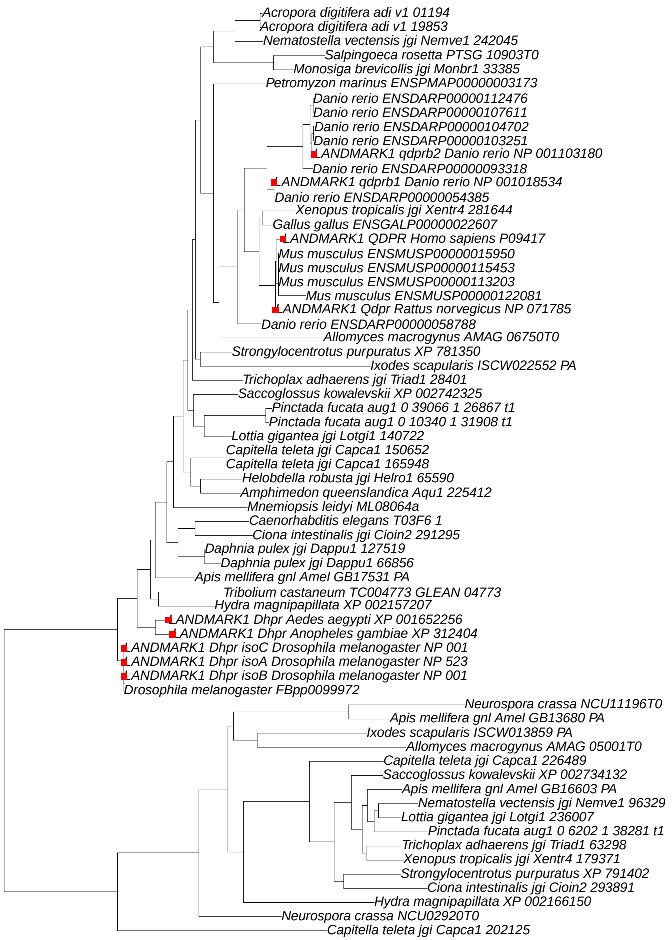

Tree File: SPR

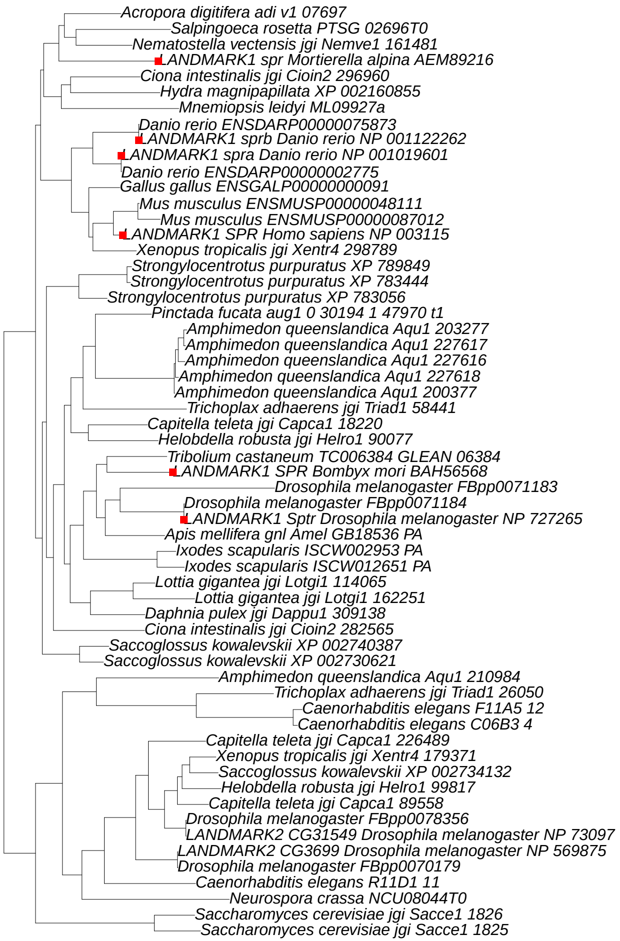

Tree File: oca2

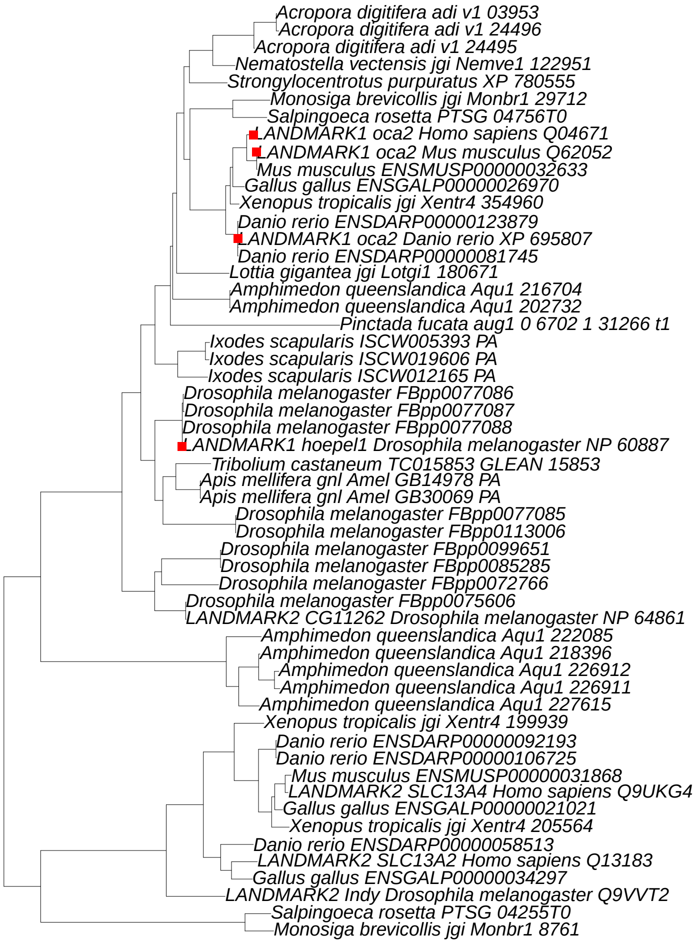

Tree File: TDO

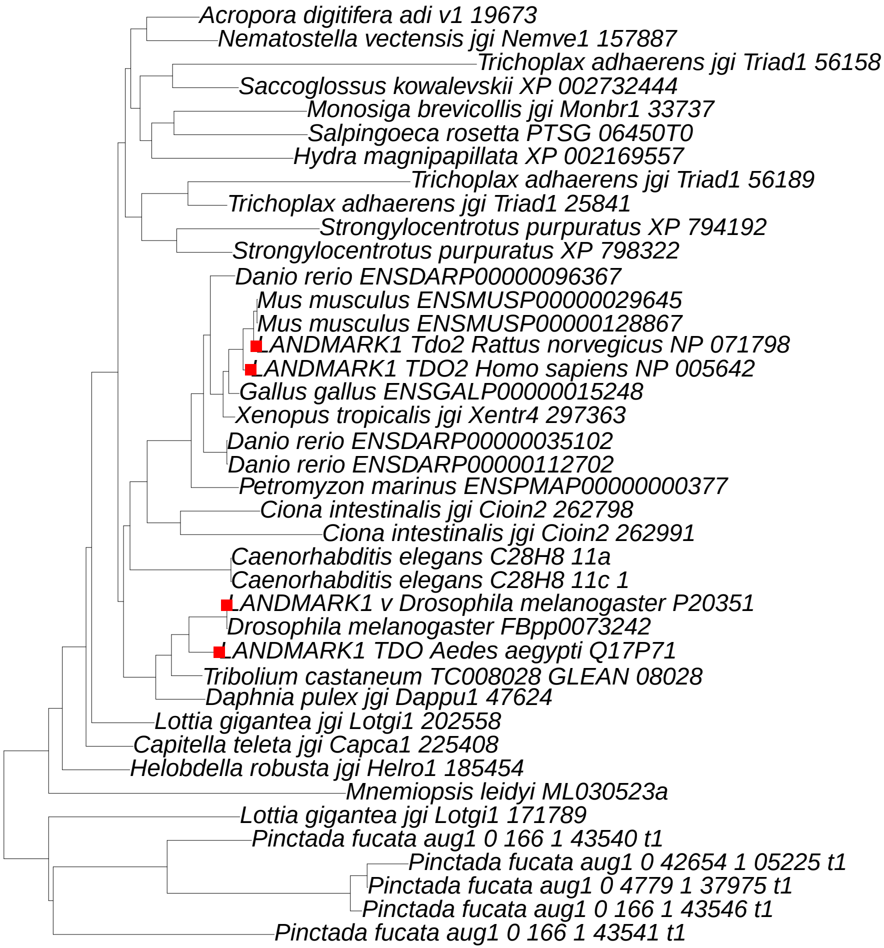

Tree File: scarlet_brown

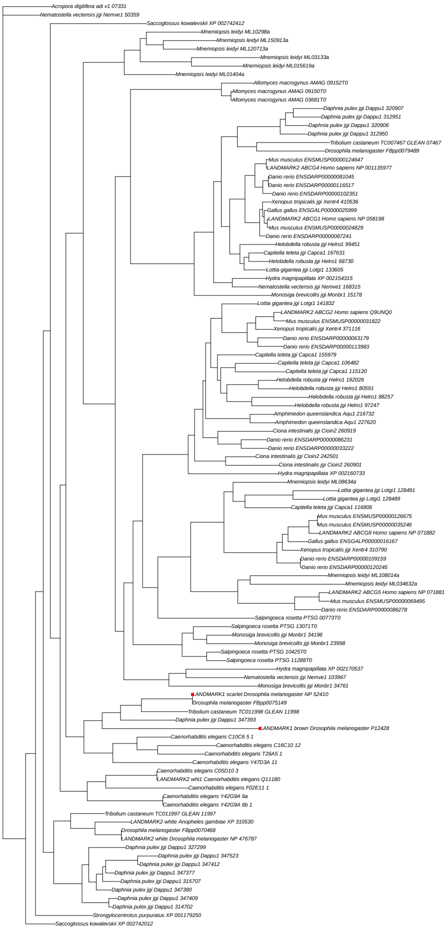

Tree File: KF

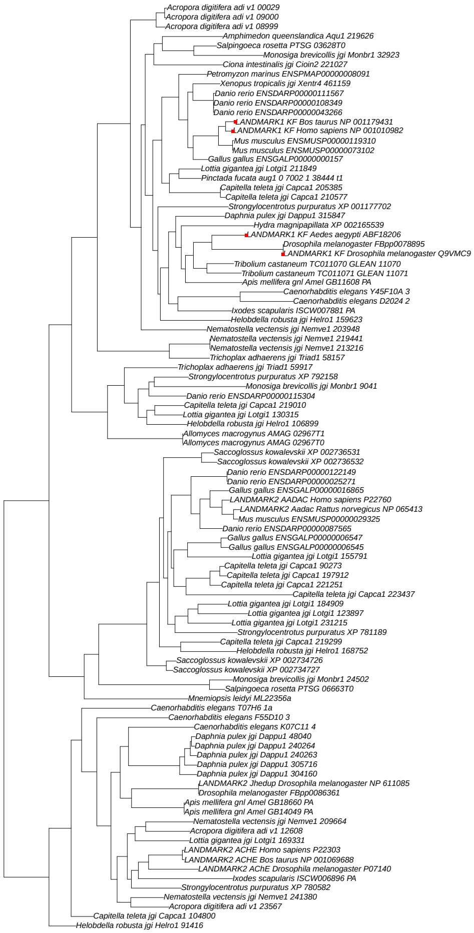
Acropora digitifera adi v1 00029
Acropora digitifera adi v1 09000
Acropora digitifera adi v1 08999
Amphimedon queenslandica Aqu1 219626
Salpingoeca rosetta PTSG 03628T0
Monosiga brevicollis jgi Monbr1 32923
Ciona intestinalis jgi Cioin2 221027
Petromyzon marinus ENSPMAP00000008091
Xenopus tropicalis jgi Xentr4 461159
Danio rerio ENSDARP00000111567
Danio rerio ENSDARP00000108349
Danio rerio ENSDARP00000043266
LANDMARK1 KF Bos taurus NP 001179431
LANDMARK1 KF Homo sapiens NP 001010982
Mus musculus ENSMUSP00000119310
Mus musculus ENSMUSP00000073102
Gallus gallus ENSGALP00000000157
Lottia gigantea jgi Lotgi1 211849
Pinctada fucata aug1 0 7002 1 38444 t1
Capitella teleta jgi Capca1 205385
Capitella teleta jgi Capca1 210577
Strongylocentrotus purpuratus XP 001177702
Daphnia pulex jgi Dappu1 315847
Hydra magnipapillata XP 002165539
LANDMARK1 KF Aedes aegypti ABF18206
Drosophila melanogaster FBpp0078895
LANDMARK1 KF Drosophila melanogaster Q9VMC9
Tribolium castaneum TC011070 GLEAN 11070
Tribolium castaneum TC011071 GLEAN 11071
Apis mellifera gnl Amel GB11608 PA
Caenorhabditis elegans Y45F10A 3
Caenorhabditis elegans D2024 2
Ixodes scapularis ISCW007881 PA
Helobdella robusta jgi Helro1 159623
Nematostella vectensis jgi Nemve1 203948
Nematostella vectensis jgi Nemve1 219441
Nematostella vectensis jgi Nemve1 213216
Trichoplax adhaerens jgi Triad1 58157
Trichoplax adhaerens jgi Triad1 59917
Strongylocentrotus purpuratus XP 792158
Monosiga brevicollis jgi Monbr1 9041
Danio rerio ENSDARP00000115304
Capitella teleta jgi Capca1 219010
Lottia gigantea jgi Lotgi1 130315
Helobdella robusta jgi Helro1 106899
Allomyces macrogynus AMAG 02967T1
Allomyces macrogynus AMAG 02967T0
Saccoglossus kowalevskii XP 002736531
Saccoglossus kowalevskii XP 002736532
Danio rerio ENSDARP00000122149
Danio rerio ENSDARP00000025271
Gallus gallus ENSGALP00000016865
LANDMARK2 AADAC Homo sapiens P22760
LANDMARK2 Aadac Rattus norvegicus NP 065413
Mus musculus ENSMUSP00000029325
Danio rerio ENSDARP00000087565
Gallus gallus ENSGALP00000006547
Gallus gallus ENSGALP00000006545
Lottia gigantea jgi Lotgi1 155791
Capitella teleta jgi Capca1 90273
Capitella teleta jgi Capca1 197912
Capitella teleta jgi Capca1 221251
Capitella teleta jgi Capca1 223437
Lottia gigantea jgi Lotgi1 184909
Lottia gigantea jgi Lotgi1 123897
Lottia gigantea jgi Lotgi1 231215
Strongylocentrotus purpuratus XP 781189
Capitella teleta jgi Capca1 219299
Helobdella robusta jgi Helro1 168752
Saccoglossus kowalevskii XP 002734726
Saccoglossus kowalevskii XP 002734727
Monosiga brevicollis jgi Monbr1 24502
Salpingoeca rosetta PTSG 06663T0
Mnemiopsis leidyi ML22356a
Caenorhabditis elegans T07H6 1a
Caenorhabditis elegans F55D10 3
Caenorhabditis elegans K07C11 4
Daphnia pulex jgi Dappu1 48040
Daphnia pulex jgi Dappu1 240264
Daphnia pulex jgi Dappu1 240263
Daphnia pulex jgi Dappu1 305716
Daphnia pulex jgi Dappu1 304160
LANDMARK2 Jhedup Drosophila melanogaster NP 611085
Drosophila melanogaster FBpp0086361
Apis mellifera gnl Amel GB18660 PA
Apis mellifera gnl Amel GB14049 PA
Nematostella vectensis jgi Nemve1 209664
Acropora digitifera adi v1 12608
Lottia gigantea jgi Lotgi1 169331
LANDMARK2 ACHE Homo sapiens P22303
LANDMARK2 ACHE Bos taurus NP 001069688
LANDMARK2 AChE Drosophila melanogaster P07140
Ixodes scapularis ISCW006896 PA
Strongylocentrotus purpuratus XP 780582
Nematostella vectensis jgi Nemve1 241380
Acropora digitifera adi v1 23567
Capitella teleta jgi Capca1 104800
Helobdella robusta jgi Helro1 91416

Tree File: KMO

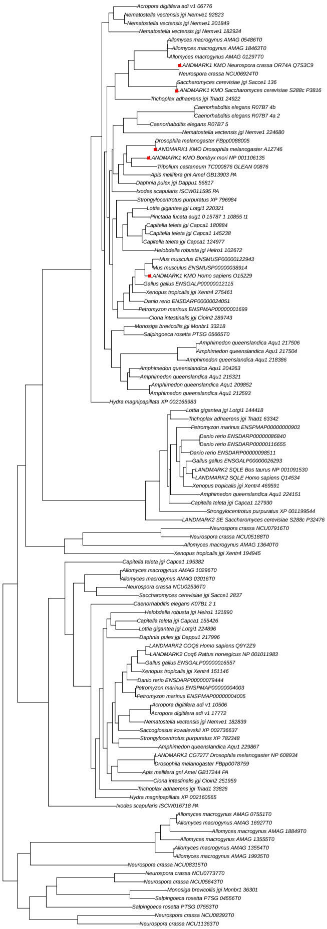

Tree File: white

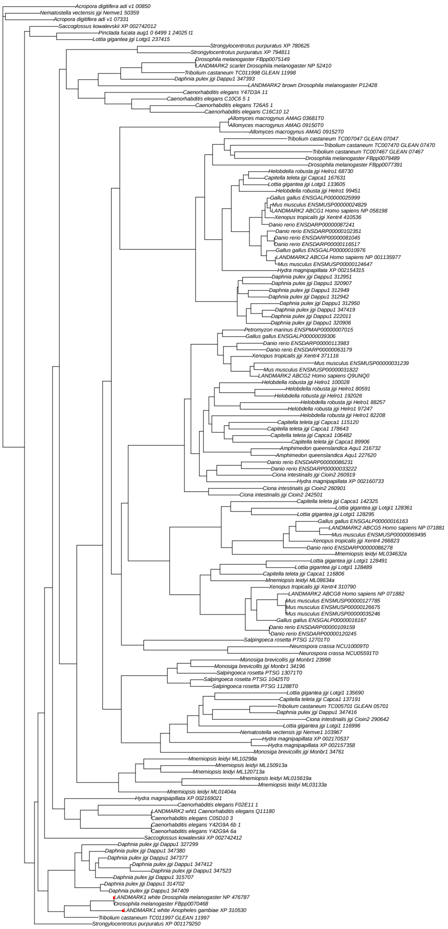

Tree File: Mitf

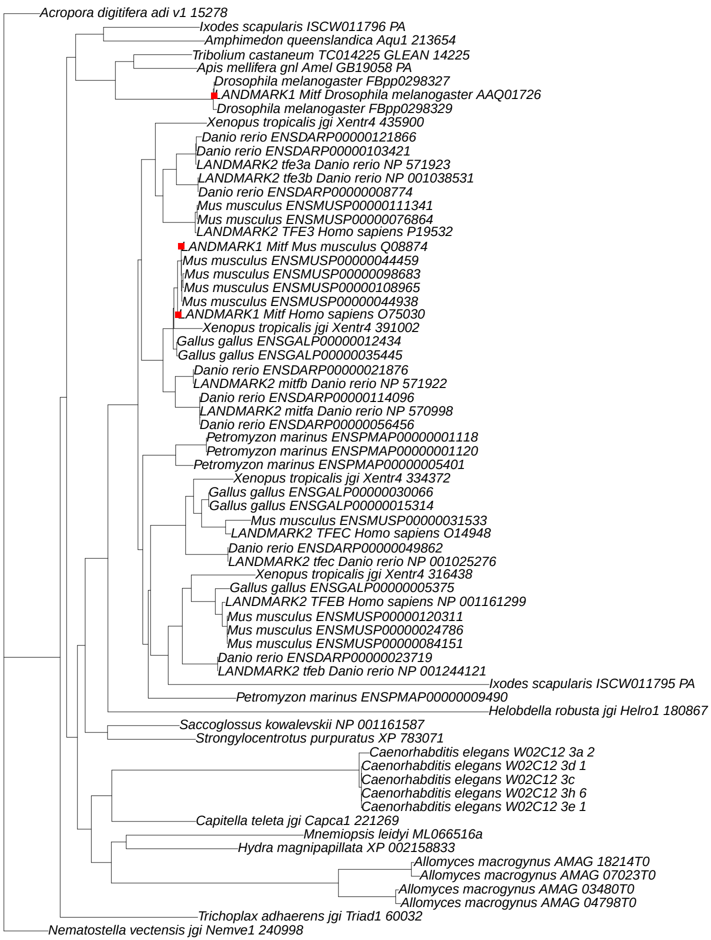

Tree File: Uros

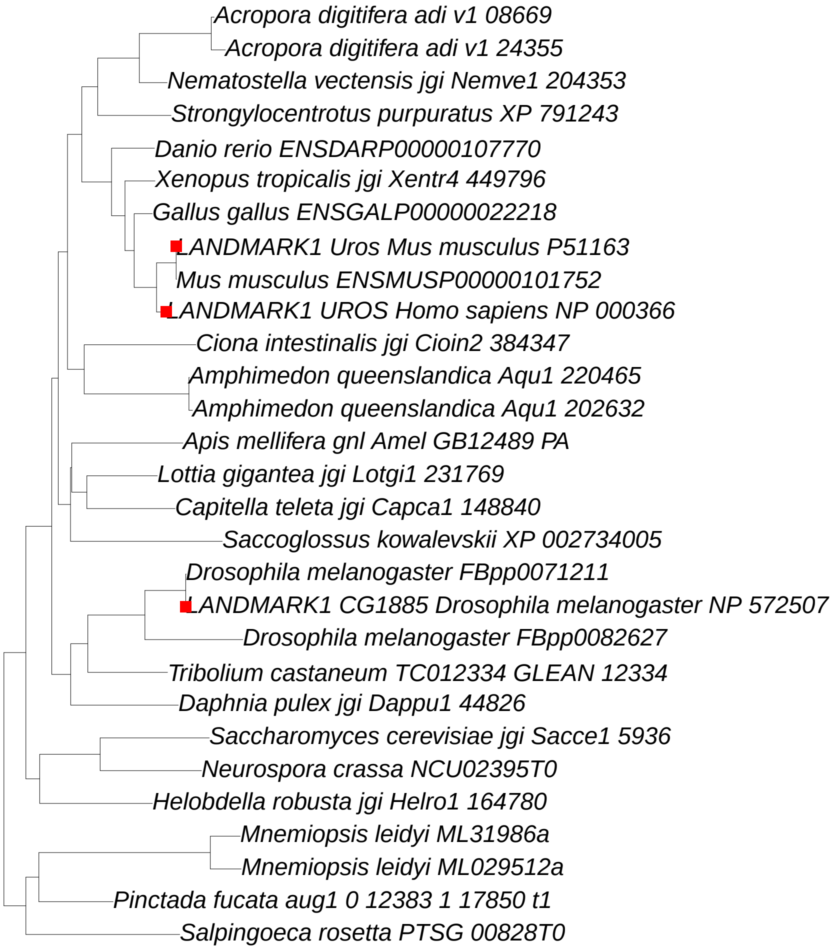

Tree File: Ppox

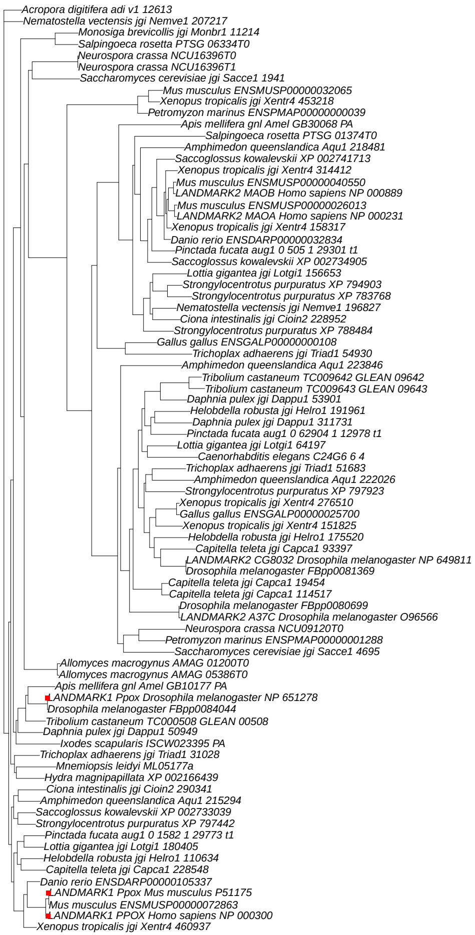

Tree File: Alas

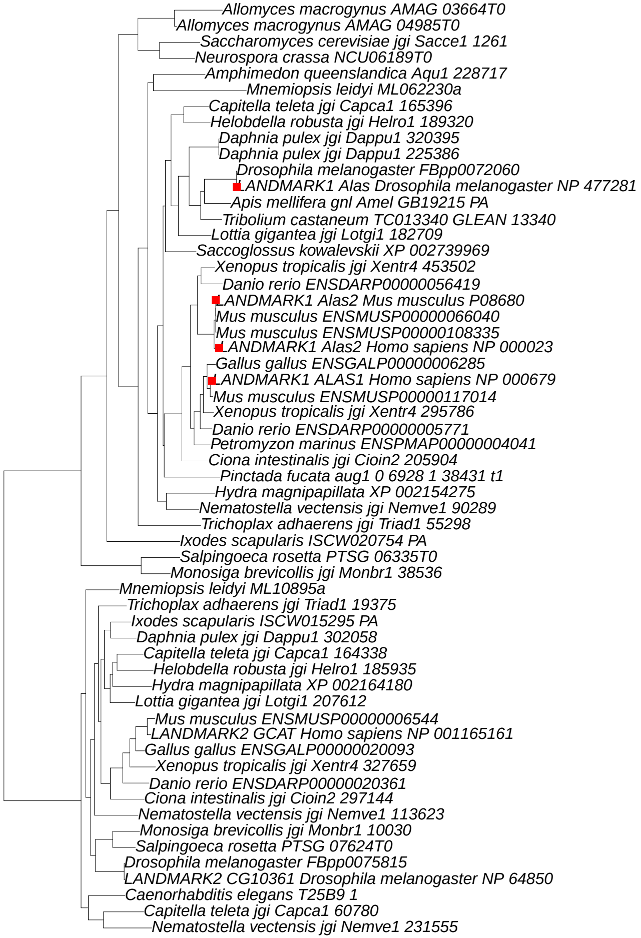

Tree File: Cpox

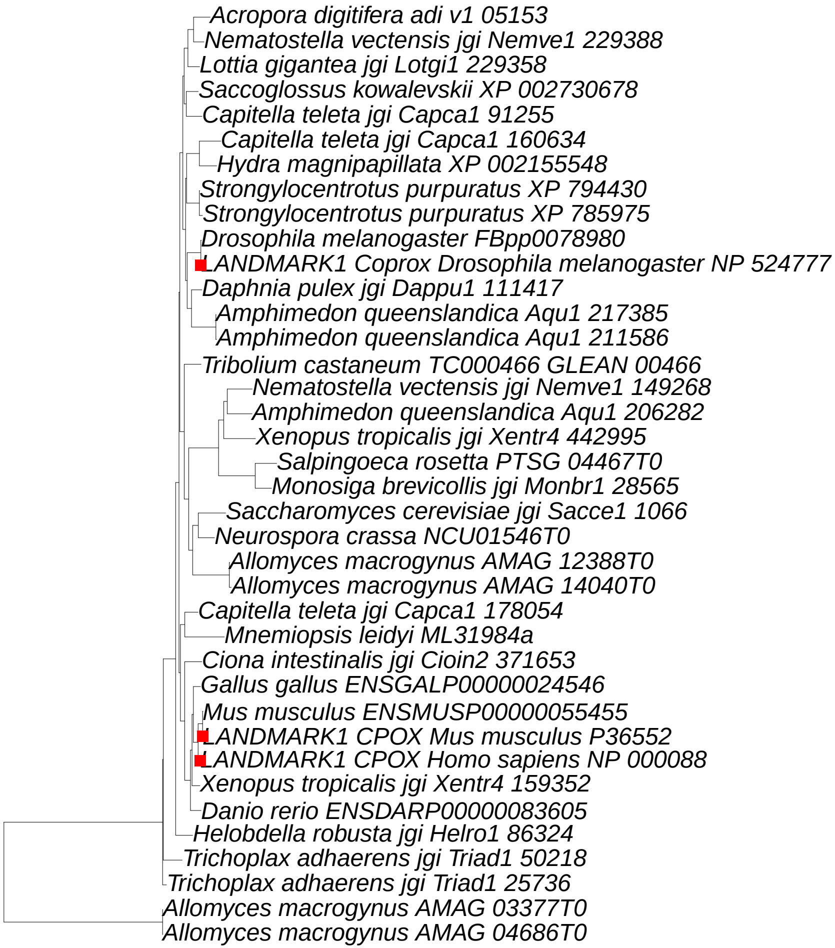

Tree File: Fech

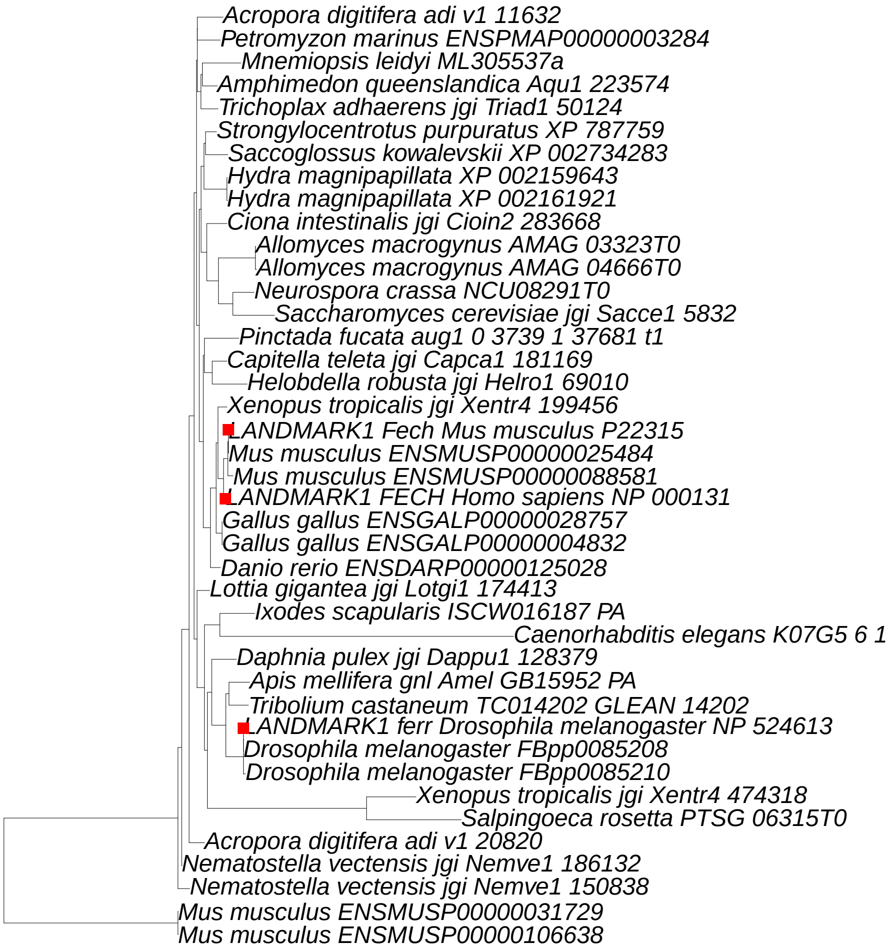

Tree File: Alad

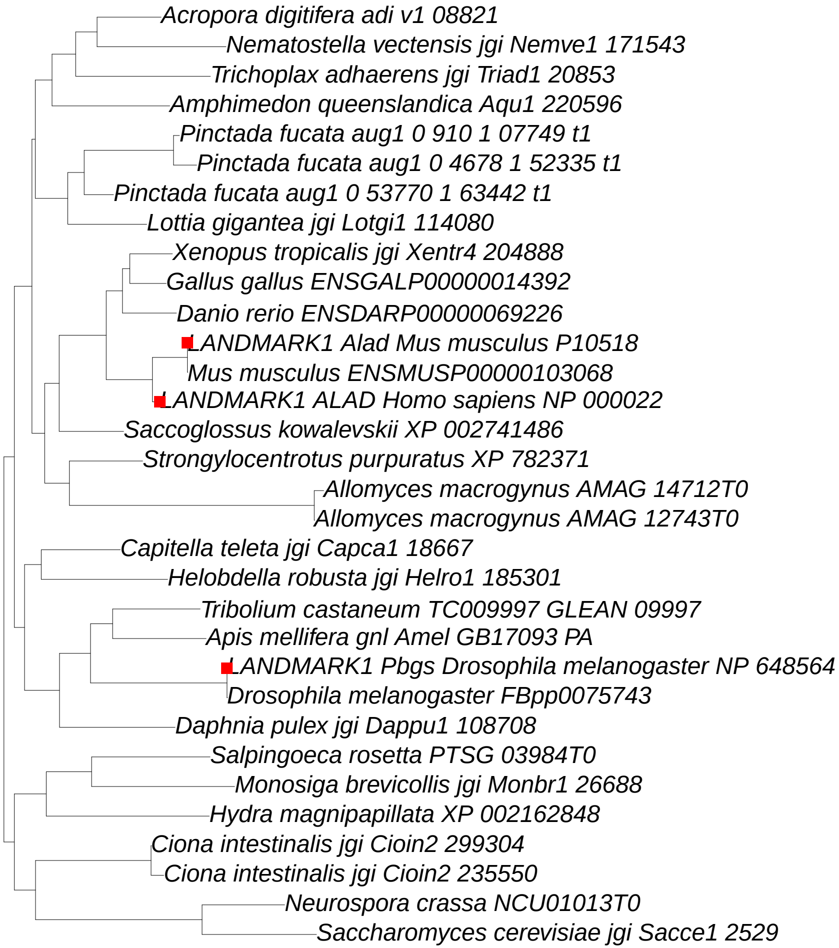

Tree File: Pbgd

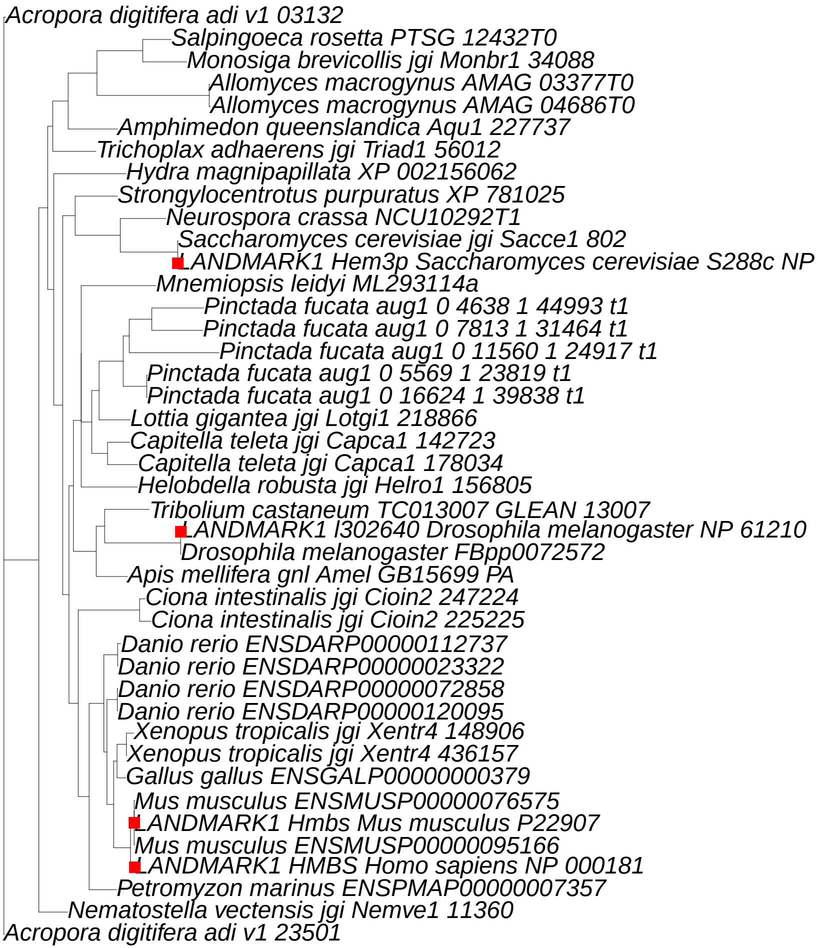

Tree File: Urod

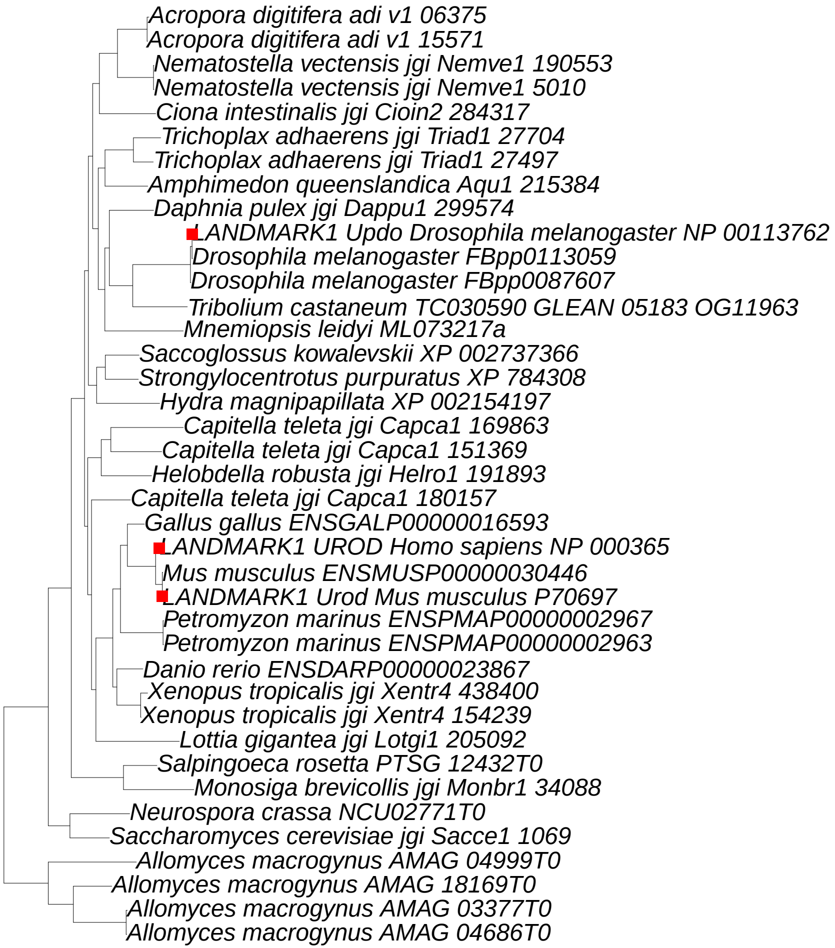

Tree File: reflectin_1a

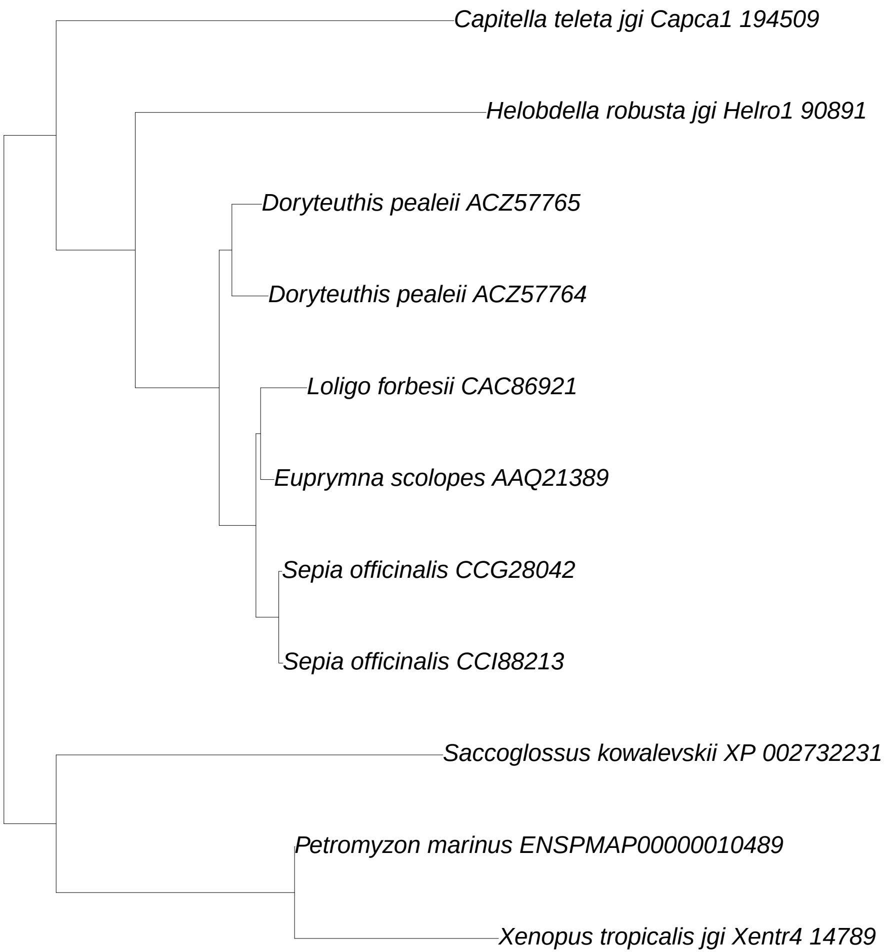

Tree File: A_crys

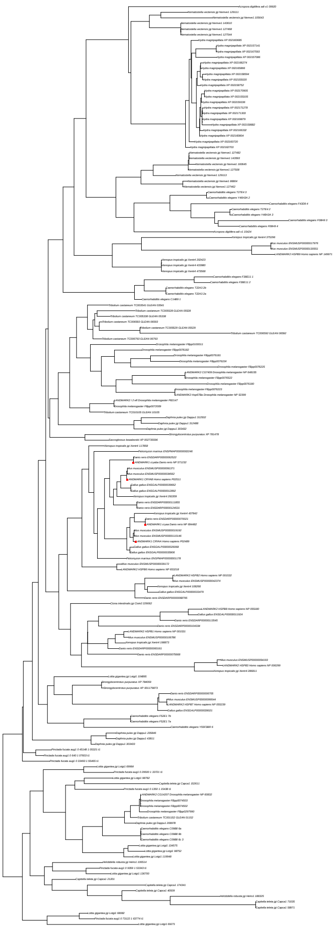

Tree File: J1_crys

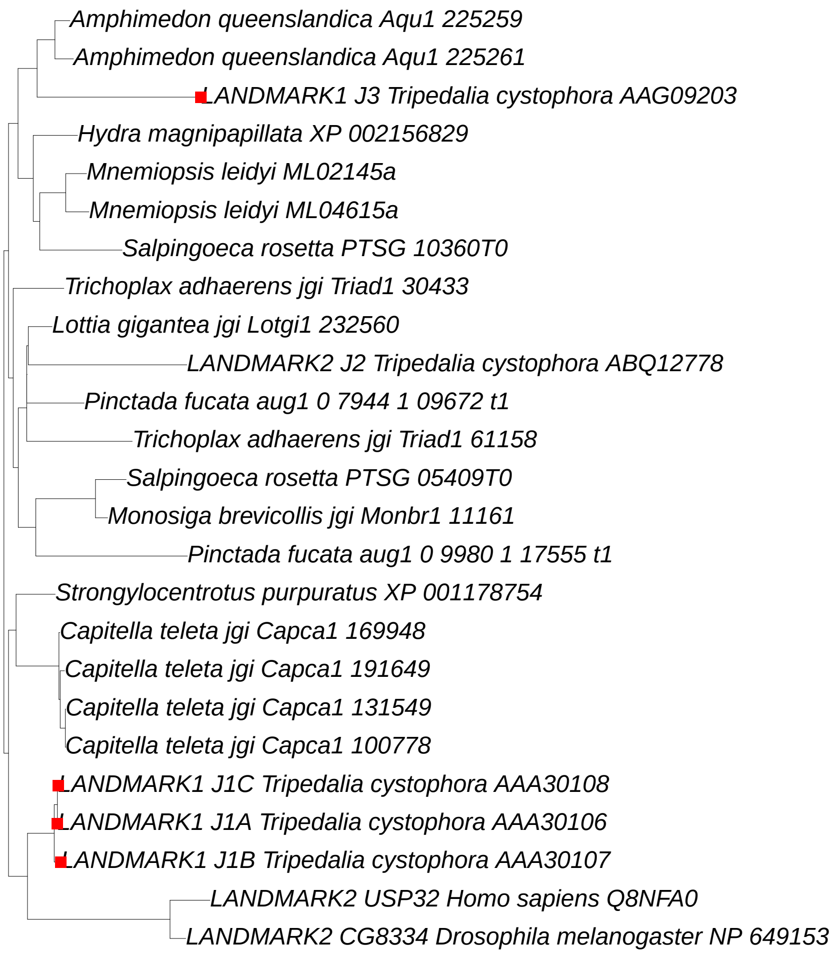

Tree File: S_crys

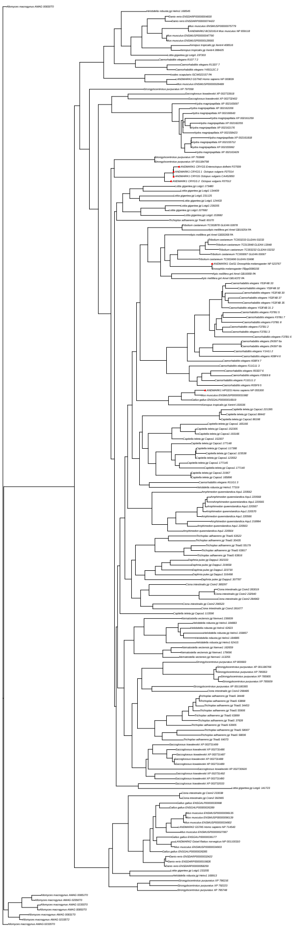

Tree File: B_crys

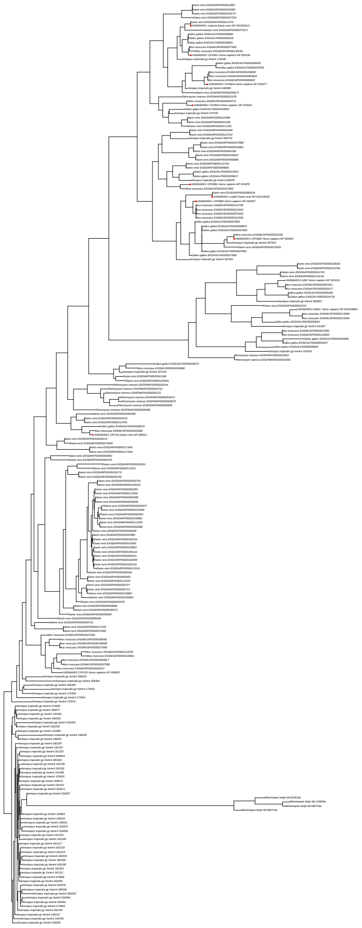

Tree File: O_crys

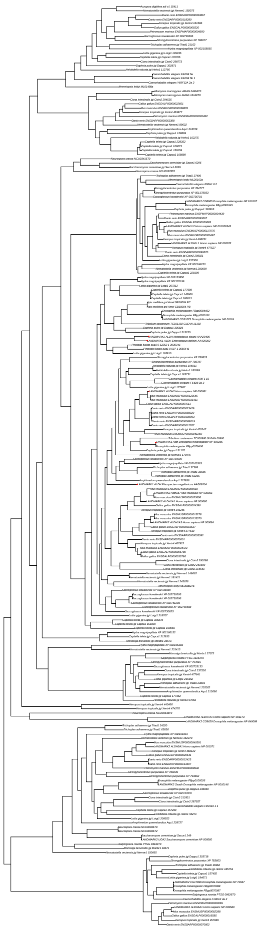

Tree File: D_crys

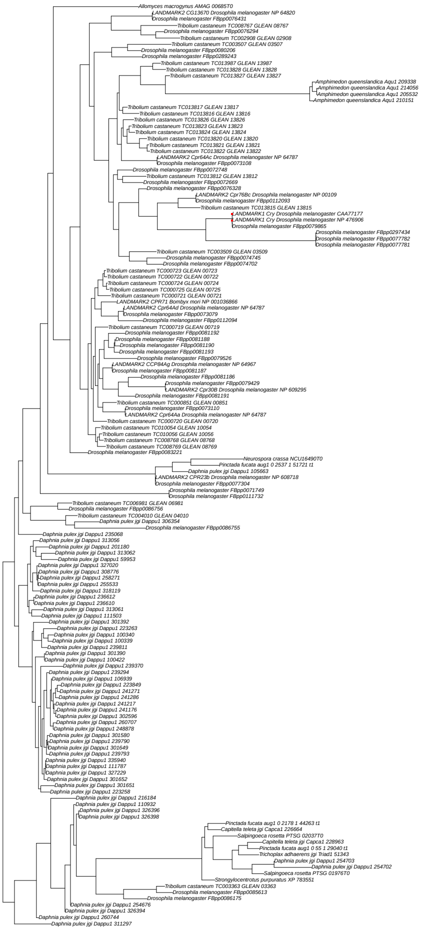

Tree File: period

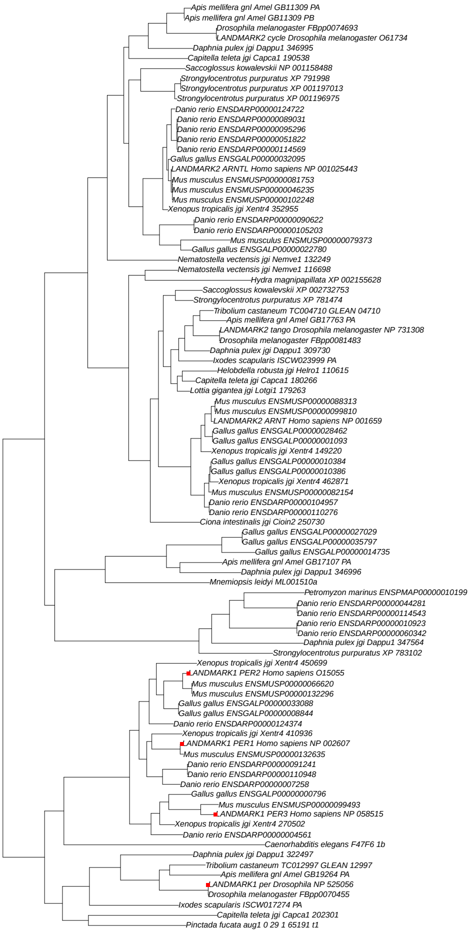

Tree File: cwo

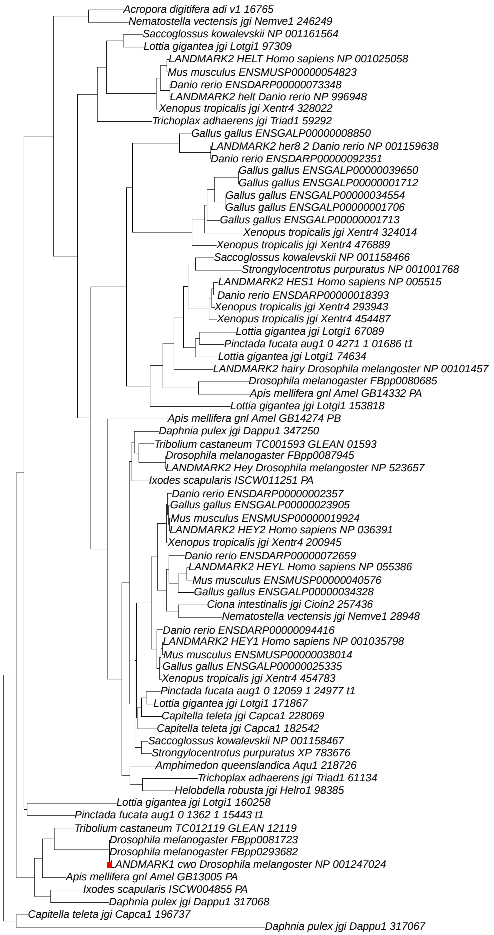

Tree File: cycle

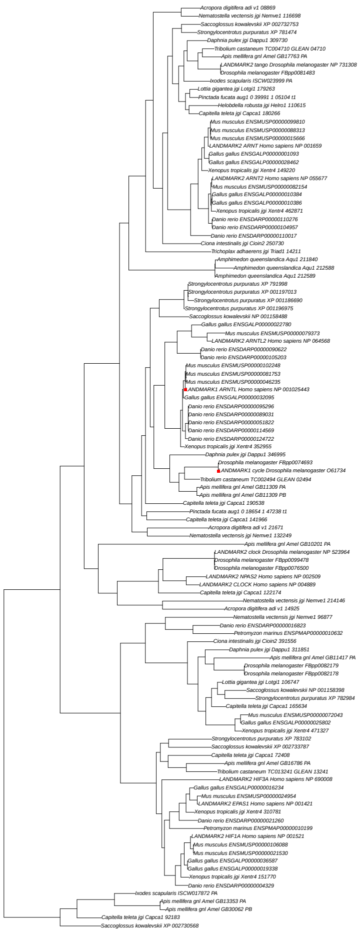

Tree File: lark

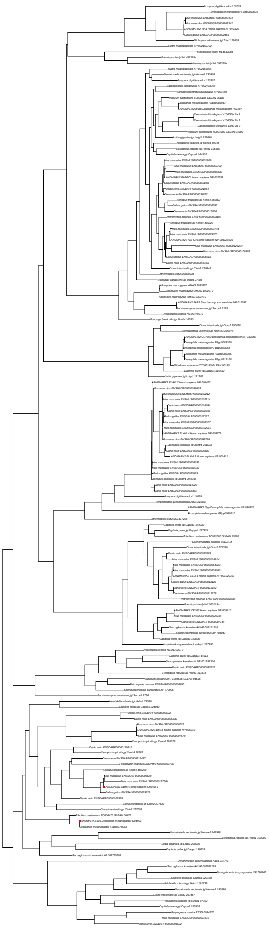

Tree File: vrille

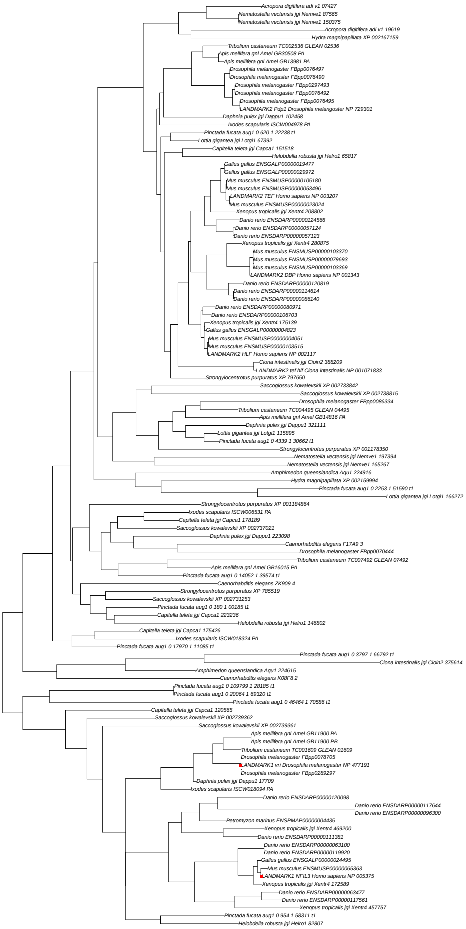

Tree File: pdf

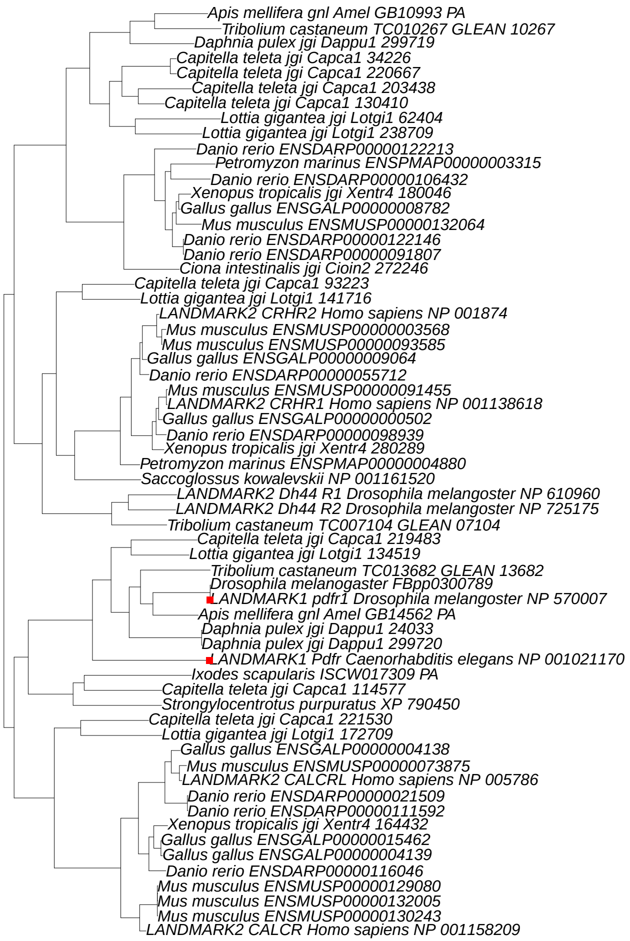

Tree File: par

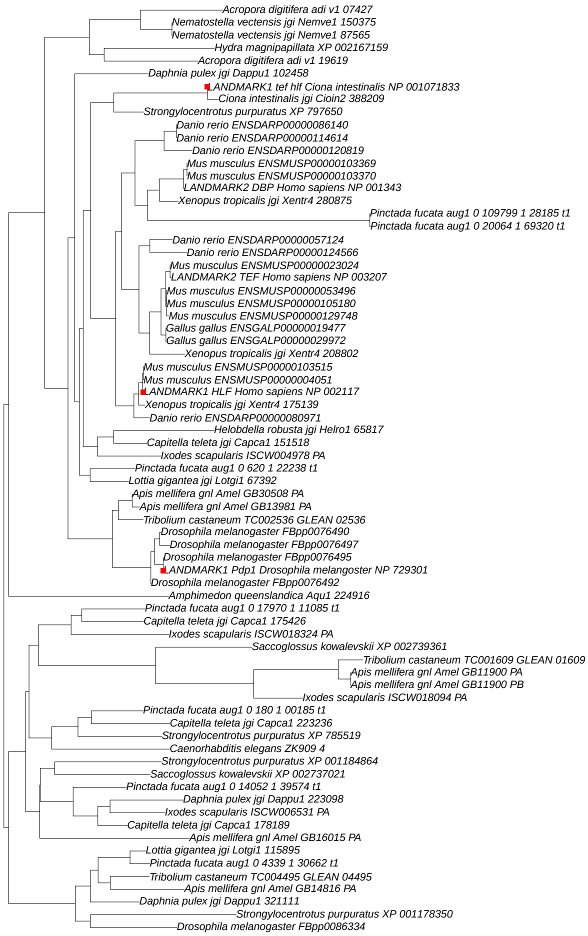

Tree File: slo

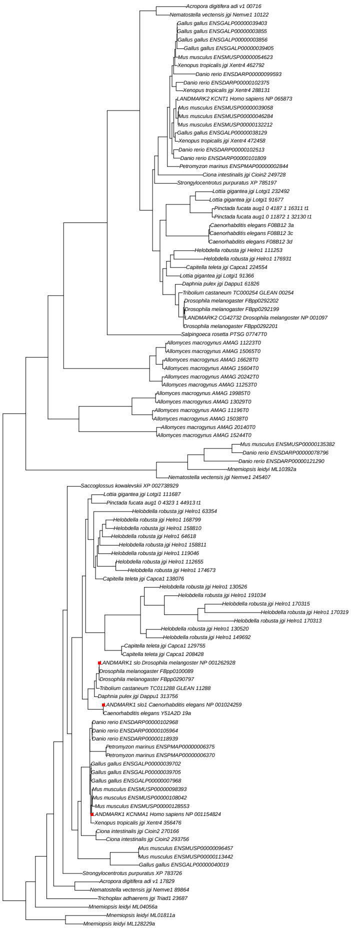

Tree File: cry2

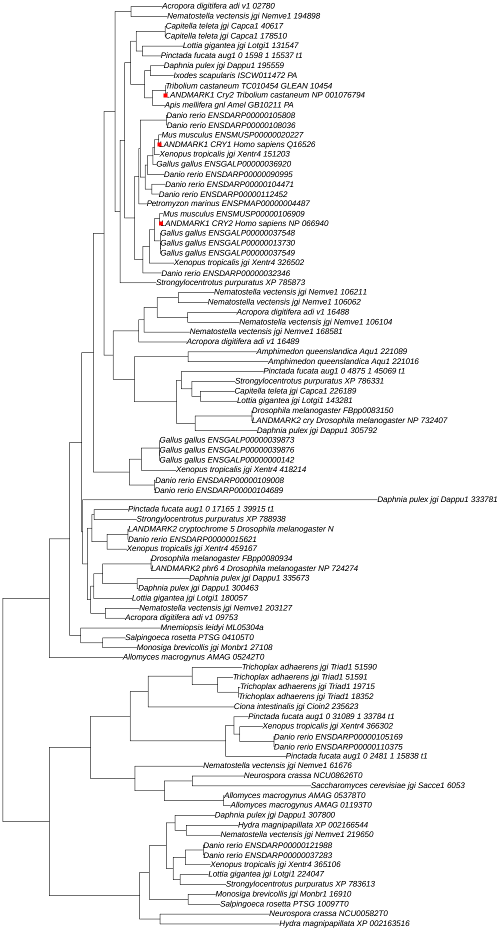

Tree File: timeless

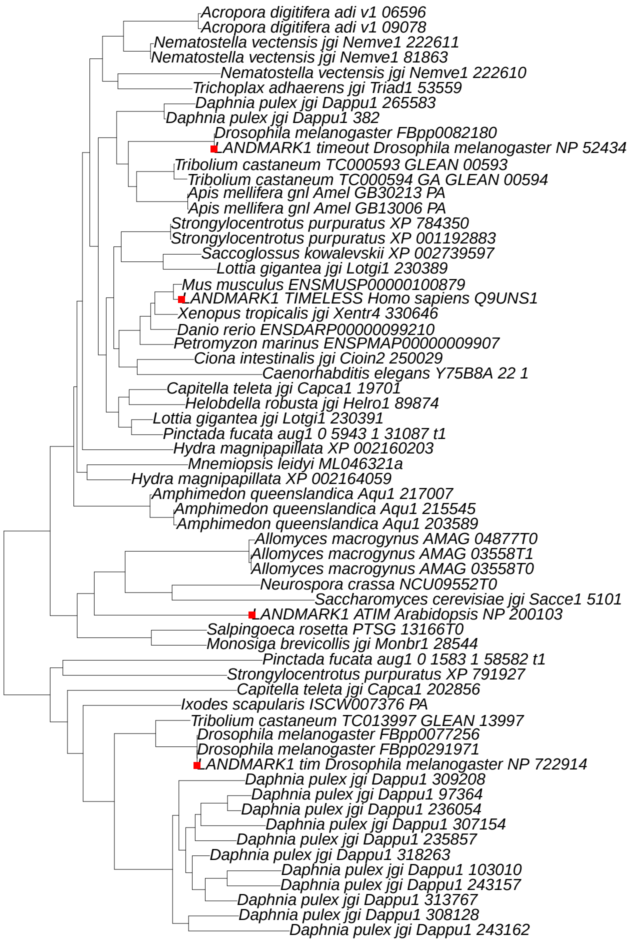

Tree File: clock

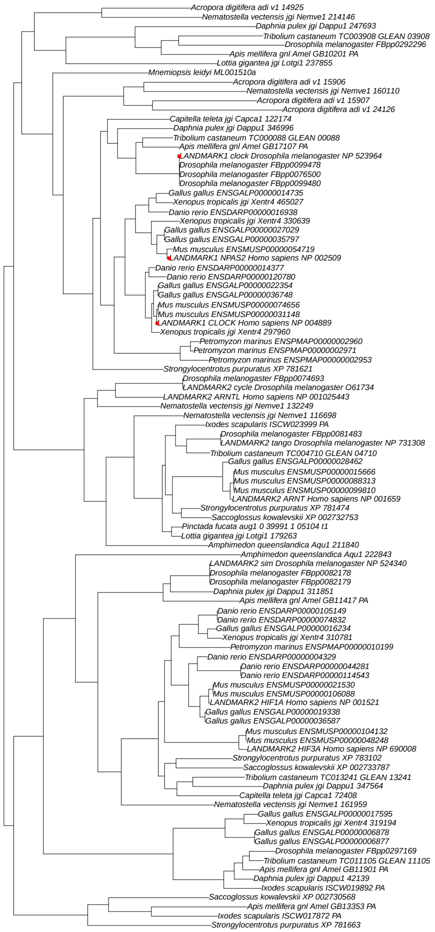

Tree File: tango

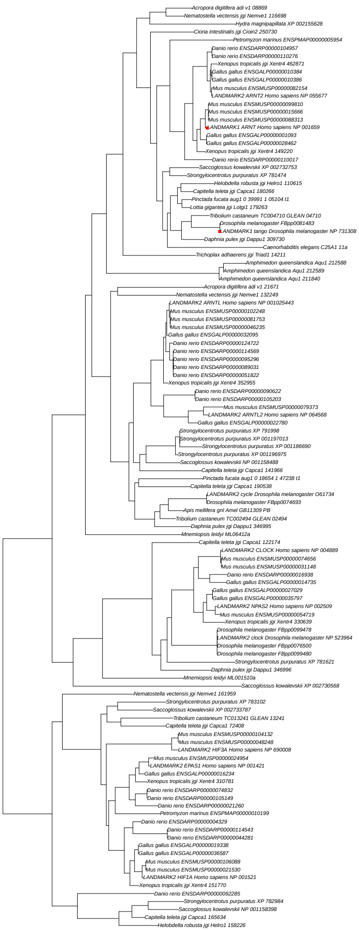

Supplement: Additional file 2: — LIT PIA trees. [file 12859_2014_350_MOESM2_ESM.pdf]
