# Supplementary material for: Using phylogenetically-informed annotation (PIA) to search for light-interacting genes in transcriptomes from non-model organisms
Source: BMC Bioinformatics. 2014 Nov 19;15(1):350. doi: 10.1186/s12859-014-0350-x (PMC4255452; doi:10.1186/s12859-014-0350-x)
Supplement: Additional file 5: — Supplementary References for Table S3. [file 12859_2014_350_MOESM5_ESM.docx]

**SUPPLEMENTARY REFERENCES**

1. Shinzato C, Shoguchi E, Kawashima T, Hamada M, Hisata K, Tanaka M, Fujie M, Fujiwara M, Koyanagi R, Ikuta T: **Using the *Acropora digitifera* genome to understand coral responses to environmental change**. *Nature* 2011, **476**:320-323.

2. Ruiz-Trillo I, Burger G, Holland PWH, King N, Lang BF, Roger AJ, Gray MW: **The origins of multicellularity: a multi-taxon genome initiative**. *Trends in Genetics* 2007, **23**:113-118.

3. Srivastava M, Simakov O, Chapman J, Fahey B, Gauthier ME, Mitros T, Richards GS, Conaco C, Dacre M, Hellsten U: **The *Amphimedon queenslandica* genome and the evolution of animal complexity**. *Nature* 2010, **466**:720-726.

4. Weinstock GM, Robinson GE, Gibbs RA, Worley KC, Evans JD, Maleszka R, Robertson HM, Weaver DB, Beye M, Bork P: **Insights into social insects from the genome of the honeybee *Apis mellifera***. *Nature* 2006, **443**:931-949.

5. Simakov O, Marletaz F, Cho S-J, Edsinger-Gonzales E, Havlak P, Hellsten U, Kuo D-H, Larsson T, Lv J, Arendt D: **Insights into bilaterian evolution from three spiralian genomes**. *Nature* 2013, **493**:526–531.

6. Consortium TCeS: **Genome Sequence of the Nematode *C. elegans*: A Platform for Investigating Biology**. *Science* 1998, **282**:2012-2018.

7. Dehal P, Satou Y, Campbell RK, Chapman J, Degnan B, De Tomaso A, Davidson B, Di Gregorio A, Gelpke M, Goodstein DM: **The draft genome of *Ciona intestinalis*: insights into chordate and vertebrate origins**. *Science* 2002, **298**:2157-2167.

8. Haffter P, Granato M, Brand M, Mullins MC, Hammerschmidt M, Kane DA, Odenthal J, Van Eeden F, Jiang Y-J, Heisenberg C-P: **The identification of genes with unique and essential functions in the development of the zebrafish, *Danio rerio***. *Development* 1996, **123**:1-36.

9. Colbourne JK, Pfrender ME, Gilbert D, Thomas WK, Tucker A, Oakley TH, Tokishita S, Aerts A, Arnold GJ, Basu MK: **The ecoresponsive genome of *Daphnia pulex***. *Science* 2011, **331**:555-561.

10. Adams MD, Celniker SE, Holt RA, Evans CA, Gocayne JD, Amanatides PG, Scherer SE, Li PW, Hoskins RA, Galle RF: **The genome sequence of *Drosophila melanogaster***. *Science* 2000, **287**:2185-2195.

11. Hillier LW, Miller W, Birney E, Warren W, Hardison RC, Ponting CP, Bork P, Burt DW, Groenen MA, Delany ME: **Sequence and comparative analysis of the chicken genome provide unique perspectives on vertebrate evolution**. *Nature* 2004, **432**(7018):695-716.

12. Chapman JA, Kirkness EF, Simakov O, Hampson SE, Mitros T, Weinmaier T, Rattei T, Balasubramanian PG, Borman J, Busam D: **The dynamic genome of *Hydra***. *Nature* 2010, **464**:592-596.

13. Lawson D, Arensburger P, Atkinson P, Besansky NJ, Bruggner RV, Butler R, Campbell KS, Christophides GK, Christley S, Dialynas E: **VectorBase: a data resource for invertebrate vector genomics**. *Nucleic Acids Research* 2009, **37**:D583-D587.

14. Ryan JF, Pang K, Schnitzler CE, Nguyen A-D, Moreland RT, Simmons DK, Koch BJ, Francis WR, Havlak P, Smith SA: **The genome of the ctenophore *Mnemiopsis leidyi* and its implications for cell type evolution**. *Science* 2013, **342**:1242592.

15. King N, Westbrook MJ, Young SL, Kuo A, Abedin M, Chapman J, Fairclough S, Hellsten U, Isogai Y, Letunic I: **The genome of the choanoflagellate *Monosiga brevicollis* and the origin of metazoans**. *Nature* 2008, **451**:783-788.

16. Chinwalla AT, Cook LL, Delehaunty KD, Fewell GA, Fulton LA, Fulton RS, Graves TA, Hillier LW, Mardis ER, McPherson JD: **Initial sequencing and comparative analysis of the mouse genome**. *Nature* 2002, **420**:520-562.

17. Putnam NH, Srivastava M, Hellsten U, Dirks B, Chapman J, Salamov A, Terry A, Shapiro H, Lindquist E, Kapitonov VV: **Sea anemone genome reveals ancestral eumetazoan gene repertoire and genomic organization**. *Science* 2007, **317**:86-94.

18. Galagan JE, Calvo SE, Borkovich KA, Selker EU, Read ND, Jaffe D, FitzHugh W, Ma L-J, Smirnov S, Purcell S: **The genome sequence of the filamentous fungus *Neurospora crassa***. *Nature* 2003, **422**:859-868.

19. Smith JJ, Kuraku S, Holt C, Sauka-Spengler T, Jiang N, Campbell MS, Yandell MD, Manousaki T, Meyer A, Bloom OE: **Sequencing of the sea lamprey (*Petromyzon marinus*) genome provides insights into vertebrate evolution**. *Nature Genetics* 2013, **45**:415-421.

20. Takeuchi T, Kawashima T, Koyanagi R, Gyoja F, Tanaka M, Ikuta T, Shoguchi E, Fujiwara M, Shinzato C, Hisata K: **Draft genome of the pearl oyster *Pinctada fucata*: a platform for understanding bivalve biology**. *DNA Research* 2012, **19**:117-130.

21. Mewes H, Albermann K, Bähr M, Frishman D, Gleissner A, Hani J, Heumann K, Kleine K, Maierl A, Oliver S: **Overview of the yeast genome**. *Nature* 1997, **387**:7-8.

22. Freeman R, Wu M, Cordonnier-Pratt M, Pratt L, Gruber C, Smith M, Lander E, Stange-Thomann N, Lowe C, Gerhart J: **cDNA sequences for transcription factors and signaling proteins of the hemichordate *Saccoglossus kowalevskii*: efficacy of the expressed sequence tag (EST) approach for evolutionary and developmental studies of a new organism**. *The Biological Bulletin* 2008, **214**(3):284-302.

23. Fairclough SR, Chen Z, Kramer E, Zeng Q, Young S, Robertson HM, Begovic E, Richter DJ, Russ C, Westbrook MJ: **Premetazoan genome evolution and the regulation of cell differentiation in the choanoflagellate *Salpingoeca rosetta***. *Genome Biol* 2013, **14**:R15.

24. Sodergren E, Weinstock GM, Davidson EH, Cameron RA, Gibbs RA, Angerer RC, Angerer LM, Arnone MI, Burgess DR, Burke RD: **The genome of the sea urchin *Strongylocentrotus purpuratus***. *Science* 2006, **314**:941-952.

25. Richards S, Gibbs RA, Weinstock GM, Brown SJ, Denell R, Beeman RW, Gibbs R, Bucher G, Friedrich M, Grimmelikhuijzen CJ: **The genome of the model beetle and pest *Tribolium castaneum***. *Nature* 2008, **452**:949-955.

26. Srivastava M, Begovic E, Chapman J, Putnam NH, Hellsten U, Kawashima T, Kuo A, Mitros T, Salamov A, Carpenter ML: **The *Trichoplax* genome and the nature of placozoans**. *Nature* 2008, **454**:955-960.

27. Hellsten U, Harland RM, Gilchrist MJ, Hendrix D, Jurka J, Kapitonov V, Ovcharenko I, Putnam NH, Shu S, Taher L: **The genome of the Western clawed frog *Xenopus tropicalis***. *Science* 2010, **328**:633-636.
